# Multidimensional therapeutic advantages of *Smilax glabra* (*Tufuling*)-containing formulae in gout: an integrated Systematic Review and network pharmacology-based prediction

**DOI:** 10.3389/fendo.2026.1863098

**Published:** 2026-07-01

**Authors:** Qiaoyun Liu, Xiuming Li, Yuping Lin, Xianyu Tang, Guanjie Fan, Lu Sun

**Affiliations:** 1 The Second Clinical College of Guangzhou University of Chinese Medicine, Guangzhou, China; 2 Guangdong Provincial Hospital of Chinese Medicine, Guangzhou, China; 3 The Second Affiliated Hospital of Guangzhou University of Chinese Medicine, Guangzhou, China

**Keywords:** anti-inflammation, gout, network pharmacology, Smilax glabra (Tufuling), systematic review, uric acid regulation

## Abstract

**Background:**

Gout, an inflammatory form of arthritis triggered by monosodium urate (MSU) crystal deposition, poses a substantial global health burden with increasing prevalence and younger onset, particularly in China. In traditional Chinese medicine (TCM), dampness-heat accumulation is a predominant pattern associated with gout. *Smilax glabra* (*Tufuling*), a medicinal and edible herb with a history of use for detoxification and elimination of dampness, is also known to promote joint mobility. It is widely used for these purposes. This study aimed to systematically evaluate the clinical efficacy and safety of *Tufuling*-containing TCM formulae—typically used in combination with other Chinese herbs and/or Western medicine—for gout patients with dampness-heat accumulation, and to generate testable mechanistic hypotheses using network pharmacology.

**Methods:**

A systematic review (SR) and meta-analysis were conducted by searching PubMed, Embase, CNKI, and other databases from inception to June 2025, including randomized controlled trials (RCTs) of formulae containing *Tufuling* interventions for gout. Network pharmacology was utilized to identify active compounds, target genes, and key pathways involved in gout treatment, followed by molecular docking to generate mechanistic hypotheses.

**Results:**

A total of 56 RCTs involving 4,605 participants were included in this analysis. A meta-analysis revealed that *Tufuling*-containing formulae, particularly when combined with Western medicine (WM) or administered as comprehensive TCM therapy, were associated with reductions in, visual analog scale (VAS) scores (pain), serum uric acid (UA) levels, C-reactive protein (CRP) levels, and the erythrocyte sedimentation rate (ESR) compared with WM monotherapy. A lower reported incidence of gastrointestinal adverse events was observed (73 vs. 161 cases); however, adverse event reporting was incomplete, treatment durations were short, and the follow-up data were limited. Meta-analysis suggested that simpler interventions may be associated with fewer adverse events. Network pharmacology predicted 11, 3, and 14 active compounds for *Tufuling*, *Huangbo*, and *Bixie*, respectively. Target mapping predicted 49 targets for *Tufuling*, 81 for the *Tufuling*–*Huangbo* pair, and 7 for *Bixie*—all nested within the *Tufuling* target set. The *Tufuling* PPI network (48 nodes, 348 edges) identified four core targets: PTGS2, IL1B, PPARG, and TP53. The *Tufuling*–*Huangbo* PPI network (76 nodes, 1,054 edges) yielded seven core targets; four overlapped with *Tufuling*, while CCL2, BCL2, and CXCL8 were *Huangbo*-specific. KEGG analysis of 48 *Tufuling* targets identified 228 pathways, with key enrichment in metabolism, lipid and atherosclerosis, PI3K–Akt, TNF, and IL-17 signaling. The 76 *Tufuling*–*Huangbo* targets revealed 233 pathways, showing enhanced enrichment in PI3K–Akt, NOD-like receptor, MAPK, TNF, and IL-17 signaling relative to *Tufuling* alone. Molecular docking predicted For the *Tufuling*, diosgenin would bound PTGS2 most strongly (−11.6 kcal/mol), followed by TP53 (−9.8kcal/mol) and IL1B (−8.0kcal/mol); beta-sitosterol would bound PPARG (−9.2kcal/mol). For the *Tufuling*–*Huangbo* pair, beta-sitosterol additionally would bound BCL2 (−7.9kcal/mol). For *Bixie*, diosgenin would bound PLA2G4A (−10.3kcal/mol) and PTGS2 (−9.9kcal/mol), while EINECS 213-897–0 would bound NR3C2 (−8.9kcal/mol).

**Conclusion:**

*Tufuling*-containing formulae may be associated with symptomatic improvements in acute gout with dampness-heat accumulation, including analgesic, anti-inflammatory, and uric acid–lowering effects, although the certainty of evidence ranges from low to moderate. The safety profile appears promising but remains inadequately characterized due to incomplete reporting and short follow-up. The efficacy of this treatment may be mediated by multiple compounds and multi-target modulation of inflammatory and metabolic pathways. *Tufuling*-based interventions have been identified as potentially valuable adjunctive therapies for the treatment of gout. However, further rigorous RCTs and experimental studies are needed to validate its long-term efficacy and mechanism of action.

**Systematic review registration:**

https://www.crd.york.ac.uk/prospero/, identifier CRD420251060498.

## 1 Introduction

Gout is triggered by the deposition of monosodium urate (MSU) crystals in the joints, bones, and soft tissues. It can manifest as one or more of the following: acute arthritis (gout flare), chronic arthritis (chronic gouty arthritis), or tophi (tophaceous gout) (1, 2).

Globally, more than 55.8 million individuals are affected by gout. Based on systematic analyses from the Global Burden of Disease (GBD) study, this figure is projected to surpass 95 million by 2050 (3).

In China, data from 2025 indicate that the prevalence of hyperuricemia among adults has reached 14%, with the total patient population exceeding 180 million. This condition demonstrates a significant sex disparity, being markedly more common in men (24.5%) than in women (3.6%) (4). Furthermore, the prevalence of gout in China ranges from 0.86% to 2.20%, corresponding to an estimated 10.23 to 26.18 million affected individuals. The disease burden exhibits distinct demographic and geographic variations, with higher rates observed in urban areas than in rural areas and in coastal regions than in inland areas (5).

A concerning trend is the shift toward a younger onset of gout, characterized by a sharp increase in the number of adolescent cases (6). This phenomenon imposes substantial medical, economic, and societal burdens on both individuals and the healthcare system (7).

The increasing global incidence of gout is closely associated with population aging, shifts in dietary patterns, and the increasing prevalence of obesity. Specific dietary risk factors include chronic consumption of high-calorie foods, excessive alcohol, and fructose-rich sugar-sweetened beverages. Adopting a balanced and nutritionally appropriate diet plays a crucial role in managing serum uric acid concentrations, thereby helping to mitigate gout flares and enhance the overall quality of life. Guided by the TCM principle of pattern differentiation and treatment determination, the implementation of tailored dietary therapy can progressively improve constitutional status and serve as an adjunctive measure to control the onset and progression of the disease (8).

In TCM, dampness-heat accumulation is considered a primary pattern in gout. The clinical manifestations include red, swollen, hot, and intensely painful joints (often described as lancinating), tenderness, fever, thirst, dark urine, a red tongue with a yellow, greasy coating, and a rapid, slippery pulse. The treatment principle is to clear heat, resolve dampness, unblock the collaterals, and alleviate pain. Commonly used Chinese herbs include *Smilax glabra* (*Tufuling*), *Phellodendron chinense* (*Huangbai*), *Lonicera japonica* (*Rendongteng*), and *Cremastra appendiculata* (*Shancigu*) (9). *Smilax glabra* (*Tufuling*) has a long history of dietary use in China and was documented in the Ming Dynasty “*Jiuhuang Bencao*” as a food substitute during famines. Since the Ming and Qing dynasties, its “mild and nontoxic” nature has made it a staple in daily diets, especially in the humid climate of southern China. In 2002, the Ministry of Health of China listed *Smilax glabra* in the “Notification on Further Regulating the Management of Health Food Ingredients” as an ingredient permissible for use in health foods (10). As a medicinal and edible herb, *Smilax glabra* is effective in removing toxins, eliminating dampness, and facilitating joint movement, making it one of the most frequently used herbs for treating gout with dampness-heat accumulation.

*Tufuling*-containing formulae are widely used for gout. Clinical evidence demonstrates that Qinpi Tongfeng Formula (QPTFF) alleviates acute gouty arthritis symptoms with analgesic efficacy non-inferior to diclofenac sodium sustained-release tablets, while outperforming it in reducing serum uric acid levels and treatment-related adverse events (11). But several critical research gaps remain. First, existing clinical studies exhibit substantial heterogeneity in sample sizes and methodological quality, leading to inconsistent efficacy reports. Second, mechanistic investigations have largely focused on isolated compounds or single pathways, resulting in a fragmented understanding of the polypharmacological nature of these herbal interventions. Third, to the best of our knowledge, no systematic review or meta-analysis has been conducted to quantitatively synthesize the clinical evidence on *Tufuling*-based therapies for acute gout with dampness-heat accumulation pattern. Fourth, the molecular mechanisms underlying the clinical efficacy of *Tufuling*-containing formulae remain poorly elucidated. This study aims to address these gaps by integrating a comprehensive systematic review with network pharmacology-based mechanistic prediction.

This study will conduct a meta-analysis of clinical trials from Chinese and English databases that investigate TCM formulae with *Smilax glabra* as the primary or secondary herb for treating patients with gout presenting with dampness-heat accumulation patterns. This study aimed to summarize the clinical evidence regarding the additional benefits and safety profiles of these formulations. Network pharmacology and bioinformatics analyses were used to explore the potential molecular mechanisms underlying the therapeutic effects of *Smilax glabra* in gout management.

## 2 Methods

### 2.1 Systematic review of clinical trials

#### 2.1.1 Methodology for systematic review of clinical trials

##### 2.1.1.1 Information sources and search strategy

A comprehensive literature search was conducted according to the Cochrane Handbook for Systematic Reviews of Interventions. Electronic databases were queried from their inception to 26 May 2025, and performed a update on 30 Jun 2025. with no filters applied. The search included major international databases, specifically PubMed, ExcerptaMedica Database (Embase), Cumulative Index of Nursing and Allied Health Literature (CINAHL), Cochrane Central Register of Controlled Trials (CENTRAL), and Allied and Complementary Medicine Database (AMED), as well as prominent Chinese repositories, including SinoMed, China National Knowledge Infrastructure (CNKI), Chongqing VIP (CQVIP), and WanFang.

The search terms were grouped into three blocks: 1) intervention (formulations containing *Tufulin*g, including oral herbal formulas, external applications, acupuncture, medicated compresses, and herbal steam or wash therapies.), 2) clinical condition (including patients with gout, specifically those during an acute attack); and 3) trial design (including randomized controlled trials).

Supplementary search techniques: to ensure thoroughness, the reference lists of pertinent reviews and all eligible primary studies were manually examined. Furthermore, major clinical trial registries, namely, Chinese Clinical Trial Registry (ChiCTR), European Union Clinical Trials Register (EU-CTR), and USA National Institutes of Health register (ClinicalTrials.gov), were screened for relevant entries. Attempts were made to contact the corresponding authors of the included studies via email or telephone to acquire missing data. Information that remained unobtainable after a 4-week follow-up period was documented as ‘not available’.

The review protocol was registered *a priori* in the PROSPERO International Prospective Register of Systematic Reviews (Registration ID: CRD420251060498).

#### 2.1.2 Study inclusion criteria

##### 2.1.2.1 Study designs

Only randomized controlled trials (RCTs) were eligible.

##### 2.1.2.2 Participants

Adults were diagnosed with gout according to the following guidelines:

- The 1977 gout classification criteria established by the ACR (12).
- The American College of Rheumatology (ACR) and the European Alliance of Associations for Rheumatology (EULAR) developed the 2015 gout classification criteria together (12).
- The Chinese Guidelines for the Diagnosis and Management of Hyperuricemia and Gout were published in 2019 (13).
- Patients presented with dampness-heat accumulation patterns in traditional Chinese medicine (TCM) (5, 9).

##### 2.1.2.3 Interventions and controls

Types of interventions:

Chinese herbal medicine (CHM), other CHM therapies (for example, acupuncture, external therapeutic methods of TCM, including medicated compresses and herbal steam/wash treatments) Integrative medicine, such as CHM plus medications for acute gout attacks, has also been investigated. All interventions included *Tufuling*. In traditional Chinese medicine formulae, *Tufuling* serves as the *jun* 君 (monarch) or *chen*臣 (minister) herb. If the prescription does not explicitly specify the roles of the monarch, minister, assistant, or courier herbs, *Tufuling* will be the herb with the highest dosage in that formula. Treatment duration (≤14 days).

Types of comparators:

The conventional therapies recommended in guidelines include pharmacotherapy (medications for acute gout attacks, such as nonsteroidal anti-inflammatory drugs (NSAIDs), colchicine, corticosteroids, and urate-lowering therapy (ULT)).), diet therapy and lifestyle interventions.

##### 2.1.2.4 Outcomes

The primary outcome measures were as follows:

- Visual analog scale (VAS).
- Adverse events (AEs).

The secondary outcomes were as follows:

- Effective rate.
- Uric acid (UA).
- C-reactive protein (CRP).
- Erythrocyte sedimentation rate (ESR).
- White blood cell (WBC) count.
- TCM symptom score.

#### 2.1.3 Study exclusion criteria

- Quasirandomized controlled trials;
- Gout-related complications and comorbidities;
- Patients with isolated hyperuricemia;
- Enrolled patients did not present with dampness–heat accumulation pattern in TCM;
- *Tufuling* serves neither as the *jun*君 (monarch) herb, the *chen*臣 (minister) herb, nor as the herb with the highest dosage in the formula;
- Integrative medicine studies that used different Western medicine therapies in the intervention group compared to the control group;
- The control group was treated with a form of Chinese medicine.

#### 2.1.4 Database search and study selection

Systematic literature retrieval was done across key biomedical databases in English and Chinese. We followed the guidelines from the Cochrane Handbook for Systematic Reviews of Interventions (14). The electronic searches included data from the start of the databases up to June 2025, with no restrictions.

The international databases searched were PubMed, Embase, CINAHL, CENTRAL, and AMED. For Chinese literature, we used the SinoMed, CNKI, CQVIP, and Wanfang databases.

The search strategy focused on four main areas: 1) interventions using the medicinal herb *Smilax glabra* (*Tufuling*), including oral herbal formulas, external applications, acupuncture, medicated compresses, and herbal steam or wash therapies, 2) clinical diagnosis of gout, 3) suitable study designs, including randomized controlled trials, and 4) study populations defined by the TCM pattern of dampness-heat accumulation.

#### 2.1.5 Data extraction and management

##### 2.1.5.1 Data curation and abstraction process

The literature search results were subjected to standardized curation procedures. Following duplicate removal, the initial screening of titles and abstracts was conducted independently by two investigators (QYL and LS). The same reviewers subsequently obtained and examined the full-text articles for eligibility criteria. Studies meeting the predefined inclusion criteria were processed for data abstraction using EpiData software (EpiData Association, Odense, Denmark).

Using a standardized extraction form, QYL and LS independently retrieved and subsequently cross-verified the following data from the included studies: authorship, publication year, article title, journal name, participant characteristics, sample size, methodological specifications, intervention protocols, treatment duration, outcome parameters and adverse event reports.

#### 2.1.6 Assessment of risk of bias in included studies

##### 2.1.6.1 Methodological quality appraisal

The methodological quality of the included trials was assessed following Cochrane recommendations (14). Publication bias was evaluated using Egger’s test in subgroups containing at least ten studies. Two reviewers (QYL and LS) independently assessed the risk of bias using the Cochrane Risk of Bias Tool 2 (RoB2, 2019 revision) via the official macro Excel file. Disagreements were resolved through discussion or, if necessary, by consulting a third reviewer (GJF). The RoB2 tool evaluates five domains: the randomization process, deviations from intended interventions, missing outcome data, measurement of the outcome, and selective reporting. Each domain was rated as “low risk,” “some concerns,” or “high risk,” and the overall risk of bias for each RCT was determined based on the assessments across these five domains.

#### 2.1.7 Measures of treatment effects

##### 2.1.7.1 Quantification of treatment effects and statistical methods

Quantification of Treatment Effects: for continuous outcome measures, treatment effects are expressed as the mean difference (MD) with the corresponding 95% confidence interval (CI). Dichotomous outcomes were analyzed using relative risk (RR) with 95% CI. All the statistical computations were performed using Stata software (version 13.0). To account for anticipated clinical and methodological variations across studies, DerSimonian and Laird random effects models were employed for all meta-analyses to generate conservative effect estimates. Between-study heterogeneity was quantified via the I² statistic.

##### 2.1.7.2 Assessment and exploration of heterogeneity

Heterogeneity was quantified using the I² statistic; I² > 75% indicated substantial heterogeneity. To explore potential sources, meta-regression and subgroup analyses were performed:

1. Univariable meta-regression: Random-effects meta-regression was conducted separately for each covariate using Stata, including random sequence generation risk (low risk vs. unclear or high risk), baseline uric acid level (<535, 535–590, or >590 μmol/L), comparator drug class, treatment duration (≤7 days, 7–14 days, or ≥14 days), Tufuling dose (<30 g/day, 30–45 g/day, or ≥45 g/day), and combinations of Tufuling with high-frequency Chinese herbal medicines (*Tufuling*–*Huangbo*, *Tufuling*–*Bixie*, or *Tufuling*–*Huangbo*–*Bixie*–*Niuxi*).
2. Multivariable meta-regression: Based on clinical *a priori* hypotheses and univariable findings, variables potentially contributing to heterogeneity were selected to construct a multivariable model. The significance level was set at *P* < 0.05. Following the empirical rule, multivariable analysis was performed only when the total number of included studies was ≥ 10 × (number of covariates + 1) to avoid overfitting.
3. Subgroup analyses: Categorical covariates were stratified by subgroup to visually display between-group differences.

##### 2.1.7.3 Primary prespecified comparisons

The primary prespecified comparisons for this systematic review included the following: (1) Chinese herbal medicine (CHM) versus pharmacotherapy; (2) TCM comprehensive therapy versus pharmacotherapy; (3) CHM combined with pharmacotherapy versus pharmacotherapy alone; and (4) TCM comprehensive therapy plus pharmacotherapy versus pharmacotherapy alone.

##### 2.1.7.4 Sensitivity analysis

To evaluate the robustness of the primary meta-analysis findings, a leave-one-out sensitivity analysis was performed. This method involved iteratively removing each individual included study and recalculated the pooled effect estimate (e.g., mean difference [MD] or risk ratio [RR]) and its 95% confidence interval (CI) for all primary and secondary outcomes across every comparison (i.e., CHM monotherapy vs. WM, CHM comprehensive therapy vs. WM, CHM+WM vs. WM, CHM comprehensive therapy+WM vs. WM). The stability of the results was assessed by examining the fluctuation range of the recalculated point estimates and the consistency in statistical significance (determined by whether the 95% CI included the null value: 0 for MDs, 1 for RRs). The influence of each omitted study on the overall heterogeneity (I² statistic) was also observed. The findings were interpreted as follows:

- Highly Robust: Minimal variation in the point estimate across all iterations, with the recalculated the 95% CIs, consistently excluding the null value.
- Moderately Robust: Moderate variation in the point estimate, but the direction of effect and statistical significance remained unchanged in most iterations.
- Less Robust/Low Robustness: Substantial variation in the point estimate upon removal of specific studies and/or a change in the statistical conclusion (i.e., the CI crossing the null value in one or more iterations). For outcomes with high baseline heterogeneity, particular attention was paid to whether the exclusion of any study led to a marked reduction in the I² value.

This analysis aimed to identify whether the overall conclusions were disproportionately dependent on any single study, and to provide a measure of confidence in the meta-analytic results.

##### 2.1.7.5 Safety analysis

Adverse events (AEs) were independently extracted by two reviewers (QYL and LS). Using the Common Terminology Criteria for Adverse Events (CTCAE) version 5.0, AEs were graded as mild or moderate; no severe AEs were documented in any included study. Studies were stratified into three categories according to AE reporting status: explicit AE counts, zero-event studies (included in the meta-analysis with a continuity correction of 0.5), and missing data (excluded from quantitative synthesis). Relative risks (RRs) and 95% confidence intervals (CIs) were estimated using the Mantel-Haenszel random-effects model, with the Peto one-step method applied as a sensitivity analysis. zero-event studies were incorporated with a continuity correction of 0.5. Risk differences (RDs) and numbers needed to treat (NNTs) were calculated to quantify absolute effects. The certainty of evidence was evaluated using the GRADE framework.

##### 2.1.7.6 Assessment of evidence quality

The overall quality of evidence for critical outcomes was evaluated via the Grading of Recommendations Assessment, Development and Evaluation (GRADE) framework.

### 2.2 Materials and methods for network pharmacology

#### 2.2.1 Database resources

The following databases were utilized for data acquisition and analysis in this study (**Table 1**):

**Table 1:** Databases used in this study.

Database name	Description	Website
TCMSP	Traditional Chinese Medicine Systems Pharmacology Database and Analysis Platform,TCMSP database	https://tcmsp-e.com/tcmsp.php
STRING	Search Tool for the Retrieval of Interacting Genes/Proteins, STRING database	https://string-db.org/
NCBI	National Center for Biotechnology Information, NCBI database	https://www.ncbi.nlm.nih.gov/
PDB	Protein Data Bank, PDB Database	https://www.rcsb.org/
OmicShare	OmicShare website	https://www.omicshare.com/tools/
PubChem	Public Chemical Database,Pubchem database	https://pubchem.ncbi.nlm.nih.gov/
SwissTargetPrediction	Swiss Target Prediction, Swiss database	http://www.swisstargetprediction.ch
GeneCards	GeneCards: The Human Gene Database,GeneCards database	https://www.genecards.org/
DrugBank	DrugBank: A Comprehensive Database for Drug Discovery and Exploration,DrugBank database	https://go.drugbank.com/
TTD	Therapeutic Target Database, TTD Database	https://db.idrblab.net/ttd/

#### 2.2.2 Software tools

The following software packages and their respective versions were employed for data processing, network analysis, and visualization:

- Cytoscape (v3.10.3) with the Cytoscape plugin (CytoHubba) plugin for network construction and centrality analyses.
- Chem3D (v23.1.1) was used for the molecular structure conversion and minimization.
- PyMOL (v2.3.0) was used for protein structure preparation and visualization of the molecular docking results.
- AutoDock Vina (v1.1.2) was used for the molecular docking simulations.

#### 2.2.3 Methodology

##### 2.2.3.1 Acquisition of active compounds for Tufuling, Huangbo, Bixie and target prediction

The chemical constituents of *Tufuling, Huangbo, and Bixie* were obtained from the Traditional Chinese Medicine Systems Pharmacology Database and Analysis Platform (TCMSP). To identify potential bioactive compounds, a screening process was applied using specific pharmacokinetic parameters: oral bioavailability (OB) of 30% or more, drug likeness (DL) of at least 0.18, and blood-brain barrier (BBB) permeability exceeding -0.3. The canonical SMILES of these active ingredients were sourced from the PubChem database and analyzed using the SwissTargetPrediction platform to predict their likely protein targets. The resulting targets were compiled, and duplicates were removed.

##### 2.2.3.2 Identification of therapeutic targets for gout

Disease-related targets for gout and gouty arthritis (GA) were gathered from DisGeNET, GeneCards, and the Therapeutic Target Database (TTD). The TTD targets have support from clinical evidence, patents, and literature. We merged targets from these databases and removed duplicates to create a complete list of therapeutic targets for gout.

##### 2.2.3.3 Identification of overlapping targets of Tufuling, Tufuling-Huangbo, and Bixie against gout.

The potential therapeutic targets of *Tufuling*, *Tufuling*-*Huangbo*, and *Bixie* in gout were identified by determining the intersection between the predicted targets of the active compounds of *Tufuling*, *Huangbo*, and *Bxie* and the known therapeutic targets of gout. A Venn diagram was generated using the OmicShare online tool to visualize the overlapping genes, which are considered potential key targets for the treatment of gout.

##### 2.2.3.4 Construction of three independent “Compound-Target-Disease” network

To visualize the multicomponent, multitarget characteristics of each intervention, three separate networks were constructed in Cytoscape (v3.10.3): *Tufuling* alone, the *Tufuling*–*Huangbo* pair, and *Bixie* alone. Each network incorporated the respective active compounds, their intersecting targets with gout, and the disease node. Topological analysis was conducted for each network, and nodes were ranked by degree centrality to identify key compounds and targets.

##### 2.2.3.5 Construction and analysis of the Protein–Protein Interaction (PPI) Network

The overlapping targets were imported into the STRING database (https://string-db.org/) to build a PPI network. We set the minimum interaction score to over 0.40. Next, we imported the network into Cytoscape and analyzed it with the CytoHubba plugin. We calculated topological parameters like betweenness centrality (BC), closeness centrality (CC), degree centrality (DC), eigenvector centrality (EC), and local average connectivity (LAC). Then, we filtered nodes based on these parameters to find the core targets in the PPI network.

##### 2.2.3.6 Gene ontology and Kyoto encyclopedia of genes and genomes enrichment analyses

Overlapping target genes were converted to Ensembl IDs using the NCBI database. Functional enrichment analysis of GO terms (biological process, cellular component, and molecular function) and KEGG pathways was conducted using the OmicShare platform. The species was specified as Homo sapiens (Genome assembly: GRCh38.p12/GRCh38.p13). Bar charts and bubble plots were generated to visualize significantly enriched terms and pathways.

##### 2.2.3.7 Molecular docking for binding affinity prediction and hypothesis generation

###### 2.2.3.7.1 Ligand preparation

The three-dimensional structures of the core active compounds (ligands) were downloaded from the PubChem database in the SDF format. These structures were subsequently energy-minimized and converted to the Mol2 format using Chem3D software.

###### 2.2.3.7.2 Receptor preparation

The crystal structures of the core target proteins (receptors) were retrieved from the Protein Data Bank (PDB), prioritizing structures of human origin. The PyMOL software (v2.3.0) was used to remove the bound water molecules, native ligands, and cofactors from the protein structures. The purified protein structures were saved in the PDB format.

###### 2.2.3.7.3 File format conversion

Both the ligand (Mol2) and receptor (PDB) files were converted to the PDBQT format, which includes atomic coordinates and partial charges, via AutoDock Vina (v1.1.2). The active site of the protein receptor was defined in the docking simulation.

###### 2.2.3.7.4 Docking execution and analysis

Molecular docking was performed using AutoDock Vina (v1.1.2) to predict binding affinities and generate structural hypotheses regarding ligand–receptor interactions. This is shown as binding energy in kcal/mol between active compounds and target proteins. Usually, the smaller the value, the stronger the affinity. We selected ligand-receptor complexes with the lowest binding energies for further analysis. PyMOL (v2.3.0) helped visualize the docking poses and analyze specific binding interactions. This includes hydrogen bonds and hydrophobic contacts at the binding site.

## 3 Results

### 3.1 Modern literature results

#### 3.1.1 Description of included studies

##### 3.1.1.1 Search yield and study selection

**Figure 1:**
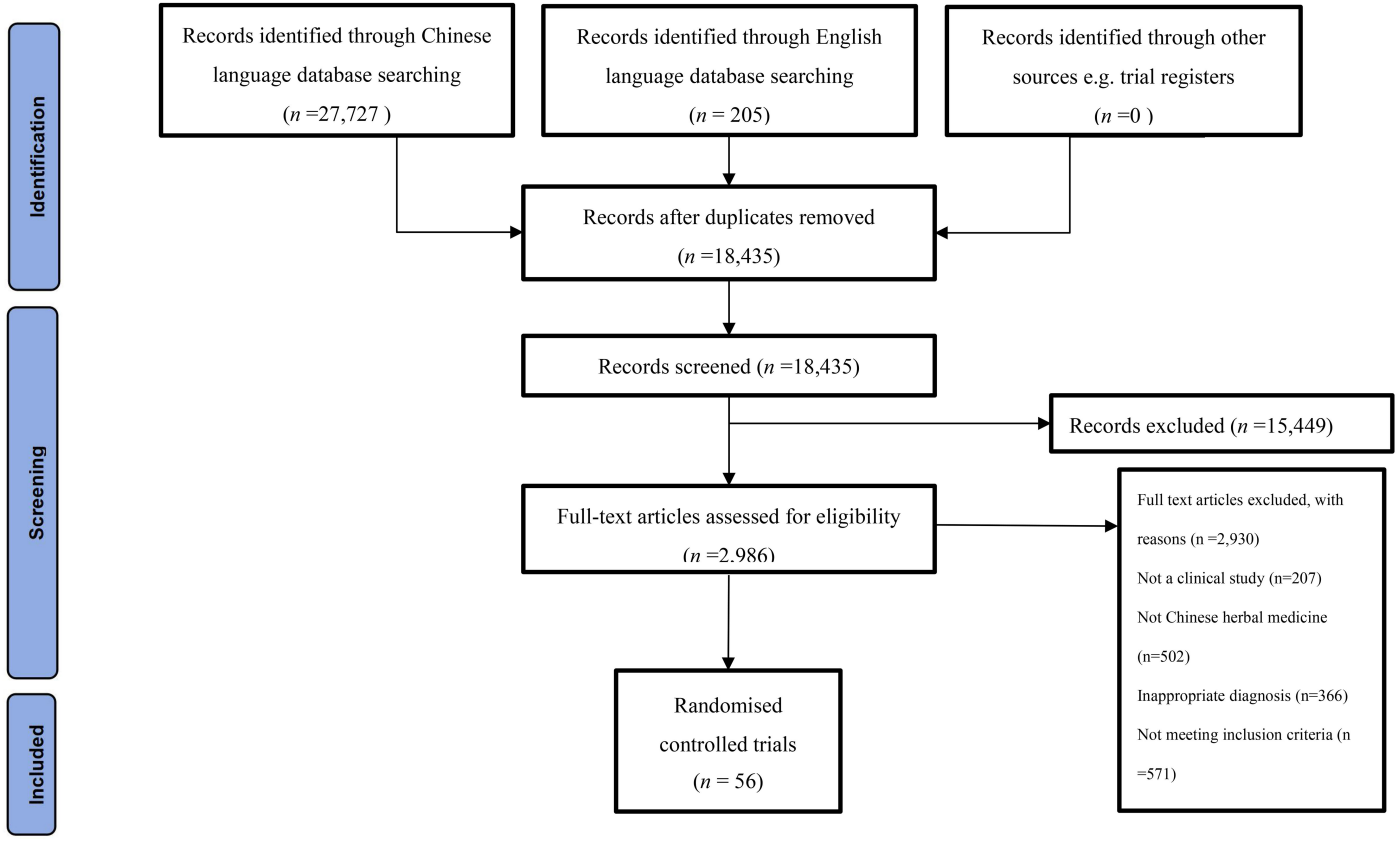
Flow chart of study selection process.

The systematic search found 27,932 records from the targeted databases. After screening, as shown in **Figure 1**, we included fifty-six randomized controlled trials (RCTs) with 4,605 participants in the systematic review (14-69).

##### 3.1.1.2 Trial and participant characteristics

All included studies were randomized, parallel-group, controlled trials conducted exclusively in China and reported in Chinese between 2008 and 2023. Participant diagnosis was consistently based on the established gout classification criteria, primarily the 1977 American College of Rheumatology (ACR) criteria or the 2015 ACR/European Alliance of Associations for Rheumatology (EULAR) criteria.

The enrolled population had a mean or median age ranging from 18 to 80 years, with a reported duration of gout from one week to four years. The intervention period was ≤14 days in all studies. A follow-up assessment was documented in only four trials, extending up to three months (reference 65). The key characteristics of the included studies are listed in **Table 2**.

**Table 2:** The key characteristics of the included studies.

NO.	Study	Sample size	Sample size	Mean age (y)	Mean age (y)	Treatment	Control	Duration (d)	VAS	Effective rate	UA	CRP	ESR	WBC	TCM Syndrome Scoring	AEs
		(I)	(C)	(I)	(C)											
1	Cao MZ 2020 (15)	44	44	53.56	53.46	Simiao Formula&NSAIDs	NSAIDs	7	✓	✓	✓	✓	✓	×	✓	×
2	Wan L 2018 (16)	54	52	46.35	45.52	Self-made Formula&NSAIDs	NSAIDs	14	✓	✓	✓	✓	✓	×	×	✓
3	Du YY 2018 (17)	38	37	50.29	51.22	Self-made Formula&NSAIDs	NSAIDs	7	✓	✓	✓	✓	✓	×	✓	✓
4	Huang QM 2013 (18)	35	34	43.94	48.94	Self-made Formula&NSAIDs	NSAIDs	7	×	✓	✓	×	×	✓	✓	✓
5	Jiang D 2023 (19)	50	50	39.21	38.97	self-made Formula&External use Furong Plaster	NSAIDs	5	✓	✓	×	×	×	×	×	✓
6	Li C 2022 (20)	38	38	43.82	44.89	Self-made Formula&NSAIDs	NSAIDs	7	×	✓	✓	✓	✓	×	✓	✓
7	Li D 2023 (21)	60	60	18∼75	21∼75	Self-made Formula&NSAIDs	NSAIDs	14	✓	✓	✓	✓	✓	×	×	✓
8	Li L 2021 (22)	68	68	68.47	68.85	Self-made Formula(p.o.&External use)&Colchicine	Colchicine	14	✓	✓	×	×	✓	×	×	✓
9	Li SG 2020 (23)	47	47	47.2	46.7	Self-made Formula&NSAIDs&Colchicine	NSAIDs&Colchicine	7	×	✓	✓	✓	✓	×	×	✓
10	Lin HH 2023 (24)	25	25	50.12	48.08	Self-made Formula&NSAIDs	NSAIDs	14	×	✓	✓	✓	✓	×	×	×
11	Liu F 2022 (25)	40	40	45.62	45.11	Self-made Formula&Meridian segment alignment method&Febuxostat	Febuxostat	7	×	✓	✓	✓	✓	×	×	✓
12	Liu MY 2016 (26)	30	30	52.27	55.3	Self-made Formula&NSAIDs&Colchicine	NSAIDs&Colchicine	7	×	✓	✓	✓	✓	×	✓	×
13	Liu SC 2021 (27)	40	40	33.35	34.75	Simiao Formula&Simiaoyongan Formula&ACU	NSAIDs	12	×	✓	✓	×	✓	✓	×	✓
14	Liu T 2019 (28)	29	30	15∼57	20∼55	Self-made Formula&NSAIDs	NSAIDs	7	✓	✓	✓	✓	✓	×	✓	✓
15	Liu YX 2008 (29)	40	40	40.27	41.85	Simiao Formula&External use self-made Formula	NSAIDs	7	×	✓	✓	✓	✓	×	×	✓
16	Lyu FL 2022 (30)	30	30	47.27	45.23	Self-made Formula&NSAIDs	NSAIDs	7	✓	×	✓	✓	✓	×	✓	✓
17	Ma YN 2020 (31)	25	25	18∼80	18∼80	Self-made Formula&NSAIDs	NSAIDs	14	✓	✓	✓	✓	✓	×	×	×
18	Nan YT 2020 (32)	25	25	43.48	41.2	Self-made Formula&NSAIDs	NSAIDs	7	✓	✓	✓	✓	✓	✓	✓	✓
19	Nie JP 2012 (33)	45	45	28∼80	25∼76	self-made Formula	NSAIDs	7	×	✓	×	✓	✓	×	×	✓
20	Ouyang HX 2020 (34)	44	43	52.34	48.51	Self-made Formula(p.o.&External use)&NSAIDs	NSAIDs	10	×	✓	×	×	✓	×	✓	✓
21	Qi ZM 2016 (35)	105	105	38∼69	36∼59	self-made Formula	Colchicine	14	×	✓	✓	✓	✓	×	×	✓
22	Qi ZM 2017 (36)	60	60	35∼67	33∼68	self-made Formula	Colchicine	14	×	✓	✓	✓	×	×	×	✓
23	Qin GF 2019 (37)	78	78	20∼65	21∼64岁	Self-made Formula(p.o.&External use)&NSAIDs	NSAIDs	7	✓	✓	✓	✓	✓	×	×	✓
24	Rong SQ 2021 (38)	40	40	48.51	47.23	Self-made Formula&NSAIDs	NSAIDs	14	×	✓	✓	✓	✓	×	×	×
25	Shen N 2023 (39)	31	30	36∼68	33∼69	Self-made Formula(p.o.&External use)&NSAIDs&Colchicine	NSAIDs&Colchicine	7	✓	✓	✓	✓	✓	×	×	✓
26	Song JC 2021 (40)	36	35	35∼67	36∼68	Self-made Formula&NSAIDs	NSAIDs	15	×	✓	✓	✓	✓	✓	×	✓
27	Song JF 2017 (41)	75	75	30∼65	28∼65	Self-made Formula&NSAIDs	NSAIDs	14	✓	✓	✓	✓	✓	×	×	×
28	Sun JW 2017 (42)	55	53	40.11	38.52	Self-made Formula&NSAIDs	NSAIDs	14	×	✓	✓	✓	✓	×	×	✓
29	Tong Y 2016 (43)	20	20	43.5	45.8	self-made Formula	Colchicine	10	×	✓	✓	×	×	✓	×	✓
30	Wang GS 2020 (44)	40	40	31∼65	28∼62	self-made Formula&ACU	Colchicine	14	×	✓	✓	×	×	×	×	×
31	Wang HL 2016 (45)	60	30	20∼66	21∼64	Self-made Formula&NSAIDs	NSAIDs	14	✓	✓	✓	✓	✓	×	×	✓
32	Wang L 2011 (46)	29	29	28∼65	25∼63	self-made Formula	NSAIDs	7	✓	✓	✓	✓	×	×	✓	✓
33	Wang YX 2022 (47)	40	40	47.58	46.75	Self-made Formula&NSAIDs	NSAIDs	7	✓	✓	✓	✓	✓	×	✓	✓
34	Wang YY 2020 (48)	32	32	20∼72	18∼73	Self-made Formula&NSAIDs	NSAIDs	7	✓	✓	✓	✓	✓	×	×	✓
35	Wei YL 2020 (49)	34	33	43.74	44.91	Self-made Formula&NSAIDs	NSAIDs	14	✓	✓	✓	✓	✓	×	✓	✓
36	Wu YY 2020 (50)	40	40	41∼65	39∼61	Self-made Formula&NSAIDs&Allopurinol	NSAIDs&Allopurinol	7	✓	✓	✓	×	×	×	×	✓
37	Xi YJ 2021 (51)	46	46	20∼79	19∼80	Self-made Formula&NSAIDs	NSAIDs	14	✓	✓	✓	✓	✓	✓	✓	✓
38	Xia SJ 2023 (52)	53	51	56.74	55.45	Self-made Formula&NSAIDs&Colchicine	NSAIDs&Colchicine	7	×	✓	✓	✓	✓	×	✓	✓
39	Xiao GR 2017 (53)	32	32	48	47	External use self-made Formula&ACU	NSAIDs	14	×	✓	✓	×	×	×	×	×
40	Xie HF 2013 (54)	30	30	44.25	45.63	self-made Formula(p.o.&External use)	NSAIDs&Colchicine	10	×	✓	✓	×	✓	×	×	✓
41	Xie HZ 2017 (55)	45	45	40.53	39.3	self-made Formula	Allopurinol&Probenecid	14	×	✓	✓	✓	✓	×	×	✓
42	Xing ZL 2018 (56)	43	43	25∼67	27∼65	self-made Formula(p.o.&External use)	NSAIDs	7	×	✓	✓	✓	✓	×	×	×
43	Xiong YM 2021 (57)	39	39	33∼64	31∼62	Self-made Formula&NSAIDs	NSAIDs	14	×	✓	✓	✓	✓	×	×	×
44	Xu DD 2019 (58)	43	42	37∼65	35∼64	Self-made Formula&NSAIDs&Colchicine&Allopurinol	NSAIDs&Colchicine&Allopurinol	14	×	✓	✓	✓	✓	×	×	×
45	Xu FQ 2020 (59)	30	30	NS	NS	Self-made Formula&NSAIDs&Colchicine	NSAIDs&Colchicine	7	×	✓	×	✓	✓	×	×	✓
46	Xu RM 2020 (60)	30	30	50.33	45.47	Self-made Formula&NSAIDs	NSAIDs	14	✓	✓	✓	✓	✓	×	✓	✓
47	Yang C 2019 (61)	45	47	36∼59	34∼58	Self-made Formula&Colchicine&Benzbromarone	Colchicine&Benzbromarone	14	×	✓	✓	✓	✓	×	×	✓
48	Yang D 2014 (62)	33	32	48	48.25	Self-made Formula&NSAIDs	NSAIDs	7	×	✓	✓	✓	✓	×	×	✓
49	Yang L 2021 (63)	40	40	23∼65	25∼65	Self-made Formula&NSAIDs&Colchicine	NSAIDs&Colchicine	7	×	✓	✓	✓	✓	×	×	✓
50	Yu JY 2022 (64)	45	45	35∼72	35∼71	Self-made Formula&NSAIDs	NSAIDs	14	×	✓	✓	×	×	×	✓	×
51	Yuan B 2014 (34)	25	25	32∼49	35∼52	self-made Formula	Colchicine	14	✓	✓	✓	✓	✓	×	×	✓
52	Zeng JY 2021 (65)	51	49	52.44	52.34	Self-made Formula&NSAIDs&Colchicine	NSAIDs&Colchicine	14	×	×	✓	✓	×	×	×	×
53	Zhang JH 2018 (66)	30	30	22∼69	20∼70	self-made Formula(p.o.&External use)	NSAIDs	14	✓	✓	✓	✓	✓	×	×	✓
54	Zhang M 2021 (67)	30	30	38∼72	39∼72	Self-made Formula(p.o.&External use)&NSAIDs	NSAIDs	7	✓	✓	✓	✓	✓	×	✓	✓
55	Zhou LT 2023 (68)	22	22	25∼69	26∼65	Self-made Formula&NSAIDs&Benzbromarone	NSAIDs&Benzbromarone	14	×	✓	✓	✓	✓	×	✓	✓
56	Zou WC 2022 (69)	30	30	51.4	50	Self-made Formula&NSAIDs	NSAIDs	7	✓	✓	✓	✓	✓	✓	✓	×

C, control; I, intervention; F, female; M, male; D, day/days; y, year/years; p.o., Oral administration / Per os; NSAIDs, Non-Steroidal Anti-Inflammatory Drug; ACU, Acupuncture; VAS, Visual analogue scale; UA, Uric acid; CRP, C-reactive protein; ESR, Erythrocyte sedimentation rate; WBC, White blood cell count; AEs, Adverse Events.

##### 3.1.1.3 Literature identification and study categorization

The systematic search identified fifty-six randomized controlled trials (RCTs) that evaluated various traditional Chinese medicine (TCM) interventions for gout management. These interventions were categorized as Chinese herbal medicine (CHM) monotherapy, TCM comprehensive therapy, or integrated Chinese–Western medicine regimens.

The distribution of studies across the intervention categories was as follows:

- Seven trials investigated CHM alone (references 14–20).
- Eight studies evaluated TCM comprehensive therapy (References 21–28).
- Thirty-four trials assessed integrated Chinese–Western medicine approaches (reference 29–62).
- Seven studies directly compared TCM comprehensive therapy with integrated Chinese–Western medicine (References 63–69).

##### 3.1.1.4 Herbal constituents of prescriptions

The analyzed formulae included 196 distinct medicinal herbs. *Tufuling*土茯苓 was the core botanical drug, most frequently combined with the following herbs: *Huangbo*黄柏 (48 studies), *Bixie*萆薢 (41 studies), *Niuxi*牛膝 (39 studies), *Yiyiren*薏苡仁 (36 studies), *Cangzhu*苍术 (33 studies), *Zexie*泽泻 (23 studies), *Weilingxia*n威灵仙 (21 studies), *Rendongteng*忍冬藤 (19 studies), and *Guizhi*桂枝 (8 studies).

##### 3.1.1.5 Control interventions

The control groups received pharmacotherapy and lifestyle modification strategies. The pharmacological comparators included nonsteroidal anti-inflammatory drugs (NSAIDs), colchicine, allopurinol, and benzbromarone (Non-pharmacological management involves patient education and supportive care, emphasizing a low-purine diet, consumption of dairy products and fresh vegetables, sufficient hydration, joint immobilization during acute attacks, and psychosocial support.

#### 3.1.2 Risk of bias in the included studies

Methodological quality assessment of the included studies

**Figure 2:**
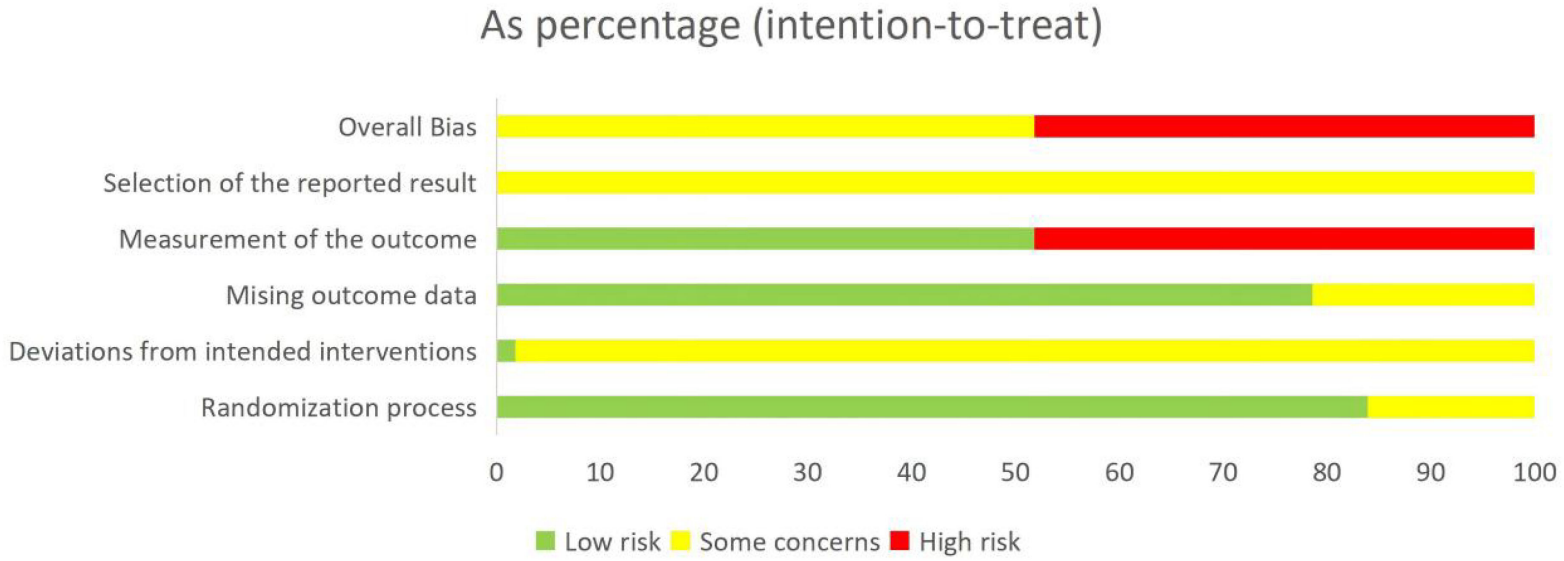
Risk of bias graph of the included studies.

**Figure 3:**
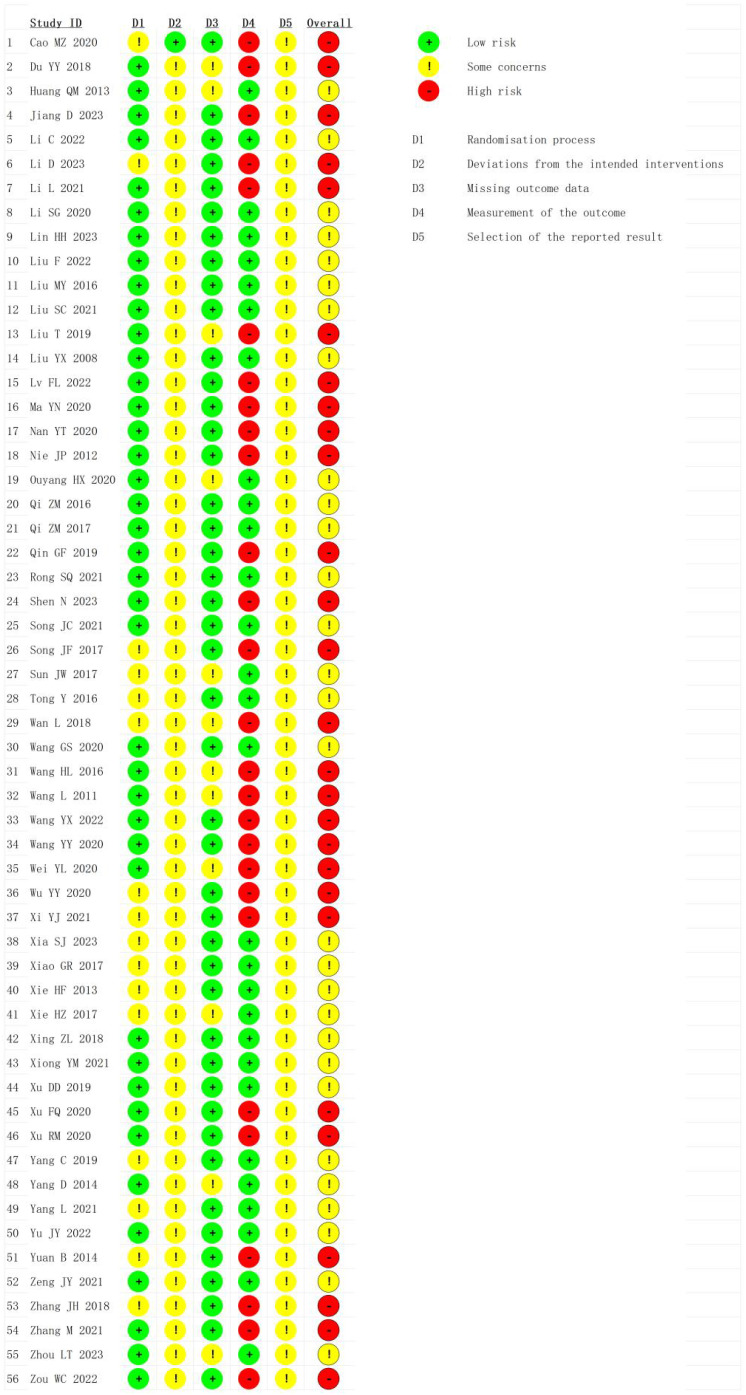
Risk of bias summary.

The risk of bias for the 56 randomized trials is shown in **Figures 2** and **3**. Fifty-six RCTs were evaluated using the Cochrane Risk of Bias 2.0 tool. Overall, 48.2% (27/56) were rated as high risk, 51.8% (29/56) as some concerns, and none as low risk.

Among the five domains, randomization (83.9% low risk) and missing outcome data (78.6% low risk) were performed relatively well performed. Deviations from intended interventions were rated as some concerns in 98.2% of studies due to open-label design. The measurement of the outcome domain showed a polarized pattern: all 29 studies using objective laboratory indicators (uric acid) were rated as low risk, whereas all 27 studies using subjective outcomes (VAS pain score, effective rate) were rated as high risk due to lack of blinding. For selective reporting, 51.8% were rated as some concerns and 48.2% as high risk, attributable to the absence of trial registration.

A key finding was that the nature of outcome indicators directly determined the overall risk of bias. Studies with objective outcomes raised some concerns despite the inability to implement blinding, whereas studies with subjective outcomes inevitably resulted in high risk due to unblinded assessment. The principal methodological deficiencies included absence of trial registration (100%), difficulty in blinding implementation (98.2%), and unblinded subjective outcome assessment (48.2%).

No significant publication bias was detected using Egger’s test (t=1.86, *P* = 0.07). The overall methodological quality of the evidence was moderate.

#### 3.1.3 Efficacy

##### 3.1.3.1 CHM versus Western medicine treatment

###### 3.1.3.1.1 Visual analog scale

**Figure 4:**
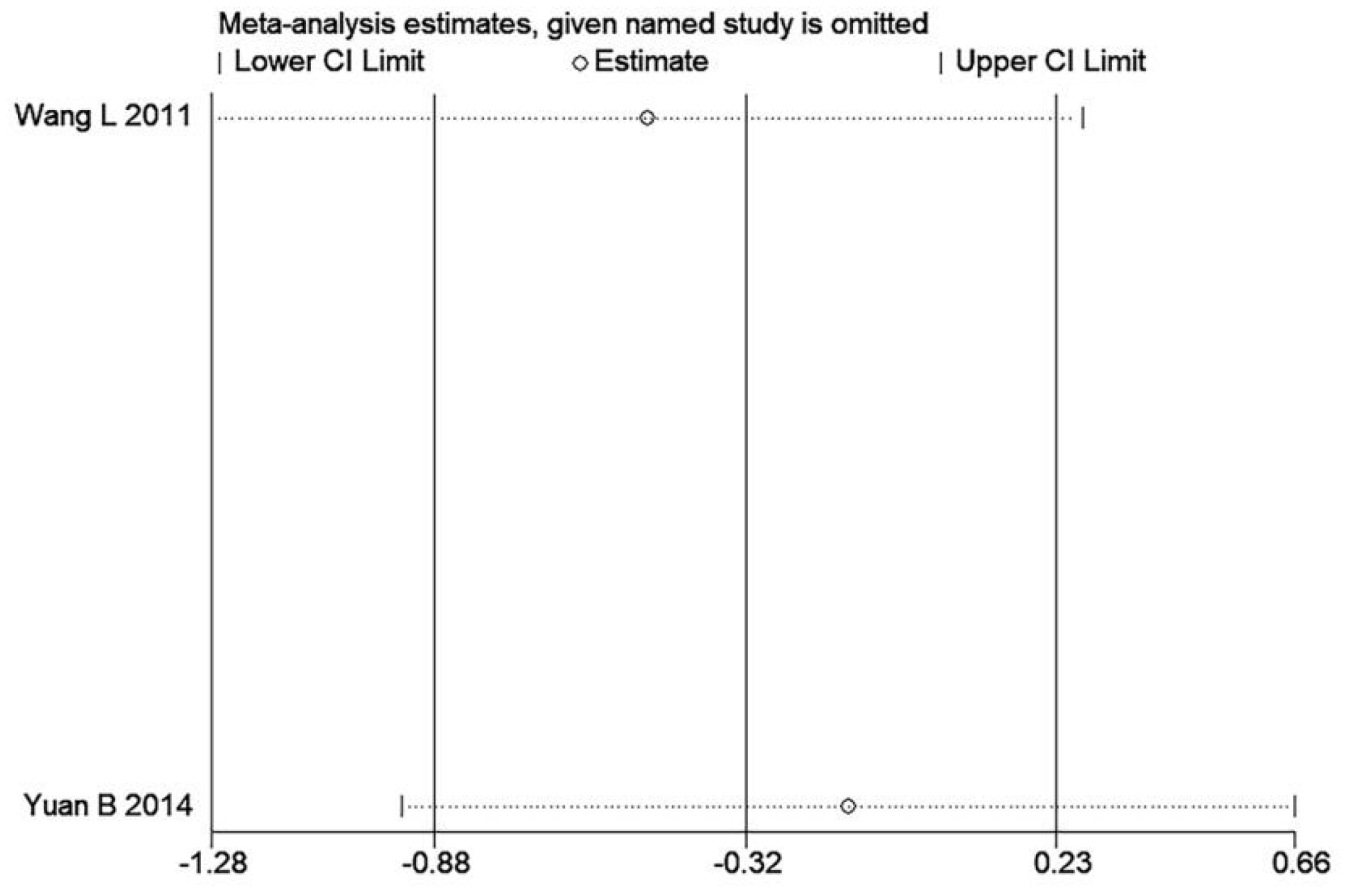
Results of the VAS sensitivity analysis in the CHM vs. WM group.

Two studies (34, 46) involving 108 patients compared the efficacy of Chinese herbal medicine (CHM) alone with that of conventional Western medicine (WM) in improving VAS. The results indicated that CHM alone was not superior to conventional Western medicine [MD -0.33 (-0.88, 0.23), I^2^ = 0.00%]. Prespecified subgroup analyses, including risk of bias subgroups, comparator drug classes, baseline uric acid levels, treatment duration, dose of *Tufuling* and combinations of *Tufuling* with high-frequency CHM failed to demonstrate a significant benefit for CHM (**Table 3**). Sensitivity analysis revealed that the MD ranged from -1.28 to 0.66, with the confidence interval frequently including zero, indicating the limited robustness of this finding (**Figure 4**).

**Table 3:** Results of meta-analysis (total and subgroup).

Treatment vs. comparison	Outcomes (unit)	Group	Subgroup	No. of ctudies	MD/RR [95% CI]	I^2^
**CHM vs. Western medicine treatment**	Visual analogue scale (VAS)	All studies	All studies	2(108)	-0.33[-0.88, 0.23]	0.0%
		Risk of bias SG	Low risk of bias SG	2(108)	-0.33[-0.88, 0.23]	0.0%
		Comparator Drug class	NSAIDs	1(58)	-0.14[-0.94, 0.66]	0.0%
			Colchicine	1(50)	-0.50[-1.28, 0.28]	0.0%
		UA level at baseline	<535μmol/L	1(58)	-0.14[-0.94, 0.66]	0.0%
			535-590μmol/L	1(50)	-0.50[-1.28, 0.28]	0.0%
		Treatment duration	≤7days	1(58)	-0.14[-0.94, 0.66]	NA
			7-14days	1(50)	-0.50[-1.28, 0.28]	NA
		Dose of Tufuling	<30g/day	2(108)	-0.33[-0.88, 0.23]	0.0%
		Combinations of Tufuling with high-frequency CHM	Tufuling-Huangbo	2(108)	-0.33[-0.88, 0.23]	0.0%
			Tufuling-Bixie	2(108)	-0.33[-0.88, 0.23]	0.0%
			Tufuling-Huangbo-Bixie-Niuxi	1(50)	-0.50[-1.28, 0.28]	NA
	Effective rate	All studies	All studies	7(658)	1.06[1.01, 1.11]*	0.0%
		Risk of bias SG	Low risk of bias SG	6(568)	1.06[1.00, 1.12]	0.0%
		Comparator Drug class	NSAIDs	2(148)	0.97[0.88, 1.07]	0.0%
			Colchicine	4(420)	1.10[1.03, 1.17]*	0.0%
			Allopurinol&Probenec	1(90)	1.03[0.88, 1.20]	NA
		UA level at baseline	<535μmol/L	4(478)	1.07[1.00, 1.15]	20.1%
			535-590μmol/L	2(90)	1.05[0.91, 1.22]	0.0%
		Treatment duration	≤7days	2(148)	0.97[0.88, 1.07]	0.0%
			7-14days	1(40)	1.06[0.84, 1.34]	NA
			≥14 days	4(470)	1.09[1.03, 1.15]*	0.0%
		Dose of Tufuling	<30g/day	1(40)	1.05[1.00, 1.11]	0.0%
			30-45g/day	6(618)	1.06[0.84, 1.34]	NA
		Combinations of Tufuling with high-frequency CHM	Tufuling-Huangbo	6(568)	1.06[1.00, 1.12]	12.3%
			Tufuling-Bixie	6(568)	1.06[1.00, 1.12]	12.3%
			Tufuling-Huangbo-Bixie-Niuxi	2(90)	1.05[0.91, 1.22]	0.0%
	Uric acid (μmol/L)	All studies	All studies	6(568)	-47.56[-68.92, -26.21]*	81.1%
		Risk of bias SG	Low risk of bias SG	5(478)	-42.11[-68.38, -15.84]*	80.7%
		Comparator Drug class	NSAIDs	1(58)	8.90[-44.00, 61.80]	NA
			Colchicine	4(420)	-49.55[-76.10, -23.00]*	81.9%
			Allopurinol&Probenec	1(90)	-66.93[-81.16, -52.70]*	NA
		UA level at baseline	<535μmol/L	4(478)	-61.90[-82.65, -41.14]*	64.6%
			535-590μmol/L	2(90)	-28.76[-43.00, -14.52]*	0.0%
		Treatment duration	≤7days	1(58)	8.90[-44.00, 61.80]	0.0
			7-14days	1(40)	-32.00[-48.57, -15.43]*	0.0
			≥14 days	4(470)	-59.78[-80.82, -38.75]*	73.1%
		Dose of Tufuling	<30g/day	1(40)	-32.00[-48.57, -15.43]*	NA
			30-45g/day	5(510)	-51.41[-75.11, -27.70]*	77.8%
		Combinations of Tufuling with high-frequency CHM	Tufuling-Huangbo	5(478)	-42.11[-68.38, -15.84]*	80.7%
			Tufuling-Bixie	5(478)	-42.11[-68.38, -15.84]*	80.7%
			Tufuling-Huangbo-Bixie-Niuxi	2(90)	-28.76[-43.00, -14.52]*	0.0%
	C-reactive protein (mg/L)	All studies	All studies	6(618)	-2.75[-4.21, -1.29]*	76.6%
		Risk of bias SG	Low risk of bias SG	5(528)	-2.45[-4.16, -0.75]*	80.6%
		Comparator Drug class	NSAIDs	2(148)	-2.48[-12.14, 7.19]	71.8%
			Colchicine	3(380)	-3.43[-3.99, -2.87]*	0.0%
			Allopurinol&Probenec	1(90)	-4.14[-6.33, -1.95]*	NA
		UA level at baseline	<535μmol/L	4(478)	-3.50[-4.05, -2.95]*	0.0%
			535-590μmol/L	1(50)	-3.05[-6.58, 0.48]	NA
		Treatment duration	≤7days	2(148)	-2.48[-12.14, 7.19]	71.8%
			7-14days	4(470)	-3.47[-4.02, -2.93]*	0.0%
			30-45g/day	6(618)	-2.75[-4.21, -1.29]*	76.6%
		Combinations of Tufuling with high-frequency CHM	Tufuling-Huangbo	5(528)	-2.45[-4.16, -0.75]*	80.6%
			Tufuling-Bixie	5(528)	-2.45[-4.16, -0.75]*	80.6%
			Tufuling-Huangbo-Bixie-Niuxi	1(50)	-3.05[-6.58, 0.48]	NA
	Erythrocyte sedimentation rate (mm/h)	All studies	All studies	4(440)	-5.76[-11.02, -0.50]*	91.9%
		Risk of bias SG	Low risk of bias SG	3(350)	-4.39[-10.34, 1.55]	93.0%
		Comparator Drug class	NSAIDs	1(90)	1.80[-0.54, 4.14]	NA
			Colchicine	2(260)	-8.03[-16.01, -0.05]*	82.3%
			Allopurinol&Probenec	1(90)	-10.00[-14.51, -5.49]*	NA
		UA level at baseline	<535μmol/L	2(300)	-6.85[-12.11, -1.59]*	80.0%
			535-590μmol/L	1(50)	-12.81[-19.44, -6.18]*	NA
		Treatment duration	≤7days	1(90)	1.80[-0.54, 4.14]	NA
			≥14 days	3(350)	-8.44[-13.64, -3.24]*	79.8%
			30-45g/day	4(440)	-5.76[-11.02, -0.50]*	91.9%
		Combinations of Tufuling with high-frequency CHM	Tufuling-Huangbo	3(350)	-4.40[-10.34, 1.55]	93.0%
			Tufuling-Bixie	3(350)	-4.40[-10.34, 1.55]	93.0%
			Tufuling-Huangbo-Bixie-Niuxi	1(50)	-12.81[-19.44, -6.18]*	NA
	White blood cell count (×10^9^/L)	All studies	All studies	1(40)	-2.00[-3.21, -0.79]*	NA
		Risk of bias SG	Low risk of bias SG	1(40)	-2.00[-3.21, -0.79]*	NA
		Comparator Drug class	Colchicine	1(40)	-2.00[-3.21, -0.79]*	NA
		UA level at baseline	535-590μmol/L	1(40)	-2.00[-3.21, -0.79]*	NA
		Treatment duration	7-14days	1(40)	-2.00[-3.21, -0.79]*	NA
		Dose of Tufuling	<30g/day	1(40)	-2.00[-3.21, -0.79]*	NA
		Combinations of Tufuling with high-frequency CHM	Tufuling-Huangbo	1(40)	-2.00[-3.21, -0.79]*	NA
			Tufuling-Bixie	1(40)	-2.00[-3.21, -0.79]*	NA
			Tufuling-Huangbo-Bixie-Niuxi	1(40)	-2.00[-3.21, -0.79]*	NA
	TCM Syndrome Scoring	All studies	All studies	1(58)	0.00[-2.20, 2.20]	NA
		Risk of bias SG	Low risk of bias SG	1(58)	0.00[-2.20, 2.20]	NA
		Comparator Drug class	NSAIDs	1(58)	0.00[-2.20, 2.20]	NA
		UA level at baseline	<535μmol/L	1(58)	0.00[-2.20, 2.20]	NA
		Treatment duration	≤7days	1(58)	0.00[-2.20, 2.20]	NA
		Dose of Tufuling	30-45g/day	1(58)	0.00[-2.20, 2.20]	NA
		Combinations of Tufuling with high-frequency CHM	Tufuling-Huangbo	1(58)	0.00[-2.20, 2.20]	NA
			Tufuling-Bixie	1(58)	0.00[-2.20, 2.20]	NA
**CHM comprehensive therapy vs. Western medicine treatment**	Visual analogue scale (VAS)	All studies	All studies	2(160)	-0.66[-0.83, -0.48]*	0.0%
		Risk of bias SG	Low risk of bias SG	2(160)	-0.66[-0.83, -0.48]*	0.0%
		Comparator Drug class	NSAIDs	2(160)	-0.66[-0.83, -0.48]*	0.0%
		UA level at baseline	535-590μmol/L	1(60)	-0.60[-0.93, -0.27]*	NA
		Treatment duration	≤7days	1(100)	-0.68[-0.88, -0.48]*	NA
			≥14 days	1(60)	-0.60[-0.93, -0.27]*	NA
		Dose of Tufuling	30-45g/day	1(100)	-0.68[-0.88, -0.48]*	NA
			≥45g/day	1(60)	-0.60[-0.93, -0.27]*	NA
		Combinations of Tufuling with high-frequency CHM	Tufuling-Huangbo	2(160)	-0.66[-0.83, -0.48]*	0.0
			Tufuling-Bixie	2(160)	-0.66[-0.83, -0.48]*	0.0
			Tufuling-Huangbo-Bixie-Niuxi	1(60)	-0.60[-0.93, -0.27]*	NA
	Effective rate	All studies	All studies	8(610)	1.10[1.02, 1.20]*	54.4%
		Risk of bias SG	Low risk of bias SG	5(406)	1.14[1.04, 1.24]*	5.8%
		Comparator Drug class	NSAIDs	6(470)	1.12[0.99, 1.26]	68.3%
			Colchicine	1(80)	1.12[0.95, 1.33]	NA
			NSAIDs&Colchicine	1(60)	1.07[0.94, 1.23]	NA
		UA level at baseline	<535μmol/L	5(390)	1.06[0.97, 1.16]	42.9%
			535-590μmol/L	2(120)	1.11[0.98, 1.27]	10.3%
		Treatment duration	≤7days	3(266)	1.12[0.88, 1.41]	85.1%
			7-14days	2(140)	1.05[0.95, 1.17]	0.0%
			≥14 days	3(204)	1.17[1.04, 1.31]*	0.0%
		Dose of Tufuling	<30g/day	2(166)	1.06[0.94, 1.20]	0.0%
			30-45g/day	5(384)	1.11[0.98, 1.27]	72.5%
			≥45g/day	1(60)	1.23[0.96, 1.57]	NA
		Combinations of Tufuling with high-frequency CHM	Tufuling-Huangbo	7(524)	1.11[1.00, 1.22]	61.2%
			Tufuling-Bixie	6(466)	1.11[1.00, 1.24]	65.8%
			Tufuling-Huangbo-Bixie-Niuxi	3(200)	1.06[0.92, 1.21]	63.9%
	Uric acid (μmol/L)	All studies	All studies	7(510)	-40.66[-68.21, -13.11]*	88.5%
		Risk of bias SG	Low risk of bias SG	4(306)	-57.35[-84.27, -30.43]*	80.8%
		comparator Drug class	NSAIDs	5(370)	-49.47[-78.62, -20.32]*	81.0%
			Colchicine	1(80)	-49.75[-68.05, -31.45]*	NA
			NSAIDs&Colchicine	1(60)	6.65[-14.93, 28.23]	NA
		UA level at baseline	<535μmol/L	5(390)	-50.87[-75.89, -25.84]*	81.8%
			535-590μmol/L	2(120)	-15.29[-64.49, 33.90]	77.2%
		Treatment duration	≤7days	2(166)	-54.32[-126.63, 17.99]	92.5%
			7-14days	2(140)	-14.98[-58.83, 28.87]	84.5%
			≥14 days	3(204)	-49.10[-63.71, -34.49]*	0.0%
		Dose of Tufuling	<30g/day	2(166)	-64.92[-115.18, -14.66]*	90.1%
			30-45g/day	4(284)	-27.34[-58.05, 3.38]	83.2%
			≥45g/day	1(60)	-44.00[-86.21, -1.79]*	NA
		Combinations of Tufuling with high-frequency CHM	Tufuling-Huangbo	6(424)	-31.33[-52.83, -9.82]*	73.2%
			Tufuling-Bixie	5(366)	-39.10[-77.18, -1.02]*	92.2%
			Tufuling-Huangbo-Bixie-Niuxi	3(200)	-13.19[-41.99, 15.60]	57.5%
	C-reactive protein (mg/L)	All studies	All studies	3(226)	-2.47[-8.29, 3.35]	95.3%
		Risk of bias SG	Low risk of bias SG	2(146)	-5.83[-7.63, -4.04]*	0.0%
		Comparator Drug class	NSAIDs	3(226)	-2.47[-8.29, 3.35]	95.3%
		UA level at baseline	<535μmol/L	2(166)	-2.54[-9.10, 4.01]	97.6%
			535-590μmol/L	1(60)	-2.10[-12.95, 8.75]	NA
		Treatment duration	≤7days	2(166)	-2.55[-9.10, 4.01]	97.6%
			≥14 days	1(60)	-2.10[-12.95, 8.75]	NA
		Dose of Tufuling	<30g/day	1(86)	-5.94[-7.76, -4.12]*	NA
			30-45g/day	1(80)	0.75[-0.12, 1.62]	NA
			≥45g/day	1(60)	-2.10[-12.95, 8.75]	NA
		Combinations of Tufuling with high-frequency CHM	Tufuling-Huangbo	2(140)	0.73[-0.14, 1.60]	0.0%
			Tufuling-Bixie	3(226)	-2.47[-8.29, 3.35]	95.3%
			Tufuling-Huangbo-Bixie-Niuxi	2(140)	0.73[-0.14, 1.60]	0.0%
	Erythrocyte sedimentation rate (mm/h)	All studies	All studies	5(366)	-3.49[-8.35, 1.38]	91.0%
		Risk of bias SG	Low risk of bias SG	3(226)	-5.13[-11.64, 1.38]	93.1%
		Comparator Drug class	NSAIDs	4(306)	-4.82[-10.12, 0.47]	89.8%
			NSAIDs&Colchicine	1(60)	0.98[-2.25, 4.21]	NA
		UA level at baseline	<535μmol/L	3(246)	-4.94[-10.75, 0.86]	93.2%
			535-590μmol/L	2(120)	0.67[-2.45, 3.78]	0.0%
		Treatment duration	≤7days	2(166)	-7.19[-12.21, -2.18]*	66.7%
			7-14days	2(140)	-0.60[-3.04, 1.84]	40.6%
			≥14 days	1(60)	-3.90[-16.17, 8.37]	NA
		Dose of Tufuling	<30g/day	2(166)	-5.38[-12.77, 2.01]	96.5%
			30-45g/day	2(140)	-0.78[-5.29, 3.73]	50.0%
			≥45g/day	1(60)	-3.90[-16.17, 8.37]	NA
		Combinations of Tufuling with high-frequency CHM	Tufuling-Huangbo	4(280)	-1.11[-2.80, 0.57]	0.0%
			Tufuling-Bixie	4(286)	-4.05[-10.53, 2.42]	90.1%
			Tufuling-Huangbo-Bixie-Niuxi	3(200)	-0.67[-3.95, 2.62]	14.5%
	White blood cell count (×10^9^/L	All studies	All studies	1(80)	-1.24[-2.07, -0.41]*	NA
		Risk of bias SG	Low risk of bias SG	1(80)	-1.24[-2.07, -0.41]*	NA
		Comparator Drug class	NSAIDs	1(80)	-1.24[-2.07, -0.41]*	NA
		UA level at baseline	<535μmol/L	1(80)	-1.24[-2.07, -0.41]*	NA
		Treatment duration	7-14days	1(80)	-1.24[-2.07, -0.41]*	NA
		Dose of Tufuling	<30g/day	1(80)	-1.24[-2.07, -0.41]*	NA
		Combinations of Tufuling with high-frequency CHM	Tufuling-Huangbo	1 (80)	-1.24[-2.07, -0.41]*	NA
**CHM plus Western medicine treatment vs. Western medicine treatment**	Visual analogue scale (VAS)	All studies	All studies	17(1351)	-0.94[-1.20, -0.68]*	93.2%
		Risk of bias SG	Low risk of bias SG	14(1015)	-0.97[-1.37, -0.57]*	93.8%
		Comparator Drug class	NSAIDs	16(1271)	-0.93[-1.20, -0.65]*	93.2%
			Allopurinol&Probenec	1(80)	-1.19[-1.45, -0.93]*	NA
		UA level at baseline	<535μmol/L	7(441)	-0.88[-1.47, -0.29]*	89.3%
			535-590μmol/L	9(760)	-1.08[-1.53, -0.63]*	94.6%
			>590μmol/L	1(150)	-0.62[-0.67, -0.57]*	NA
		Treatment duration	≤7days	9(616)	-1.11[-1.68, -0.54]*	95.2%
			≥14 days	8(735)	-0.83[-1.12, -0.55]*	87.5%
		Dose of Tufuling	<30g/day	5(437)	-1.06[-1.78, -0.34]*	96.9%
			30-45g/day	10(800)	-0.95[-1.39, -0.51]*	91.4%
			≥45g/day	2(114)	-0.69[-0.97, -0.40]*	32.8%
		Combinations of Tufuling with high-frequency CHM	Tufuling-Huangbo	13(1048)	-1.17[-1.56, -0.77]*	94.3%
			Tufuling-Bixie	12(962)	-1.10[-1.51, -0.70]*	95.1%
			Tufuling-Huangbo-Bixie-Niuxi	8(622)	-1.36[-1.97, -0.76]*	94.2%
	Effective rate	All studies	All studies	33(2597)	1.15[1.11, 1.18]*	0.0%
		Risk of bias SG	Low risk of bias SG	25(1888)	1.15[1.11, 1.19]*	0.0%
		Comparator Drug class	NSAIDs	24(1898)	1.16[1.12, 1.20]*	0.0%
			NSAIDs&Colchicine	5(398)	1.11[1.05, 1.19]*	0.0%
			NSAIDs&Allopurinol	1(80)	1.09[0.92, 1.30]	NA
			NSAIDs&Colchicine&Allopurinol	1(85)	1.15[0.97, 1.36]	NA
			Colchicine&Benzbromarone	1(92)	1.22[1.02, 1.45]*	NA
			NSAIDs&Benzbromarone	1(44)	1.17[0.94, 1.45]	NA
		UA level at baseline	<535μmol/L	13(946)	1.15[1.10, 1.22]*	0.0%
			535-590μmol/L	16(1262)	1.14[1.09, 1.19]*	0.0%
			>590μmol/L	3(329)	1.17[1.07, 1.27]*	0.0%
		Treatment duration	≤7days	16(1164)	1.11[1.07, 1.16]*	0.0%
			≥14 days	17(1433)	1.20[1.15, 1.25]*	0.0%
		Dose of Tufuling	<30g/day	9(817)	1.13[1.08,1.19]*	0.0%
			30-45g/day	20(1526)	1.16[1.12, 1.21]*	0.0%
			≥45g/day	4(254)	1.13[1.02, 1.25]*	0.0%
		Combinations of Tufuling with high-frequency CHM	Tufuling-Huangbo	27(2216)	1.14[1.11, 1.18]*	0.0%
			Tufuling-Bixie	24(1884)	1.15[1.11, 1.19]*	0.0%
			Tufuling-Huangbo-Bixie-Niuxi	18(1360)	1.14[1.09, 1.19]*	0.0%
	Uric acid (μmol/L)	All studies	All studies	33(2597)	-57.02[-66.31, -47.73]*	86.9%
		Risk of bias SG	Low risk of bias SG	25(1888)	-52.36[-60.26, -44.47]*	74.6%
		comparator Drug class	NSAIDs	25(1958)	-59.86[-70.90, -48.83]*	89.5%
			NSAIDs&Colchicine	4(338)	-48.14[-69.08, -27.21]*	44.3%
			NSAIDs&Allopurinol	1(80)	-48.75[-60.95, -36.56]*	NA
			NSAIDs&Colchicine&Allopurinol	1(85)	-42.22[-80.37, -4.07]*	NA
			Colchicine&Benzbromarone	1(92)	-41.65[-76.94, -6.36]*	NA
			NSAIDs&Benzbromarone	1(44)	-34.49[-74.08, 5.10]	NA
		UA level at baseline	<535μmol/L	14(1006)	-64.50[-81.17, -47.83]*	90.7%
			535-590μmol/L	16(1262)	-49.09[-56.88, -41.31]*	56.0%
			>590μmol/L	3(329)	-53.89[-80.40, -27.37]*	64.3%
		Treatment duration	≤7days	16(1164)	-59.20[-74.32, -44.07]*	91.9%
			≥14 days	17(1433)	-54.09[-63.80, -44.38]*	67.2%
		Dose of Tufuling	<30g/day	10(877)	-57.35[-69.94, -44.75]*	69.6%
			30-45g/day	20(1526)	-58.60[-72.30, -44.91]*	89.4%
			≥45g/day	3(194)	-40.19[-47.23, -33.16]*	0.0%
		Combinations of Tufuling with high-frequency CHM	Tufuling-Huangbo	28(2216)	-58.71[-69.01, -48.41]*	84.8%
			Tufuling-Bixie	24(1884)	-65.01[-75.60, -54.43]*	83.9%
			Tufuling-Huangbo-Bixie-Niuxi	17(1300)	-56.09[-62.05, -50.13]*	23.4%
	C-reactive protein (mg/L)	All studies	All studies	31(2418)	-4.49[-5.31, -3.68]*	87.9%
		Risk of bias SG	Low risk of bias SG	24(1789)	-4.93[-6.01, -3.86]*	87.7%
		Comparator Drug class	NSAIDs	23(1799)	-5.17[-6.32, -4.03]*	89.0%
			NSAIDs&Colchicine	5(398)	-4.20[-6.64, -1.77]*	81.1%
			NSAIDs&Colchicine&Allopurinol	1(85)	-2.94[-4.65, -1.23]*	NA
			Colchicine&Benzbromarone	1(92)	-1.77[-2.18, -1.36]*	NA
			NSAIDs&Benzbromarone	1(44)	-1.63[-3.27, 0.01]	NA
		UA level at baseline	<535μmol/L	13(937)	-5.59[-7.37, -3.81]*	90.3%
			535-590μmol/L	14(1092)	-4.04[-5.24, -2.84]*	82.9%
			>590μmol/L	3(329)	-4.88[-6.83, -2.93]*	74.7%
		Treatment duration	≤7days	15(1075)	-5.41[-7.03, -3.79]*	91.4%
			≥14 days	16(1343)	-4.00[-5.03, -2.97]*	83.0%
		Dose of Tufuling	<30g/day	9(797)	-4.76[-6.58, -2.94]*	91.9%
			30-45g/day	18(1367)	-4.74[-6.01, -3.47]*	86.8%
			≥45g/day	4(254)	-3.77[-6.19, -1.34]*	83.2%
		Combinations of Tufuling with high-frequency CHM	Tufuling-Huangbo	26(2037)	-4.48[-5.40, -3.55]*	88.5%
			Tufuling-Bixie	22(1705)	-5.13[-6.43, -3.82]*	89.6%
			Tufuling-Huangbo-Bixie-Niuxi	15(1121)	-5.26[-7.18, -3.33]*	88.9%
	Erythrocyte sedimentation rate (mm/h)	All studies	All studies	31(2418)	-6.06[-7.22, -4.90]*	83.2%
		Risk of bias SG	Low risk of bias SG	24(1789)	-6.30[-7.77, -4.84]*	86.8%
		Comparator Drug class	NSAIDs	23(1799)	-6.79[-8.21, -5.37]*	83.0%
			NSAIDs&Colchicine	5(398)	-3.75[-4.86, -2.63]*	0.0%
			NSAIDs&Colchicine&Allopurinol	1(85)	-3.71[-5.42, -2.00]*	NA
			Colchicine&Benzbromarone	1(92)	-4.56[-6.19, -2.93]*	NA
			NSAIDs&Benzbromarone	1(44)	-4.93[-7.17, -2.69]*	NA
		UA level at baseline	<535μmol/L	13(937)	-5.73[-7.63, -3.83]*	82.7%
			535-590μmol/L	14(1092)	-7.06[-8.91, -5.20]*	82.4%
			>590μmol/L	3(329)	-4.84[-6.72, -2.97]*	64.9%
		Treatment duration	≤7days	15(1075)	-6.19[-8.09, -4.30]*	83.0%
			≥14 days	16(1343)	-6.00[-7.51, -4.49]*	83.8%
		Dose of Tufuling	<30g/day	9(797)	-5.06[-6.61, -3.51]*	73.4%
			30-45g/day	18(1367)	-6.64[-8.21, -5.07]*	81.7%
			≥45g/day	4(254)	-5.42[-10.18, -0.67]*	89.6%
		Combinations of Tufuling with high-frequency CHM	Tufuling-Huangbo	26(2037)	-5.35[-6.33, -4.37]*	67.4%
			Tufuling-Bixie	22(1705)	-5.80[-6.96, -4.64]*	68.7%
			Tufuling-Huangbo-Bixie-Niuxi	15(1121)	-6.00[-7.66, -4.34]*	77.2%
	White blood cell count (×10^9^/L	All studies	All studies	5(342)	-0.78[-1.88, 0.32]	89.9%
		Risk of bias SG	Low risk of bias SG	5(342)	-0.78[-1.88, 0.32]	89.9%
		Comparator Drug class	NSAIDs	5(342)	-0.78[-1.88, 0.32]	89.9%
		UA level at baseline	<535μmol/L	2(119)	-1.29[-2.59, 0.01]	92.9%
			535-590μmol/L	3(223)	0.30[-0.92, 1.51]	32.2%
		Treatment duration	≤7days	3(179)	-0.22[-1.26, 0.81]	64.5%
			≥14 days	2(163)	-1.55[-3.69, 0.59]	95.2%
		Dose of Tufuling	30-45g/day	5(342)	-0.78[-1.88, 0.32]	89.9%
		Combinations of Tufuling with high-frequency CHM	Tufuling-Huangbo	4(250)	-0.85[-2.26, 0.57]	91.6%
			Tufuling-Bixie	3(190)	-0.82[-3.20, 1.56]	92.8%
			Tufuling-Huangbo-Bixie-Niuxi	3(190)	-0.82[-3.20, 1.56]	92.8%
	TCM Syndrome Scoring	All studies	All studies	16(1134)	-3.18[-4.11, -2.25]*	98.1%
		Risk of bias SG	Low risk of bias SG	14(1014)	-3.22[-4.24, -2.20]*	98.4%
		Comparator Drug class	NSAIDs	13(926)	-3.66[-4.58, -2.74]*	95.9%
			NSAIDs&Benzbromarone	1(44)	-1.56[-2.13, -0.99]*	NA
			NSAIDs&Colchicine	2(164)	-0.96[-2.53, 0.61]	71.3%
		UA level at baseline	<535μmol/L	9(640)	-3.57[-4.80, -2.33]*	94.5%
			535-590μmol/L	7(494)	-2.68[-3.85, -1.51]*	97.6%
		Treatment duration	≤7days	11(781)	-2.80[-3.63, -1.97]*	95.2%
			≥14 days	5(353)	-3.43[-4.92, -1.94]*	93.8%
		Dose of Tufuling	<30g/day	4(311)	-2.04[-3.30, -0.78]*	97.8%
			30-45g/day	12(823)	-3.42[-4.40, -2.43]*	93.6%
		Combinations of Tufuling with high-frequency CHM	Tufuling-Huangbo	13(895)	-2.86 [-3.89, -1.83]*	98.4%
			Tufuling-Bixie	12(811)	-3.14[-4.05, -2.24]*	96.4%
			Tufuling-Huangbo-Bixie-Niuxi	8(535)	-2.82[-3.92, -1.72]*	97.6%
**CHM comprehensive therapy plus Western medicine treatment vs. Western medicine treatment**	Visual analogue scale (VAS)	All studies	All studies	4(413)	-1.13[-2.14, -0.12]*	99.2%
		Risk of bias SG	Low risk of bias SG	4(413)	-1.13[-2.14, -0.12]*	99.2%
		Comparator Drug class	NSAIDs	2(216)	-1.54[-3.29, 0.20]	99.6%
			Colchicine	1(136)	-0.61[-0.81, -0.41]*	NA
			NSAIDs&Colchicine	1(61)	-0.82[-1.07, -0.57]*	NA
		UA level at baseline	<535μmol/L	2(121)	-0.71[-0.86, -0.55]*	16.1%
			535-590μmol/L	1(156)	-2.43[-2.57, -2.29]*	NA
		Treatment duration	≤7days	3(277)	-1.30[-2.57, -0.04]*	99.3%
			≥14 days	1(136)	-0.61[-0.81, -0.41]*	NA
		Dose of Tufuling	<30g/day	2(197)	-0.70[-0.90, -0.50]*	37.9%
			30-45g/day	2(216)	-1.54[-3.29, 0.20]	99.6%
		Combinations of Tufuling with high-frequency CHM	Tufuling-Huangbo	4(413)	-1.13[-2.14, -0.12]*	99.2%
			Tufuling-Bixie	3(352)	-1.23[-2.50, 0.04]	99.4%
			Tufuling-Huangbo-Bixie-Niuxi	3(352)	-1.23[-2.50, 0.04]	99.4%
	Effective rate	All studies	All studies	6(580)	1.11[1.05, 1.18]*	0.0%
		Risk of bias SG	Low risk of bias SG	6(580)	1.11[1.05, 1.18]*	0.0%
		Comparator Drug class	NSAIDs	3(303)	1.10[1.02, 1.18]*	0.0%
			Colchicine	1(136)	1.16[0.98, 1.36]	NA
			Febuxostat	1(80)	1.28[1.03, 1.57]*	NA
			NSAIDs&Colchicine	1(61)	1.08[0.89, 1.32]	NA
		UA level at baseline	<535μmol/L	4(288)	1.17[1.07, 1.28]*	0.0%
			535-590μmol/L	1(156)	1.07[0.99, 1.15]	NA
		Treatment duration	≤7days	4(357)	1.13[1.02, 1.24]*	35.0%
			7-14days	1(87)	1.14[0.99, 1.32]	NA
			≥14 days	1(136)	1.16[0.98, 1.37]	NA
		Dose of Tufuling	<30g/day	3(277)	1.16[1.04, 1.30]*	0.0%
			30-45g/day	3(303)	1.10[1.03, 1.18]*	8.2%
		Combinations of Tufuling with high-frequency CHM	Tufuling-Huangbo	5(493)	1.12[1.04, 1.21]*	18.5%
			Tufuling-Bixie	3(352)	1.11[1.02, 1.21]*	23.6%
			Tufuling-Huangbo-Bixie-Niuxi	3(352)	1.11[1.02, 1.21]*	23.6%
	Uric acid (μmol/L)	All studies	All studies	5(457)	-86.11[-109.72, -62.51]*	95.3%
		Risk of bias SG	Low risk of bias SG	5(457)	-86.11[-109.72, -62.51]*	95.3%
		comparator Drug class	NSAIDs	2(216)	-75.81[-216.15, 64.53]	96.8%
			Febuxostat	1(80)	-57.97[-79.17, -36.77]*	NA
			NSAIDs&Colchicine	2(161)	-90.83[-96.18, -85.49]*	26.2%
		UA level at baseline	<535μmol/L	4(301)	-76.26[-92.46, -60.06]*	86.5%
			535-590μmol/L	1(156)	-145.44[-158.85, -132.03]*	NA
		Treatment duration	≤7days	4(357)	-80.43[-120.96, -39.89]*	96.1%
			≥14 days	1(100)	-88.58[-94.28, -82.88]*	NA
		Dose of Tufuling	<30g/day	2(141)	-77.47[-112.80, -42.14]*	90.0%
			30-45g/day	3(316)	-85.26[-136.00, -34.52]*	97.3%
		Combinations of Tufuling with high-frequency CHM	Tufuling-Huangbo	5(457)	-86.11[-109.72, -62.51]*	95.3%
			Tufuling-Bixie	3(316)	-85.26[-136.00, -34.52]*	97.3%
			Tufuling-Huangbo-Bixie-Niuxi	3(316)	-85.26[-136.00, -34.52]*	97.3%
	C-reactive protein (mg/L)	All studies	All studies	5(457)	-4.48[-7.22, -1.74]*	97.7%
		Risk of bias SG	Low risk of bias SG	5(457)	-4.48[-7.22, -1.74]*	97.7%
		Comparator Drug class	NSAIDs	3(303)	-4.65[-7.43, -1.86]*	88.4%
			Febuxostat	1(80)	-5.11[-6.54, -3.68]*	NA
			NSAIDs&Colchicine	2(161)	-4.07[-9.52, 1.38]	99.0%
		UA level at baseline	<535μmol/L	5(388)	-4.10[-7.17, -1.02]*	97.5%
			535-590μmol/L	1(156)	-6.01[-7.10, -4.92]*	NA
		Treatment duration	≤7days	4(357)	-5.37[-6.82, -3.92]*	81.2%
			≥14 days	1(100)	-1.31[-1.62, -1.00]*	NA
		Dose of Tufuling	<30g/day	2(141)	-6.06[-7.78, -4.34]*	73.4%
			30-45g/day	3(316)	-3.47[-6.66, -0.29]*	97.1%
		Combinations of Tufuling with high-frequency CHM	Tufuling-Huangbo	5(457)	-4.48[-7.22, -1.74]*	97.7%
			Tufuling-Bixie	3(316)	-3.47[-6.66, -0.29]*	97.1%
			Tufuling-Huangbo-Bixie-Niuxi	3(316)	-3.47[-6.66, -0.29]*	97.1%
	Erythrocyte sedimentation rate (mm/h)	All studies	All studies	6(580)	-7.04[-9.33, -4.75]*	93.7%
		Risk of bias SG	Low risk of bias SG	6(580)	-7.04[-9.33, -4.75]*	93.7%
		Comparator Drug class	NSAIDs	3(303)	-7.94[-11.95, -3.93]*	96.8%
			Colchicine	1(136)	-5.10[-6.77, -3.43]*	NA
			Febuxostat	1(80)	-7.71[-9.87, -5.55]*	NA
			NSAIDs&Colchicine	1(61)	-5.56[-7.12, -4.00]*	NA
		UA level at baseline	<535μmol/L	4(288)	-7.30[-11.29, -3.30]*	95.7%
			535-590μmol/L	1(156)	-7.88[-8.64, -7.12]*	NA
		Treatment duration	≤7days	4(357)	-6.20[-8.31, -4.10]*	88.8%
			7-14days	1(87)	-12.27[-13.74, -10.81]*	NA
			≥14 days	1(136)	-5.10[-6.77, -3.43]*	NA
		Dose of Tufuling	<30g/day	3(277)	-5.97[-7.37, -4.56]*	46.8%
			30-45g/day	3(303)	-7.94[-11.95, -3.93]*	96.8%
		Combinations of Tufuling with high-frequency CHM	Tufuling-Huangbo	5(493)	-5.99[-7.76, -4.22]*	86.9%
			Tufuling-Bixie	3(352)	-5.61[-8.40, -2.81]*	92.8%
			Tufuling-Huangbo-Bixie-Niuxi	3(352)	-5.61[-8.40, -2.81]*	92.8%
	TCM Syndrome Scoring	All studies	All studies	2(147)	-3.97[-4.62, -3.33]*	0.0%
		Risk of bias SG	Low risk of bias SG	2(147)	-3.97[-4.62, -3.33]*	0.0%
		Comparator Drug class	NSAIDs	2(147)	-3.97[-4.62, -3.33]*	0.0%
		UA level at baseline	535-590μmol/L	2(147)	-3.97[-4.62, -3.33]*	0.0%
		Treatment duration	≤7days	1(60)	-3.90[-4.59, -3.21]*	NA
			7-14days	1(87)	-4.51[-6.38, -2.64]*	NA
		Dose of Tufuling	30-45g/day	2(147)	-3.97[-4.62, -3.33]*	0.0%
		Combinations of Tufuling with high-frequency CHM	Tufuling-Huangbo	1(60)	-3.90[-4.59, -3.21]*	NA
			Tufuling-Bixie	1(60)	-3.90[-4.59, -3.21]*	NA
			Tufuling-Huangbo-Bixie-Niuxi	1(60)	-3.90[-4.59, -3.21]*	NA

*Statistically significant difference between groups. Bold values indicate statistically significant results (P < 0.05).

CI, confidence interval; CHM, Chinese herbal medicine; MD, mean difference; RR, Relative Risk; SG, sequence generation; NSAIDs, Non-Steroidal Anti-Inflammatory Drugs.

###### 3.1.3.1.2 Effective rate

A pooled analysis of seven studies (33–36, 43, 46, 55) comprising 658 patients evaluated the efficacy of CHM monotherapy versus conventional WM in improving effective response rates in patients with gout. The overall results indicated the superiority of the CHM monotherapy group (RR 1.06 [95% CI 1.01–1.11], I²=0.0%). However, in the prespecified subgroup analyses, no superiority of CHM monotherapy was observed in subgroups defined by a low risk of bias in random sequence generation (33–36, 43, 46, 55), control groups receiving NSAIDs (33, 46), controls receiving allopurinol and probenecid (55), participants with baseline uric acid levels of<535μmol/L (35, 36, 46, 55) or 535–590 μmol/L (34, 43), treatment duration ≤7 days (33, 46) or 7–14 days (43), *Tufuling* dose <30 g/day (43) or 30–45 g/day (33–36, 46, 55), and combinations of *Tufuling* with high-frequency CHM including *Huangbo (*33–36, 43, 46), *Bixie (*15, 33, 35, 36, 43, 46), or *Huangbo*-*Bixie*-*Niuxi (*34, 43) (**Table 3**).

**Figure 5:**
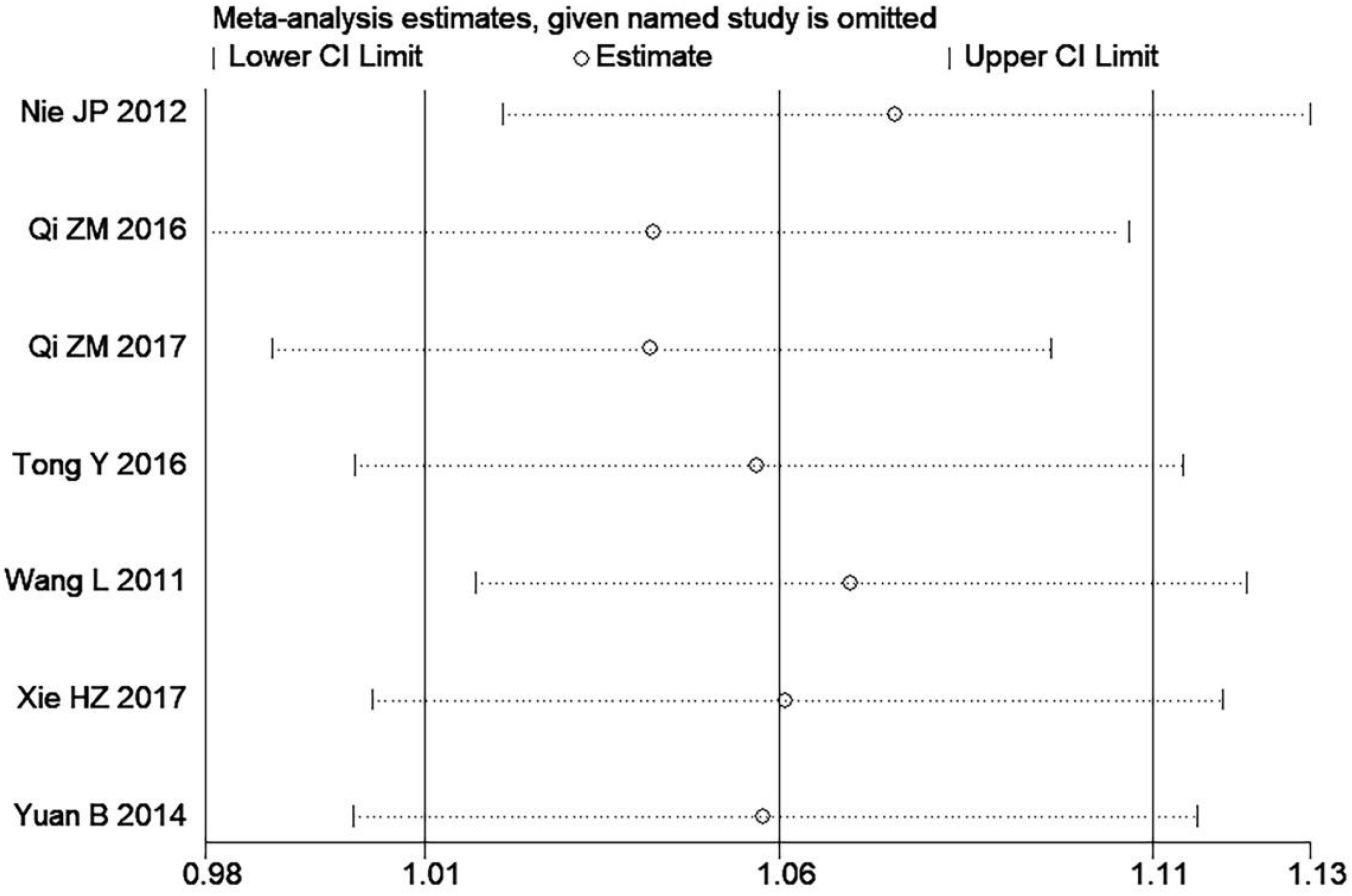
Results of the effective rate sensitivity analysis in the CHM vs. WM group.

The leave-one-out sensitivity analysis yielded RR estimates between 0.98 and 1.13 for all studies. The results were generally robust, although sensitive to individual studies; most recalculated CIs excluded 1. Particular attention is warranted for the Wang L 2011 (46) study, whose exclusion might shift the point estimate near or below 1 and widen the CI to include 1, potentially negating the statistical significance of CHM superiority (**Figure 5**).

###### 3.1.3.1.3 Uric acid

**Figure 6:**
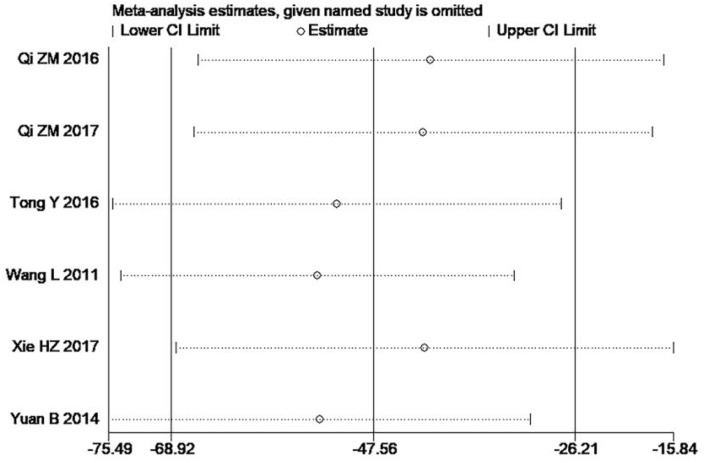
Results of the UA sensitivity analysis in the CHM vs. WM group.

A meta-analysis of six studies (34–36, 43, 46, 55) (568 patients) indicated that CHM alone was superior to conventional WM in reducing UA levels [MD -47.56 (-68.92, -26.21), I^2^ = 81.1%]. Subgroup analysis indicated no significant advantage for CHM when the control intervention was NSAIDs [MD 8.9 μmol/L, 95% CI (-44.00, 61.80)] (46). In all other prespecified subgroups, CHM monotherapy had a favorable effect on prognosis. Notably, the treatment effect demonstrated the greatest consistency (I²= 0%) in the subgroup of patients with moderately elevated baseline UA levels (535–590 μmol/L) (34, 43), treatment duration 7-14days (43), and combinations of *Tufuling* with *Huangbo*-*Bixie*-*Niuxi (*34, 43) (**Table 3**). Sensitivity analysis confirmed high robustness, as the MD remained within a negative range of -75.49–15.84 μmol/L upon exclusion of any single study, with all CIs excluding zero (**Figure 6**).

###### 3.1.3.1.4 C-reactive protein

**Figure 7:**
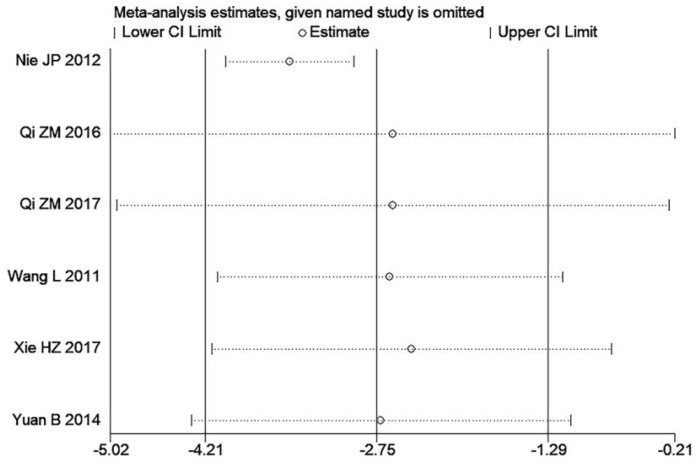
Results of the CRP sensitivity analysis in the CHM vs. WM group.

Pooled results from six studies (33–36, 46, 55) (618 patients) favored CHM alone over WM for CRP reduction [MD -2.75 (-4.21, -1.29), I^2^ = 76.6%]. However, in prespecified subgroup with baseline UA levels of 535–590 μmol/L, control groups receiving NSAIDs (33, 46), participants with baseline uric acid levels of 535–590 μmol/L (34), treatment duration ≤7 days (33, 46), and combinations of *Tufuling* with *Huangbo*-*Bixie*-*Niuxi (*34), CHM alone was not superior to WM (**Table 3**). The findings were robust, with MD estimates ranging from -5.02 to -0.21 mg/L in the sensitivity analysis, and all recalculated CIs excluded zero (**Figure 7**).

###### 3.1.3.1.5 Erythrocyte sedimentation rate

**Figure 8:**
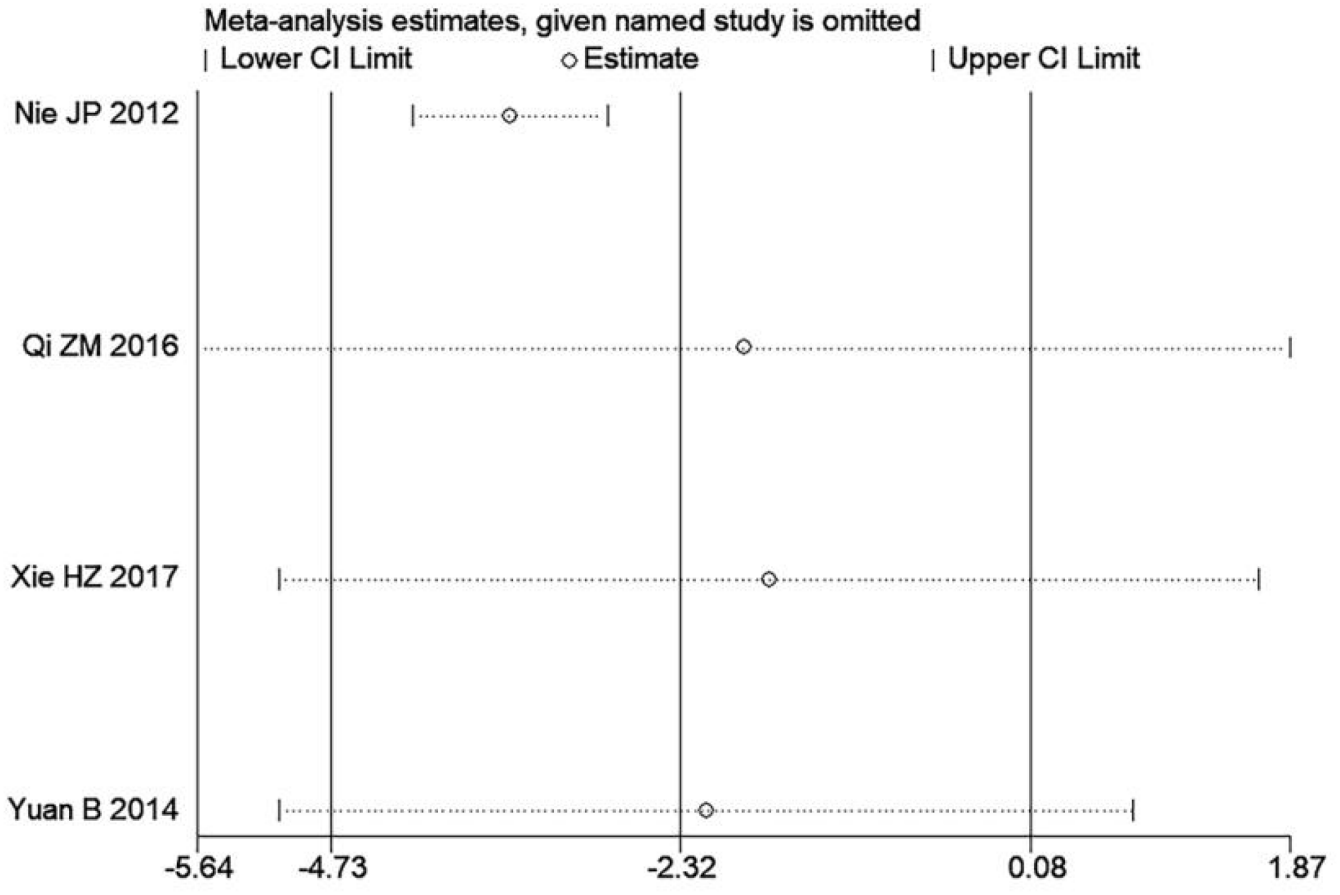
Results of the ESR sensitivity analysis in the CHM vs. WM group.

Analysis of four studies (33–35, 55) (440 patients) revealed that CHM alone was superior to WM in lowering the ESR [MD -5.76 (-11.018, -0.496), I^2^ = 91.9%]. This benefit was consistent across most prespecified subgroups, except when the control intervention was an NSAIDs (33) or in the ‘Low risk of bias’ subgroup (33–35), treatment duration ≤ 7days (33), combinations of *Tufuling* with *Huangbo (*33–35) or *Bixie (*33–35) where no significant advantage was observed (**Table 3**). Sensitivity analysis revealed that the MD varied between -5.64 and 1.87 mm/h. Given that the original CI’s upper limit was close to zero, the conclusion was sensitive to the included studies (**Figure 8**).

###### 3.1.3.1.6 White blood cell count

In one study (43) (40 patients) with a low risk of bias, colchicine was used as a control, patients with UA levels ranging from 535–590 μmol/L, treatment duration was 10 days, dose of *Tufuling* was 15g/day, and combinations of *Tufuling* with *Tufuling*-*Huangb*o-*Bixie*-*Niuxi* were included. CHM alone was superior to WM in reducing WBC count [MD -2.00 (-3.21, -0.79), I^2^=NA] (**Table 3**).

###### 3.1.3.1.7 TCM syndrome scoring

One study (46) (58 patients) with a low risk of bias that used NSAIDs as controls, patients with UA levels <535 μmol/L,treatment duration was 7 days, dose of *Tufuling* was 30g/day, and combinations of *Tufuling* with *Huangb*o-*Bixie* reported no significant difference between CHM alone and WM in improving TCM syndrome scores [MD 0.00 (-2.20, 2.20), I^2^=NA] (**Table 3**).

##### 3.1.3.2 CHM comprehensive therapy vs. Western medicine treatment

###### 3.1.3.2.1 Visual analog scale

**Figure 9:**
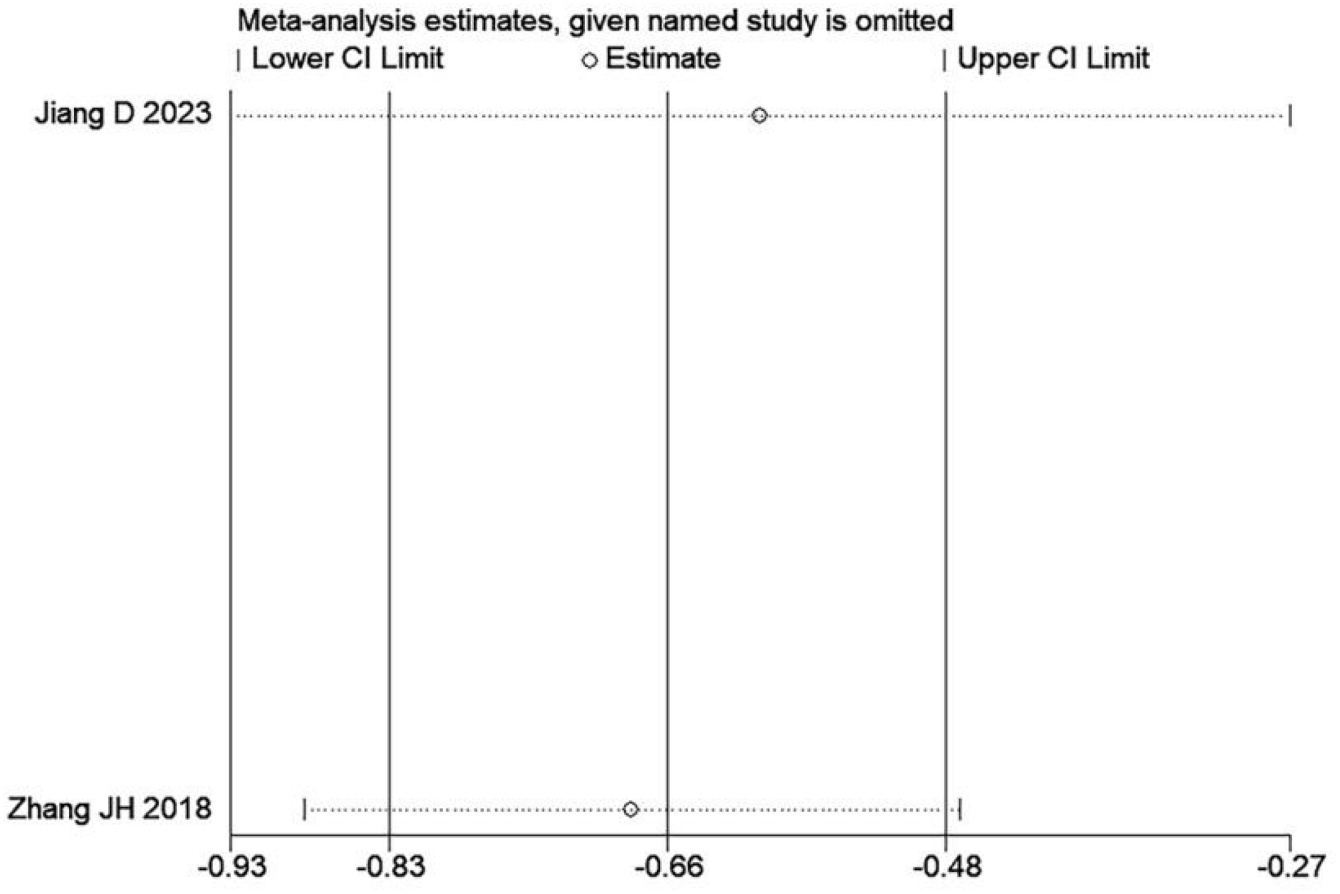
Results of the VAS sensitivity analysis in the CHM comprehensive therapy group.

Two randomized controlled trials (19, 66) involving 160 participants compared the efficacy of traditional Chinese medicine (TCM) comprehensive therapy with that of conventional WM in improving VAS scores. Pooled analysis revealed superior pain reduction in the TCM comprehensive therapy group [MD: -0.66 (-0.83, -0.48), I^2^ = 0.0%]. In all prespecified subgroups, CHM comprehensive therapy had a favorable effect on prognosis. (**Table 3**). Sensitivity analysis (excluding Zhang JH 2018) (66) revealed a narrow MD range of -0.93 to -0.27, indicating stable results (**Figure 9**).

###### 3.1.3.2.2 Effective rate

**Figure 10:**
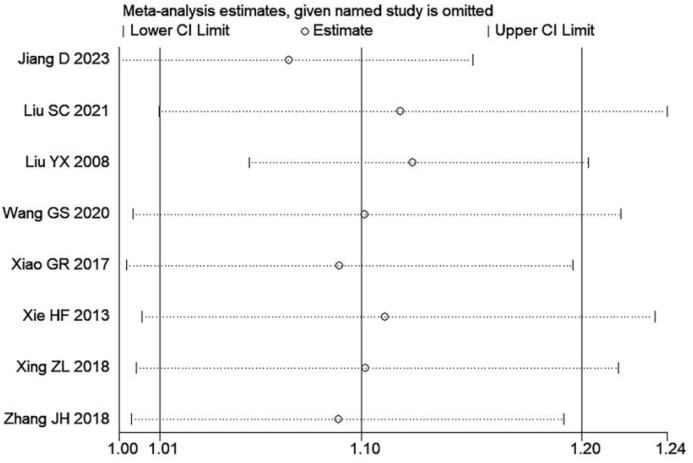
Results of the Effective rate sensitivity analysis in the CHM comprehensive therapy group.

Based on eight studies (19, 27, 29, 44, 53, 54, 56, 66) (610 patients), comprehensive CHM therapy had a higher effective rate than WM [RR: 1.10 [1.02, 1.20], I^2^ = 54.4%]. This benefit was consistent with the low risk of bias subgroup [RR: 1.14 [1.04, 1.24], I²=5.8%] (19, 27, 44, 56, 66) and treatment duration ≥14 days subgroup[RR:1.17 [1.04, 1.31], I²=0.0%] (44, 53, 66); however, no superiority of comprehensive Chinese herbal medicine therapy was evident in other subgroups (e.g., controls: NSAIDs [RR: 1.12 [0.99, 1.26], I^2^ = 68.3%] (19, 27, 29, 53, 56, 66), colchicine [RR: 1.12 [0.95, 1.33], I^2^=NA] (44), NSAIDs+colchicine [RR: 1.07 [0.94, 1.23], I^2^=NA] (54), UA 535–590 μmol/L [RR: 1.11 [0.98, 1.27], I^2^ = 10.3%] (54, 66), UA <535 μmol/L [RR: 1.06 [0.97, 1.16], I^2^ = 42.9%] (27, 29, 44, 53, 56), treatment duration ≤ 7days[RR: 1.12[0.88, 1.41], I^2^ = 85.1%] (19, 29, 56) or 7-14days[RR: 1.05[0.95, 1.17], I^2^ = 0.0%] (27, 54) and dose of *Tufuling*, combinations of *Tufuling* with high-frequency CHM subgroup) (**Table 3**). Sensitivity analysis revealed high robustness, with RR point estimates consistently >1 (range: 1.00–1.24) and minimal fluctuation (**Figure 10**).

###### 3.1.3.2.3 Uric acid

**Figure 11:**
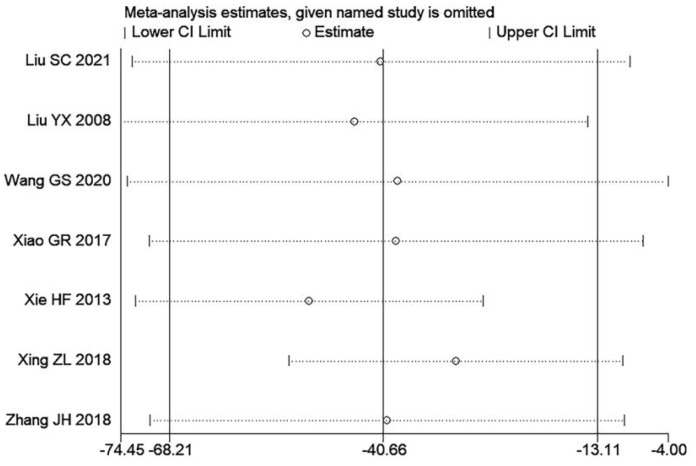
Results of the UA sensitivity analysis in the CHM comprehensive therapy group.

A meta-analysis of seven studies (27, 29, 44, 53, 54, 56, 66) (510 patients) indicated that comprehensive CHM therapy was superior to WM for UA reduction [MD:-40.66 (-68.21, -13.11, I^2^ = 88.5%)]. Consistent treatment effects were observed across multiple prespecified subgroups, with reduced heterogeneity noted in these analyses: low risk of bias subgroup [MD:-57.35 (-84.27, -30.43),I^2^ = 80.8%] (27, 44, 56, 66); NSAIDs-controlled trials [MD:-49.47 (-78.62, -20.32),I^2^ = 81.0%] (27, 29, 53, 56, 66); colchicine-controlled trials [MD:-49.75 (-68.05, -31.46),I^2^=NA] (44); baseline uric acid <535 μmol/L subgroup [MD:-50.87 (-75.89, -25.84),I^2^ = 81.8%] (27, 29, 44, 53, 56); treatment duration ≥14 days [MD -49.10 (-63.71, -34.49), I²=0.0%] (44, 53, 66); *Tufuling* dose <30 g/day [MD -64.92 (-115.18, -14.66), I²=90.1%] (27, 56); *Tufuling* dose ≥45 g/day [MD -44.00 (-86.21, -1.79), I²=NA] (66); *Tufuling*-*Huangbo* combination [MD -31.33 (-52.83, -9.82), I²=73.2%] (27, 29, 44, 53, 54, 66); and *Tufuling*-*Bixie* combination [MD -39.10 (-77.18, -1.02), I²=92.2%] (29, 44, 54, 56, 66). However, in the prespecified subgroup analyses, no statistically significant advantage for TCM comprehensive therapy was detected in trials using NSAIDs and colchicine combination therapy as controls [MD: 6.65 (-14.93, 28.23),I^2^=NA] (54) and patients with baseline uric acid levels of 535-590 μmol/L [MD: -15.29 (-64.49, 33.90),I²=77.2%]; treatment duration ≤7 days [MD -54.32 (-126.63, 17.99), I²=92.5%] (29, 56); treatment duration 7–14 days [MD -14.98 (-58.83, 28.87), I²=84.5%] (27, 54); *Tufuling* dose 30-45g/day [MD -27.34 (-58.05, 3.38), I²=83.2%] (29, 44, 53, 54); and *Tufuling*-*Huangbo*-*Bixie*-*Niuxi* combination [MD -13.19 (-41.99, 15.60), I²=57.5%] (29, 54, 66) (**Table 3**). Sensitivity analysis indicated low robustness for the point estimate magnitude, as the MD varied widely from -74.45 to -4.00 μmol/L, indicating high sensitivity to specific studies (e.g., Liu SC 2021 (27), Wang GS 2020 (44)) (**Figure 11**).

###### 3.1.3.2.4 C-reactive protein

**Figure 12:**
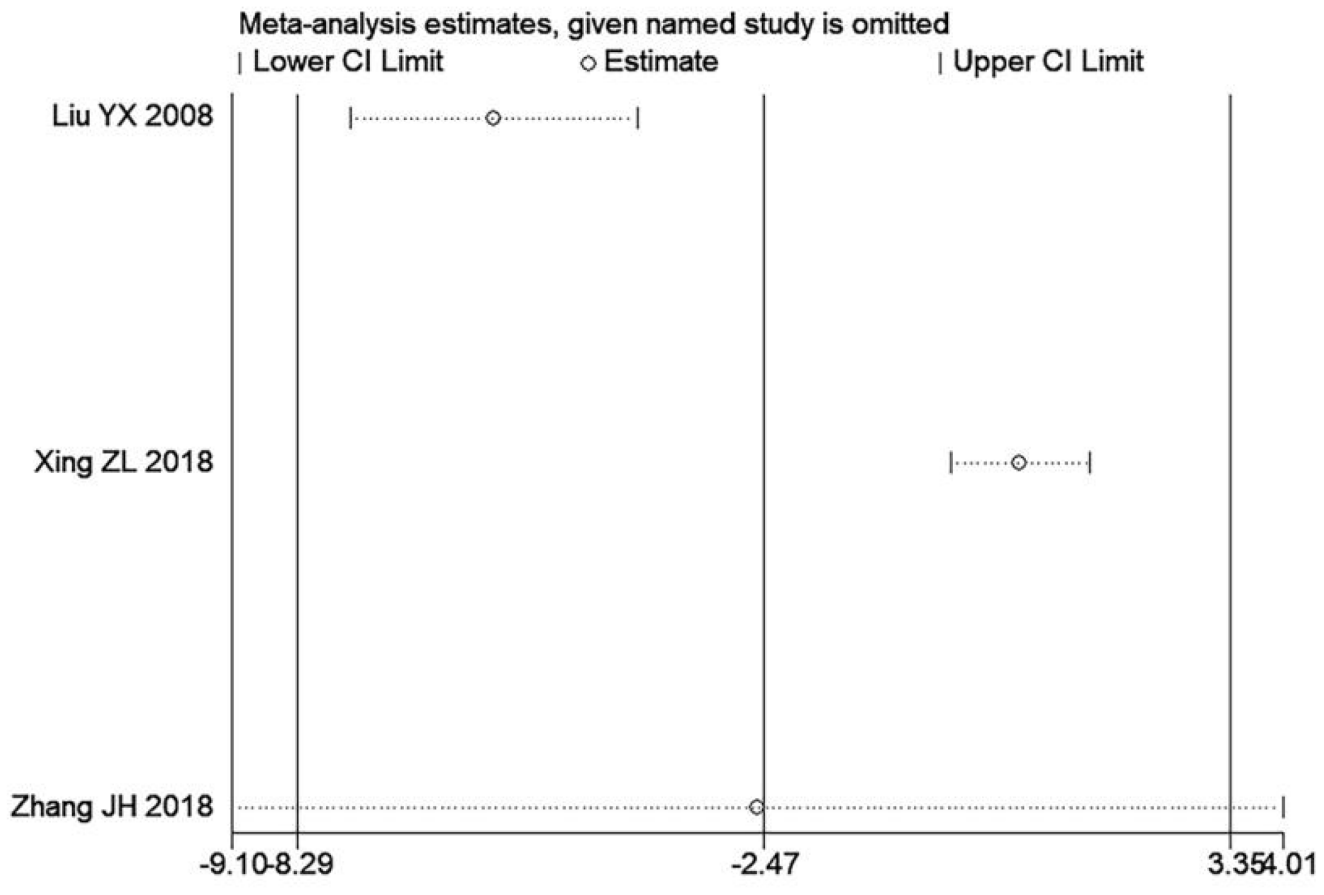
Results of the CRP sensitivity analysis in the CHM comprehensive therapy group.

Pooled results from three studies (29, 56, 66) (226 patients) revealed no significant difference between comprehensive CHM therapy and NSAIDs for CRP reduction [MD:-2.47 (-8.29, 3.35), I^2^ = 95.3%], which was consistent across UA level, treatment duration and combinations of *Tufuling* with high-frequency CHM subgroups. However, in the ‘Low risk of bias’ [MD: -5.83 (-7.63, -4.04), I^2^ = 0.0%] (56, 66),and Dose of *Tufuling*<30g/day[MD:-5.94(-7.76, -4.12),I^2^=NA] (56) subgroup, comprehensive CHM was superior(**Table 3**). The sensitivity analysis suggested good robustness, with MD estimates varying between -2.47 and 4.01 mg/L (**Figure 12**).

###### 3.1.3.2.5 Erythrocyte sedimentation rate

**Figure 13:**
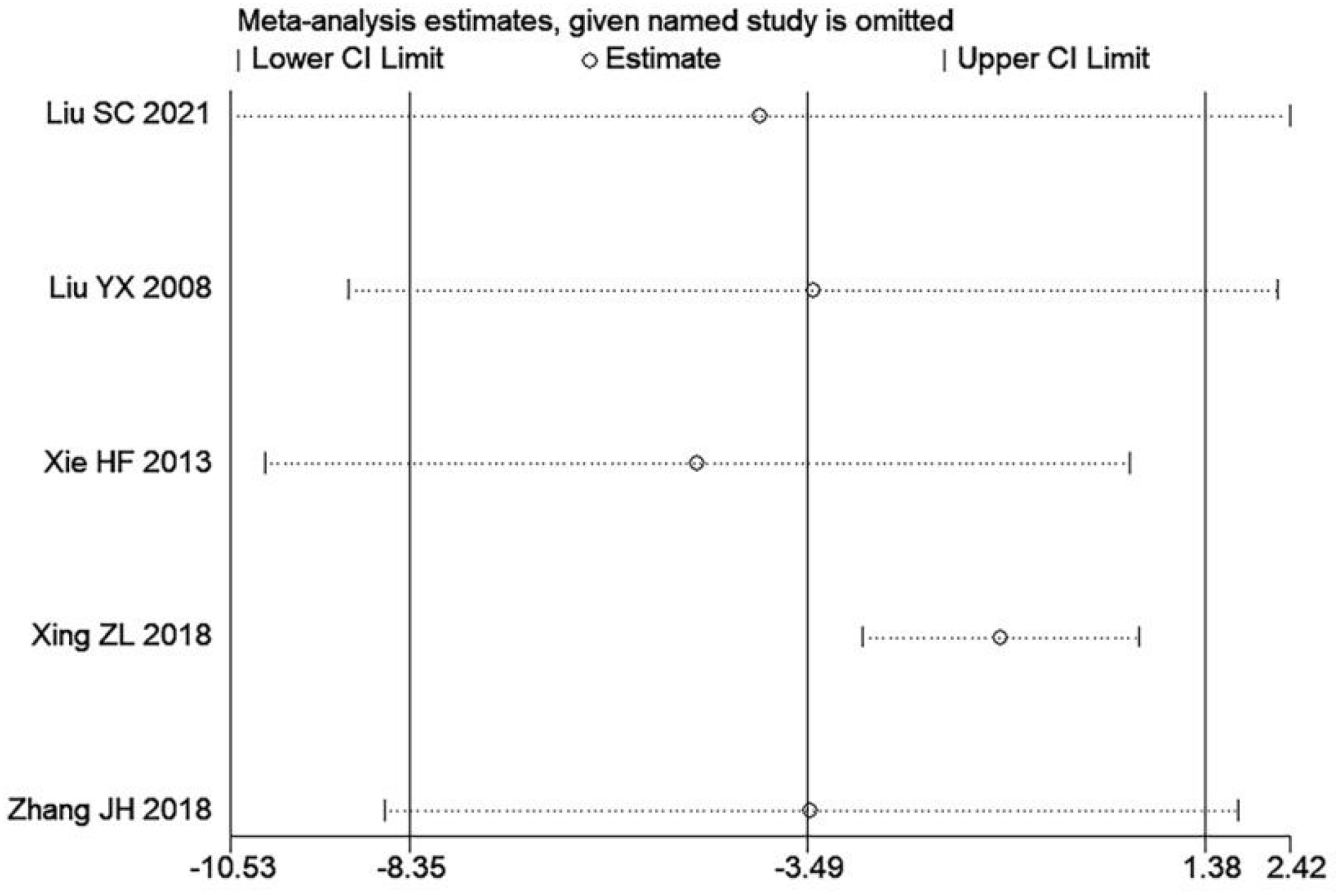
Results of the ESR sensitivity analysis in the CHM comprehensive therapy group.

Analysis of five studies (27, 29, 54, 56, 66) (366 patients) revealed no significant difference between comprehensive CHM therapy and WM for ESR reduction [MD: -3.49 (-8.35, 1.38), I^2^ = 91.0%]. Subgroup analyses yielded consistent null findings, with the exception of the treatment duration ≤7 days subgroup, where comprehensive CHM therapy demonstrated superiority [MD -7.19 (-12.21, -2.18), I²=66.7%] (29, 56). (**Table 3**). The sensitivity analysis showed limited robustness, with MD estimates ranging from -10.53 to 2.42 mm/h, indicating a significant influence of individual studies (**Figure 13**).

###### 3.1.3.2.6 White blood cell count

One study (27) (80 patients; low risk of bias), which used NSAIDs as controls, enrolled patients with UA <535 μmol/L, applied a 12-day treatment regimen with *Tufuling* 20 g/day combined with *Huangbo*, reported that comprehensive CHM therapy was superior to WM in reducing WBC count [MD:-1.24 (-2.07, -0.41)] (**Table 3**).

##### 3.1.3.3 CHM plus Western medicine treatment versus Western medicine (WM) treatment alone

###### 3.1.3.3.1 Visual analog scale

Combined therapy was superior to WM alone for pain reduction in 17 studies (15–17, 21, 28, 30–32, 41, 45, 47–51, 60, 70) (1351 patients) [MD: -0.94 (-1.20, -0.68), I^2^ = 93.2%]. Consistent treatment effects were observed across the prespecified subgroups (**Table 3**).

**Figure 14:**
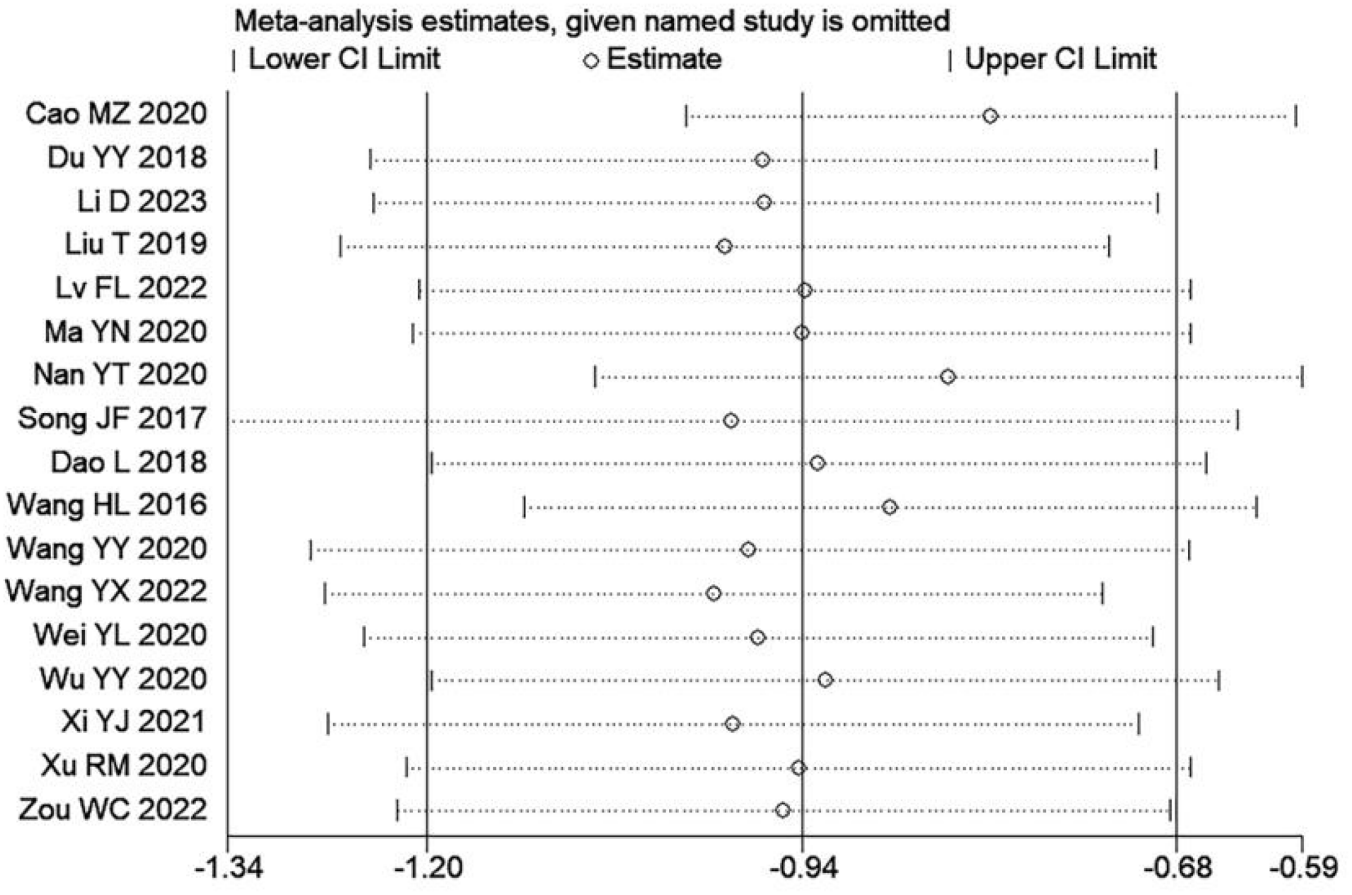
Results of the VAS sensitivity analysis in the CHM+WM group.

Meta-regression analysis revealed that none of the examined clinical or methodological covariates significantly modified the treatment effect on VAS (all *p* > 0.05) (**Table 4**). The sensitivity analysis confirmed high robustness, with the MD consistently between -1.34 and -0.59 and all CIs excluding zero (**Figure 14**).

**Table 4:** Univariable and multivariable meta-regression analyses for sources of heterogeneity across continuous outcomes in CHM+WM versus WM comparisons.

Covariate	VAS (n = 17)	UA (n= 33)	CRP (n= 30)	ESR (n = 30)	TCM syndrome score (n = 16)
Random sequence generation	−0.005 (−1.29, 1.28)	+14.03 (−10.87, 38.93)	+0.42 (−3.33, 4.18)	−1.01 (−4.86, 2.83)	— **^a^**
	*p* = 0.993; Adj *R*² = −9.74%	*p* = 0.259; Adj *R*² = 0.67%	*p* = 0.818; Adj *R*² = −4.69%	*p* = 0.594; Adj *R*² = −4.93%	
Treatment duration	+0.13 (−0.36, 0.62)	+2.73 (−6.45, 11.92)	+0.65 (−0.61, 1.92)	+0.08 (−1.19, 1.36)	−0.22 (−1.24, 0.80)
	*p* = 0.581; Adj *R*² = −7.39%	*p* = 0.548; Adj *R*² = −0.91%	*p* = 0.299; Adj *R*² = −1.13%	*p* = 0.893; Adj *R*² = −5.75%	*p* = 0.656; Adj *R*² = −5.61%
Control drug category	−0.22 (−2.28, 1.84)	+4.04 (−2.88, 10.95)	+0.56 (−0.23, 1.36)	+0.73 (−0.02, 1.48)	+0.89 (0.21, 1.56) **^b^**
	*p* = 0.823; Adj *R*² = −9.11%	*p* = 0.243; Adj *R*² = 3.53%	*p* = 0.157; Adj *R*² = 6.71%	*p* = 0.055 **^c^**; Adj *R*² = 17.86%	*p* = 0.014 **^b^**; Adj *R*² = 38.74%
Tufuling-Huangbo-Bixie-Niuxi combination	−0.71 (−1.61, 0.20)	+5.86 (−12.64, 24.35)	−0.84 (−3.39, 1.71)	+0.06 (−2.48, 2.61)	+0.56 (−1.36, 2.48)
	*p* = 0.117; Adj *R*² = 14.23%	*p* = 0.523; Adj *R*² = −2.67%	*p* = 0.504; Adj *R*² = −3.18%	*p* = 0.960; Adj *R*² = −5.15%	*p* = 0.539; Adj *R*² = −4.97%
Tufuling-Bixie	−0.47 (−1.52, 0.59)	−27.22 (−44.56, −9.88) **^b^**	−1.33 (−3.99, 1.33)	+0.48 (−2.22, 3.18)	−0.20 (−2.45, 2.06)
	*p* = 0.360; Adj *R*² = −1.26%	*p* = 0.003 **^b^**; Adj *R*² = 31.76%	*p* = 0.316; Adj *R*² = −1.49%	*p* = 0.721; Adj *R*² = −3.34%	*p* = 0.853; Adj *R*² = −7.96%
Tufuling-Huangbo	−0.79 (−1.84, 0.26)	−7.87 (−34.64, 18.90)	−0.01 (−3.74, 3.72)	+2.75 (−0.51, 6.00) **^c^**	+1.79 (−0.48, 4.05) **^c^**
	*p* = 0.128; Adj *R*² = 14.48%	*p* = 0.553; Adj *R*² = −1.69%	*p* = 0.995; Adj *R*² = −6.07%	*p* = 0.095 **^c^**; Adj *R*² = 20.53%	*p* = 0.113; Adj *R*² = 17.47%
Dose of Tufuling	+0.12 (−0.68, 0.92)	+3.07 (−12.26, 18.40)	+0.30 (−1.71, 2.30)	−0.47 (−2.44, 1.50)	−1.12 (−3.19, 0.96)
	*p* = 0.751; Adj *R*² = −7.74%	*p* = 0.685; Adj *R*² = −3.34%	*p* = 0.762; Adj *R*² = −4.82%	*p* = 0.629; Adj *R*² = −2.15%	*p* = 0.269; Adj *R*² = 7.51%
Baseline uric acid level	−0.02 (−0.86, 0.83)	+9.77 (−4.24, 23.79)	+0.65 (−1.27, 2.58)	−0.04 (−1.95, 1.87)	+0.86 (−1.07, 2.79)
	*p* = 0.970; Adj *R*² = −9.36%	*p* = 0.165; Adj *R*² = 6.90%	*p* = 0.493; Adj *R*² = −4.81%	*p* = 0.965; Adj *R*² = −6.08%	*p* = 0.357; Adj *R*² = −6.20%
Multivariable model (UA) ^d^	—	See below ^d^	—	—	—
Multivariable model (ESR) ^e^	—	—	—	See below ^e^	—

All meta-regressions were performed using the REML method with Knapp–Hartung modification. Data are presented as regression coefficient (95% CI). n, number of studies included in the meta-regression.

^a^ Automatically dropped due to collinearity (all 16 studies reporting this outcome were rated as low risk for random sequence generation).

^b^ Statistically significant (*p* < 0.05).

^c^ Trend-level significance (0.05 ≤ *p* < 0.10).

^d^ Multivariable model for UA (*Bixie* + nualevel): *Bixie* = −29.30 (−45.35 to −13.25), *P* = 0.001; nualevel = +12.81 (1.14 to 24.47), *P* = 0.032; model F(2,30) = 8.68, *P* = 0.0011, Adj R² = 46.05%.

^e^ Multivariable model for ESR (ncontrole + *Huangbo*): ncontrole = +0.54 (−0.20 to 1.27), *P* = 0.145; *Huangbo* = +2.17 (−1.10 to 5.43), *P* = 0.185; model F(2,26) = 2.80, *P* = 0.0793, Adj R² = 29.10%.

###### 3.1.3.3.2 Effective rate

**Figure 15:**
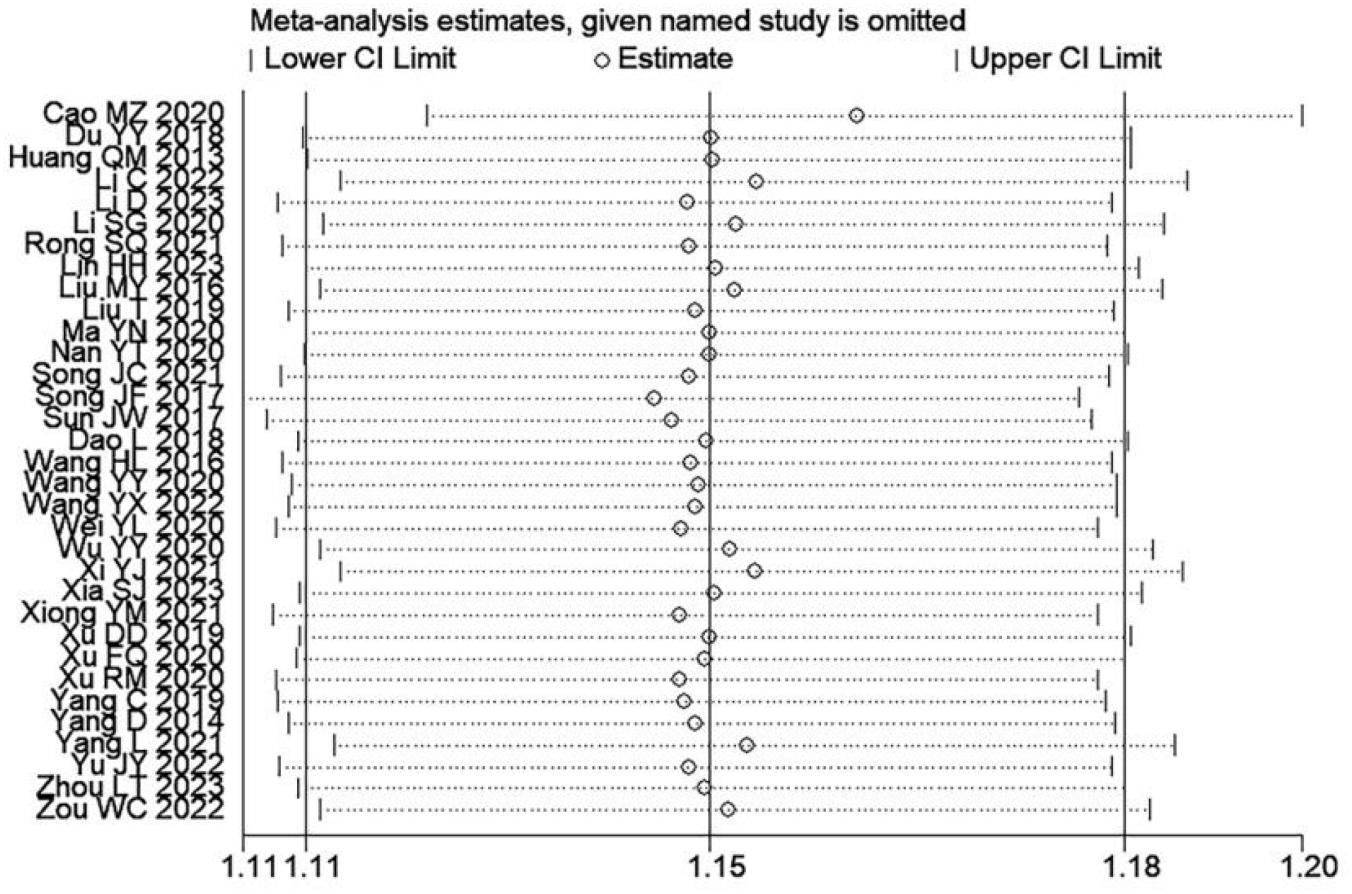
Results of the effective rate sensitivity analysis in the CHM+WM group.

Based on a pooled analysis of 33 studies (15–18, 20, 21, 23, 24, 26, 28, 31, 32, 38, 40–42, 45, 47–52, 57–64, 68, 70) (N = 2,597 patients), combined therapy demonstrated a significantly higher effective rate than Western medicine (WM) alone [RR: 1.15 95% CI (1.11, 1.19); I²=0.0%]. In the prespecified subgroup analyses, the combined therapy had a superior effect across most subgroups. However, no statistically significant advantage was found in the subgroups where the control regimen consisted of NSAIDs and allopurinol (50), NSAIDs, colchicine and allopurinol (58), or NSAIDs and benzbromarone (68) (**Table 3**). The sensitivity analysis indicated exceptional robustness, with RR estimates tightly clustered between 1.11 and 1.20, and all CIs having a lower limit >1 (**Figure 15**).

###### 3.1.3.3.3 Uric acid

Combined therapy was superior to WM alone for UA reduction according to 33 studies (15–18, 20, 21, 23, 24, 26, 28, 30–32, 38, 40–42, 45, 47–52, 57, 58, 60–64, 68, 70) (2597 patients) [MD: -57.02(-66.31, -47.73), I^2^ = 86.9%], except in the subgroup where the control was NSAIDs+benzbromarone (68) (**Table 3**).

Meta-regression analysis identified the presence of *Bixie* as the only significant effect modifier. Studies containing *Bixie* demonstrated a substantially greater reduction in UA compared with those without (coefficient = −27.22, 95% CI: −44.56 to −9.88, *P* = 0.003), with this variable explaining approximately 31.76% of the between-study heterogeneity (residual τ² = 320.4, I² = 79.85%). None of the other examined covariates showed a statistically significant association with effect size (all *P* > 0.16) (**Table 4**).

To further elucidate the independent contributions of herbal composition and patient baseline status, a multivariable meta-regression was performed for the UA outcome. The model including both *Bixie* and baseline UA level was statistically significant (F(2,30) = 8.68, *P* = 0.0011) and explained 46.05% of between-study variance (residual τ² = 253.3, I² = 76.49%). After adjusting for baseline uric acid level, the presence of *Bixie* remained strongly associated with greater UA reduction (coefficient = −29.30, 95% CI: −45.35 to −13.25, *P* = 0.001). Notably, baseline UA level emerged as a significant independent effect modifier in the multivariable model (coefficient = +12.81, 95% CI: 1.14 to 24.47, *P* = 0.032), whereas it was non-significant in univariable analysis (*P* = 0.165) (**Table 4**).

**Figure 16:**
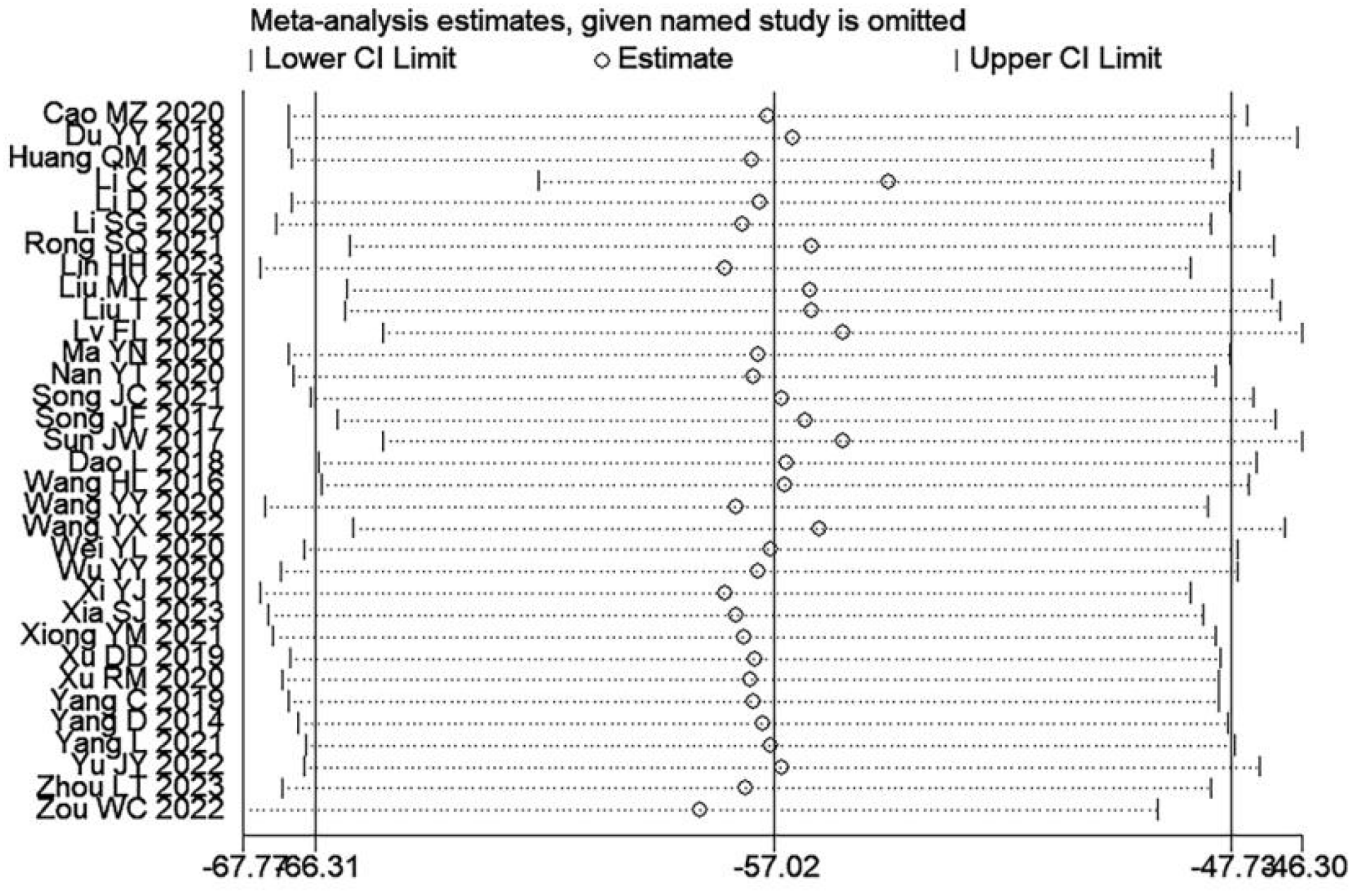
Results of the UA sensitivity analysis in the CHM+WM group.

The findings were highly robust, with sensitivity analysis yielding MD values between -67.77 and -46.30 μmol/L and CIs far from zero (**Figure 16**).

###### 3.1.3.3.4 C-reactive protein

**Figure 17:**
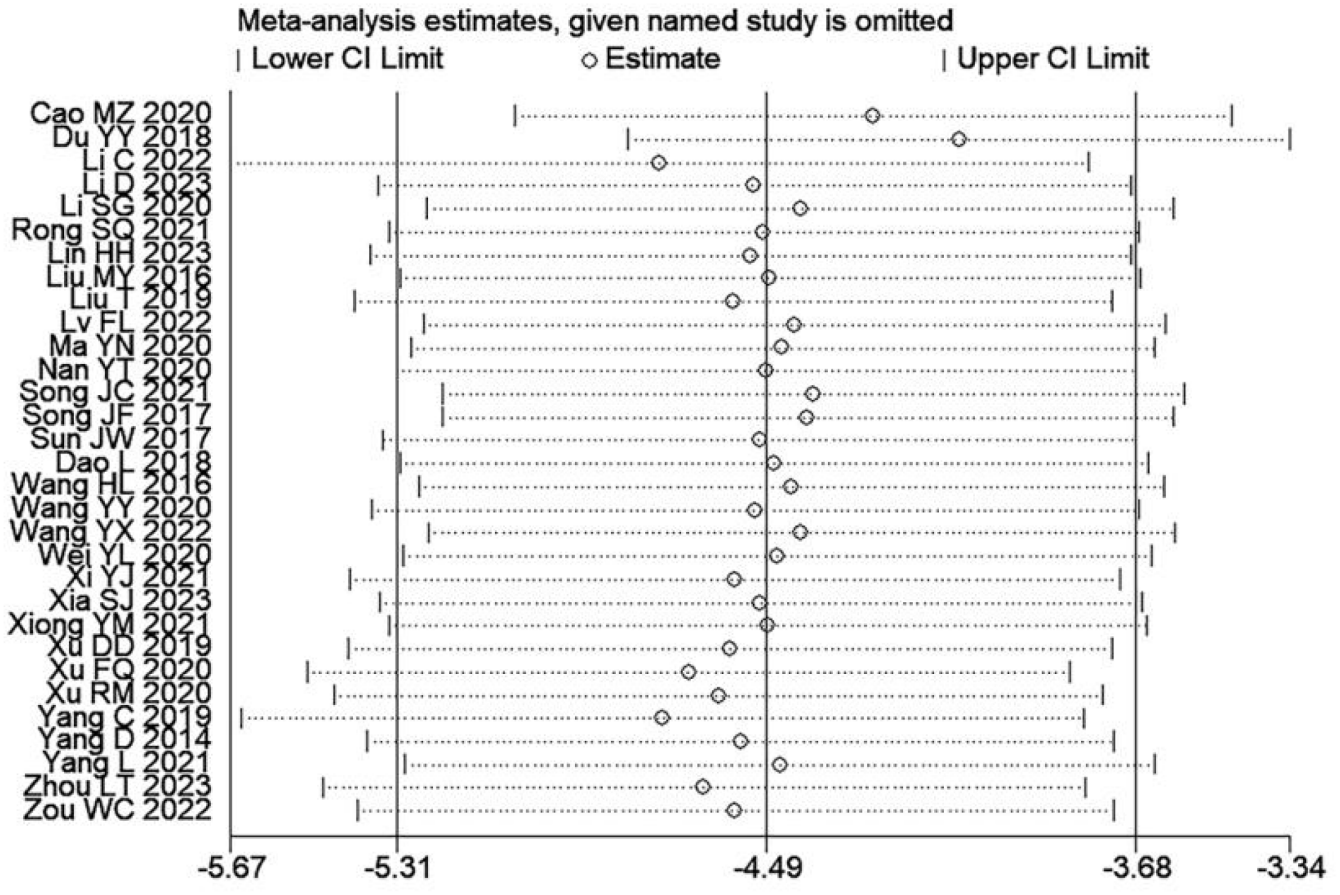
Results of the CRP sensitivity analysis in the CHM+WM group.

Combined therapy was superior to WM alone for CRP reduction in 31 studies (15–17, 20, 21, 23, 24, 26, 28, 30–32, 38, 40–42, 45, 47–49, 51, 52, 57–63, 68, 70) (2418 patients) [MD: -4.49 (-5.31, -3.68), I^2^ = 87.9%] except in the NSAIDs+benzbromarone control subgroup (68) (**Table 3**). Meta-regression analysis revealed that none of the prespecified covariates significantly modified the treatment effect on CRP (all *P* > 0.15) (**Table 4**). The result was highly robust, with recalculated MDs narrowly ranging from -5.67 to -3.34 mg/L and all CIs excluding zero (**Figure 17**).

###### 3.1.3.3.5 Erythrocyte sedimentation rate

Combined therapy was superior to WM alone for ESR reduction in 31 studies (15–17, 20, 21, 23, 24, 26, 28, 30–32, 38, 40–42, 45, 47–49, 51, 52, 57–63, 68, 70) (2418 patients) [MD: -6.06(-7.22, -4.90), I^2^ = 83.2%]. The results were consistent across the prespecified subgroup analyses (**Table 3**).

Meta-regression analysis revealed that none of the examined covariates reached conventional statistical significance (all *P* > 0.05). However, two variables demonstrated borderline associations. The presence of *Huangbo* was associated with a smaller ESR reduction (coefficient = +2.75, 95% CI: −0.51 to 6.00, *P* = 0.095), explaining approximately 20.53% of between-study variance (residual τ² = 5.40, I² = 69.92%). Similarly, more complex control drug regimens showed a trend toward attenuated incremental ESR reduction (coefficient = +0.73, 95% CI: −0.02 to 1.48, *P* = 0.055, adjusted R² = 17.86%) (**Table 4**).

Multivariable meta-regression for ESR, including both control drug category and presence of *Huangbo*, yielded a marginally significant model (F(2,26) = 2.80, *p* = 0.079). The combined covariates explained 29.10% of between-study variance, reducing residual heterogeneity to a moderate-to-high level (τ²= 4.82, I²= 67.61%). However, after mutual adjustment, neither control drug category (coefficient = +0.54, 95% CI: −0.20 to 1.27, *P* = 0.145) nor *Huangbo* (coefficient = +2.17, 95% CI: −1.10 to 5.43, *P* = 0.185) retained individual statistical significance (**Table 4**).

**Figure 18:**
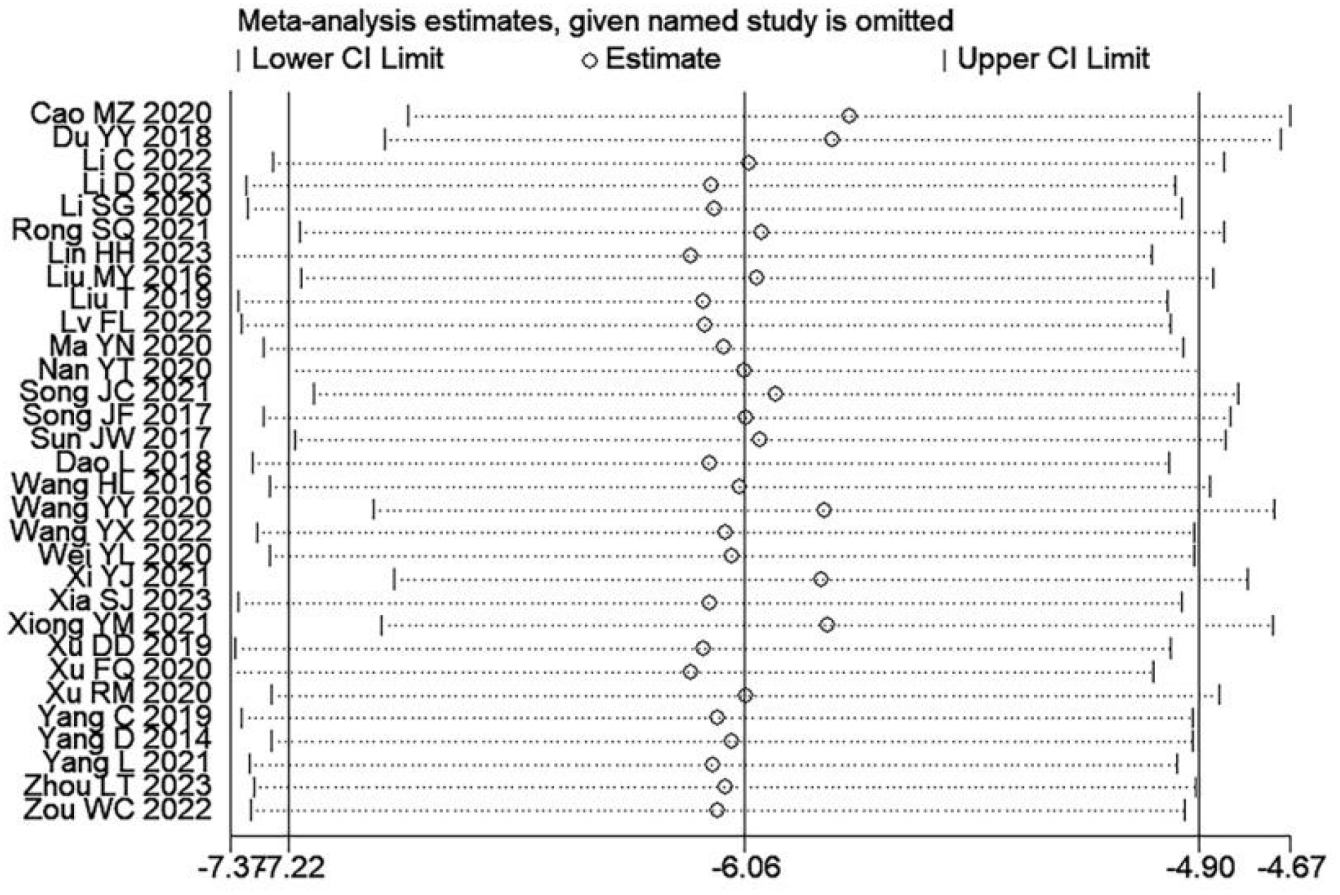
Results of the ESR sensitivity analysis in the CHM+WM group.

Sensitivity analysis demonstrated robustness, with MD estimates varying from -7.37 to -4.67 mm/h and all CIs remaining below zero (**Figure 18**).

###### 3.1.3.3.6 White blood cell count

**Figure 19:**
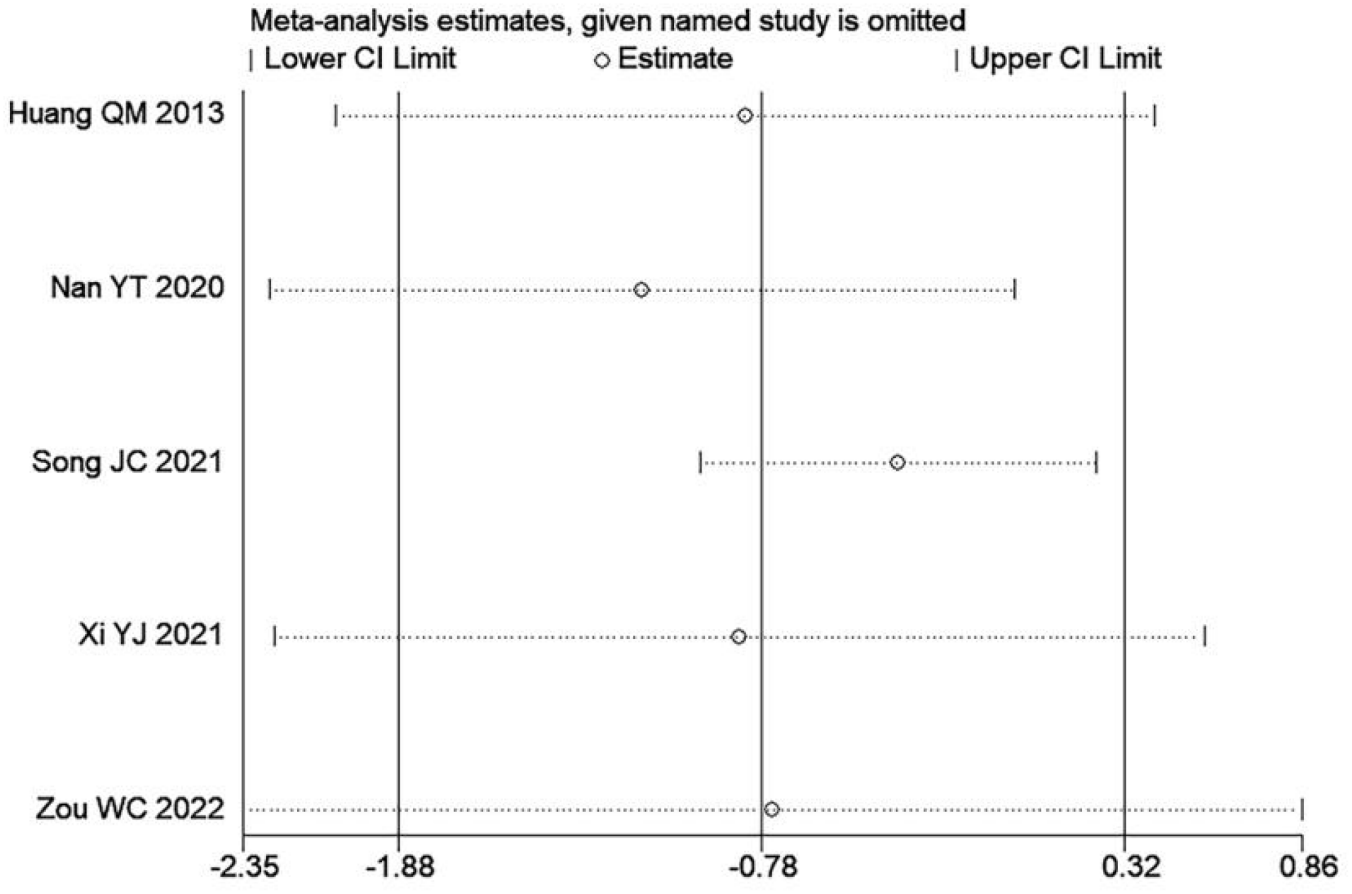
Results of the WBC sensitivity analysis in the CHM+WM group.

An analysis of five studies (18, 32, 40, 51, 70) (342 patients) revealed no significant difference between combined therapy and WM alone for WBC reduction [MD: -0.78(-1.88, 0.32), I^2^ = 89.9%]. A consistent pattern of statistical effects was observed in the prespecified subgroup analyses (**Table 3**). This finding lacked robustness, as the sensitivity analysis revealed MDs ranging from -2.35 to 0.86 x10^9^/L, with CIs frequently including zero (**Figure 19**).

###### 3.1.3.3.7 TCM syndrome scoring

**Figure 20:**
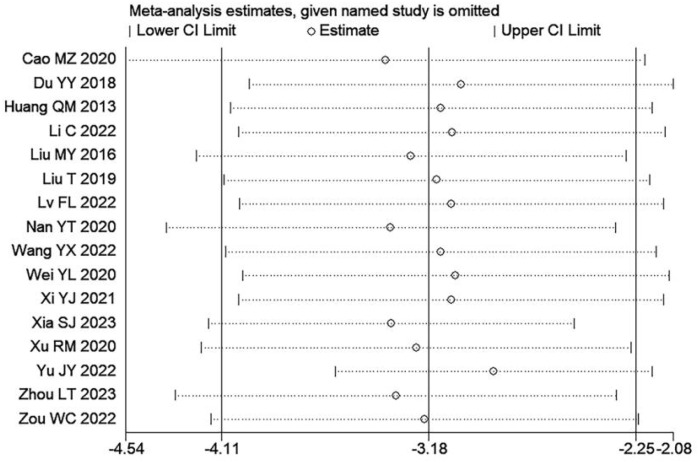
Results of the TCM Syndrome Scoring sensitivity analysis in the CHM+WM group.

Combined therapy was superior to WM alone for improving TCM syndrome scores in 16 studies (15, 17, 18, 20, 26, 28, 30, 32, 47, 49, 51, 52, 60, 64, 68, 70) (1242 patients) [MD: -3.18(-4.11, -2.25), I^2^ = 98.1%], except in the NSAIDs+colchicine control subgroup [MD: -0.960[-2.530, 0.611], I^2^ = 71.3%] (26, 52) (**Table 3**). Meta-regression analysis identified control drug regimens as the only significant effect modifier for TCM syndrome score improvement (coefficient = +0.89, 95% CI: 0.21 to 1.56, *P* = 0.014), explaining 38.74% of between-study heterogeneity (residual τ² = 1.41). More complex western medicine control protocols (e.g., NSAIDs combined with colchicine or urate-lowering agents) were associated with a smaller incremental TCM syndrome score reduction attributable to Chinese herbal medicine. The presence of Phellodendron chinense (*Huangbo*) showed a non-significant but directionally consistent trend toward attenuated efficacy (coefficient = +1.79, 95% CI: −0.48 to 4.05, *P* = 0.113, adjusted R² = 17.47%), corroborating the pattern observed in the ESR analysis. Treatment duration, *Bixie*, *Tufuling*-*Huangbo*-*Bixie*-*Niuxi*, and baseline uric acid level showed no significant association (all *P* > 0.35) (**Table 4**). The result was robust, with MDs stable between -4.54 and -2.25, and all CIs excluding zero (**Figure 20**).

##### 3.1.3.4 CHM comprehensive therapy plus Western medicine treatment (COM+WM) versus Western medicine treatment

###### 3.1.3.4.1 Visual analog scale

**Figure 21:**
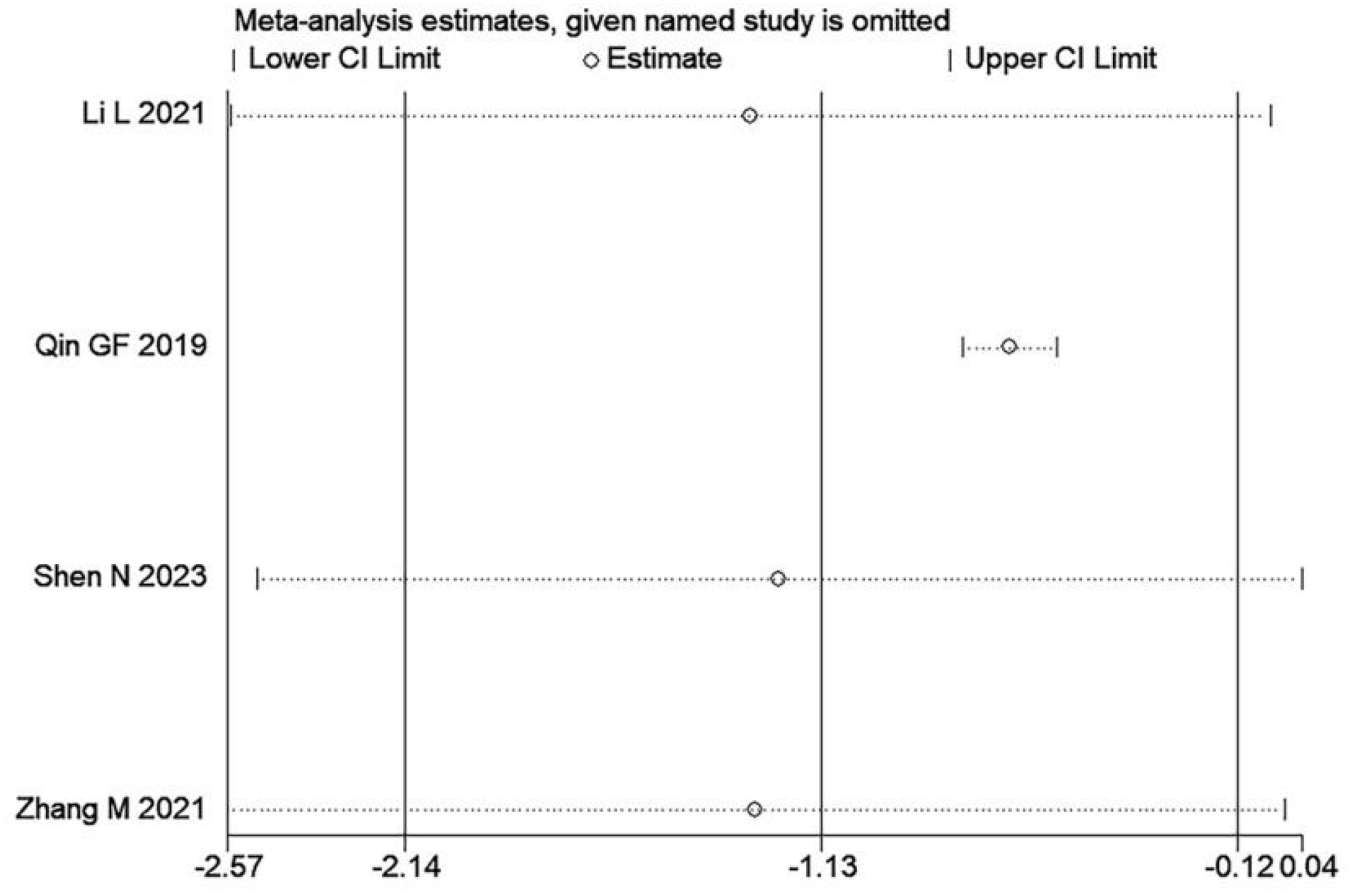
Results of the VAS sensitivity analysis in the COM+WM group.

The combination was superior to WM alone in four studies (22, 37, 39, 67) (413 patients) [MD: -1.13(-2.14, -0.12), I^2^ = 99.2%]; however, this superiority was not observed in the NSAIDs-controlled (37, 67), *Tufuling* 30-45g/day (22), *Tufuling*-*Bixie (*22, 37, 67), or *Tufuling-Huangbo-Bixie-Niuxi (*22, 37, 67) subgroups. (**Table 3**). The sensitivity analysis indicated good robustness, with predominantly negative MDs (-2.57 to 0.04) (**Figure 21**).

###### 3.1.3.4.2 Effective rate

**Figure 22:**
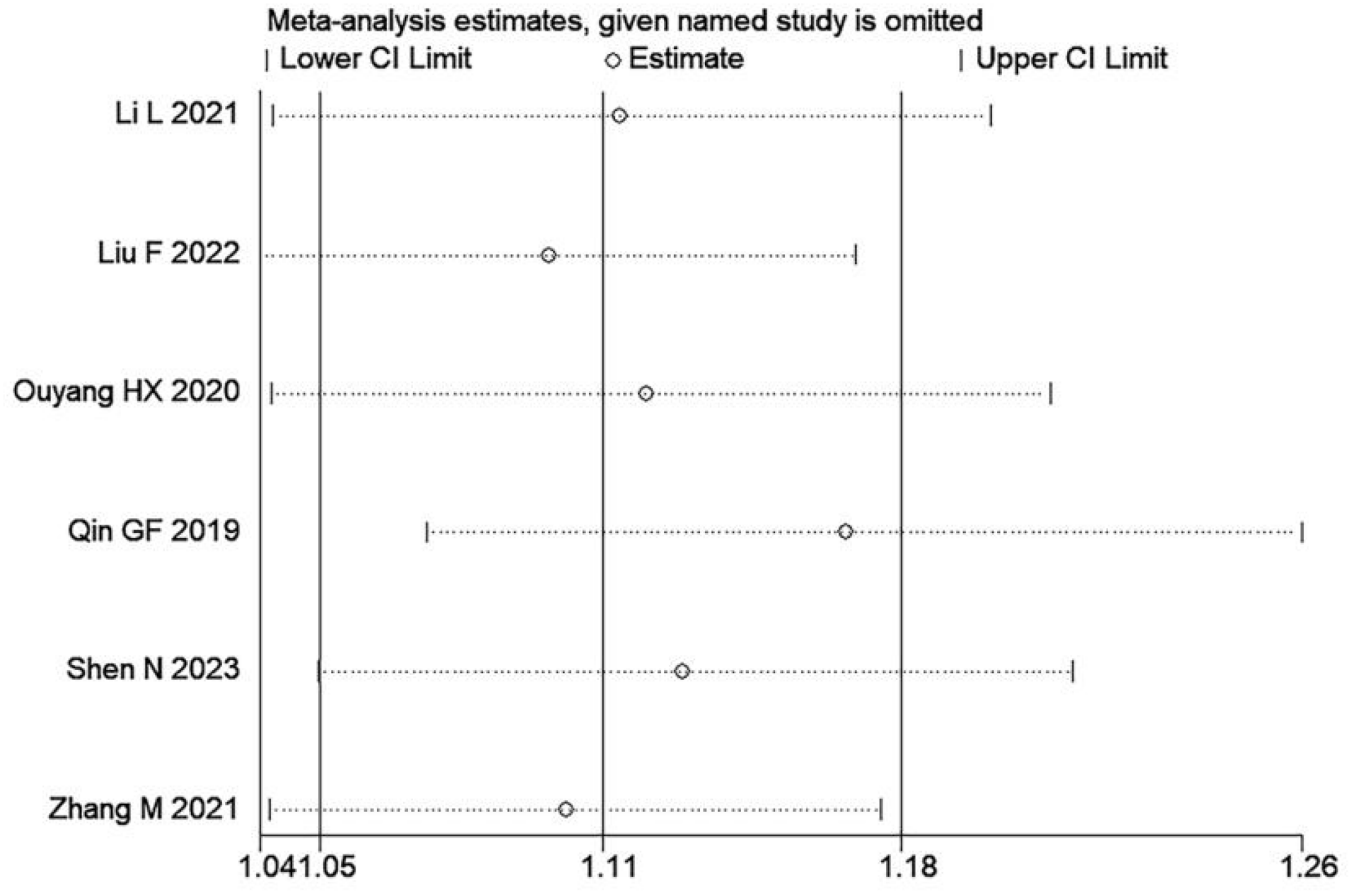
Results of the effective rate sensitivity analysis in the COM+WM group.

A pooled analysis of six studies (22, 25, 37, 39, 67, 71) (N = 580 patients) demonstrated that combined therapy (TCM comprehensive therapy plus WM) had a significantly greater effective rate than WM alone [RR: 1.11, 95% CI (1.05, 1.18); I²=0.0%]. In the prespecified subgroup analyses, the combined therapy had a superior effect in most subgroups. However, no statistically significant advantage was found in the following specific subgroups: trials in which the control intervention was colchicine alone (22), a combination of NSAIDs and colchicine (39), participants with baseline serum uric acid levels between 535–590 μmol/L (37), and treatment duration 7-14days (71) or ≥14 days (22) (**Table 3**). The sensitivity analysis confirmed very high robustness, with all recalculated RRs >1 (range: 1.04–1.26) (**Figure 22**).

###### 3.1.3.4.3 Uric acid

**Figure 23:**
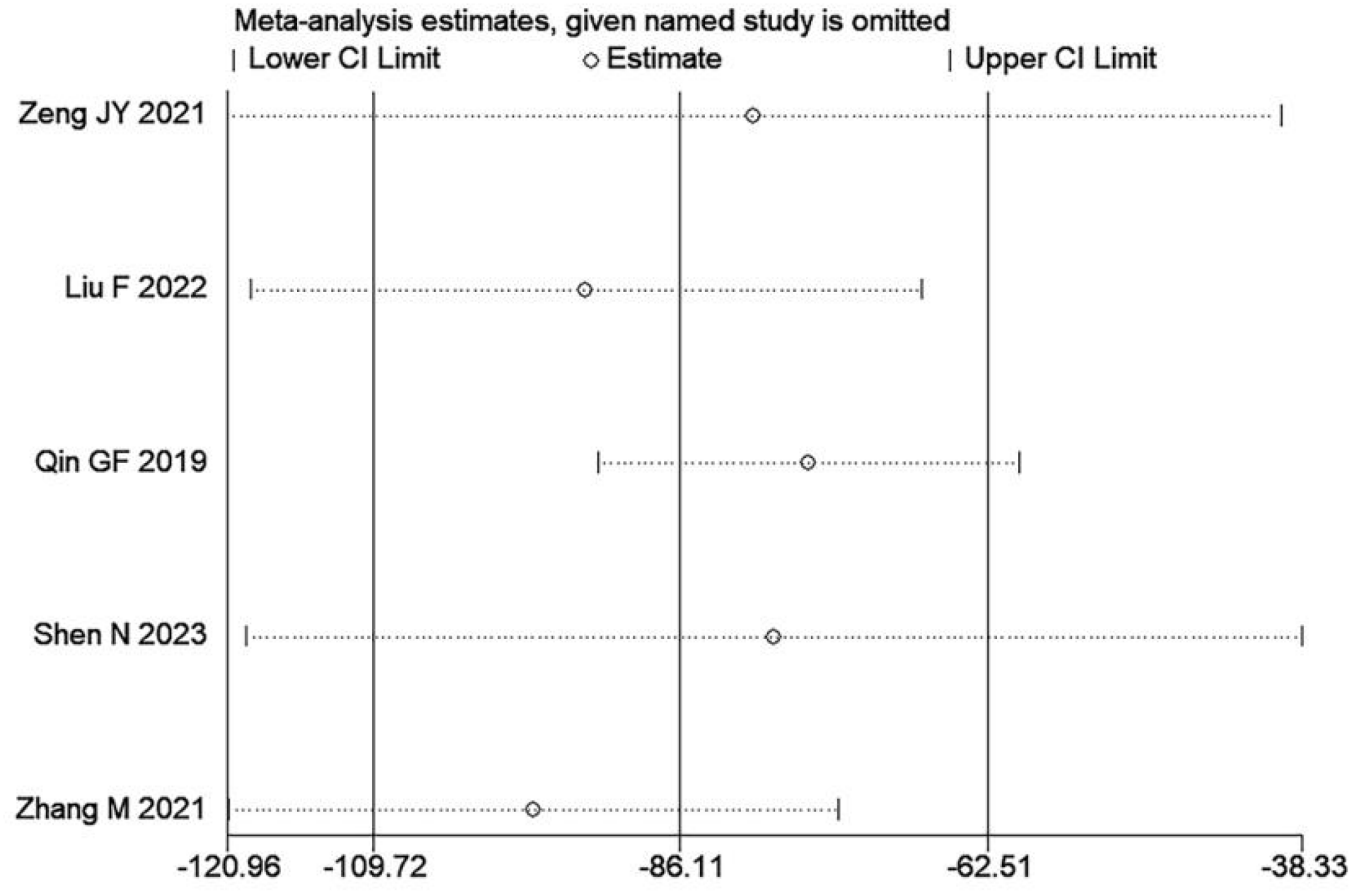
Results of the UA sensitivity analysis in the COM+WM group.

The combination was superior to WM alone for UA reduction according to five studies (25, 37, 39, 65, 67) (457 patients) [MD: -86.11(-109.72, -62.51), I^2^ = 95.3%] with the exception of the NSAIDs control subgroup (37, 67) (**Table 3**). The direction of the effect was consistent, but the precision of the point estimate had low robustness, varying greatly from -120.96 to -38.33 μmol/L in the sensitivity analysis, indicating high sensitivity to specific studies (**Figure 23**).

###### 3.1.3.4.4 C-reactive protein

**Figure 24:**
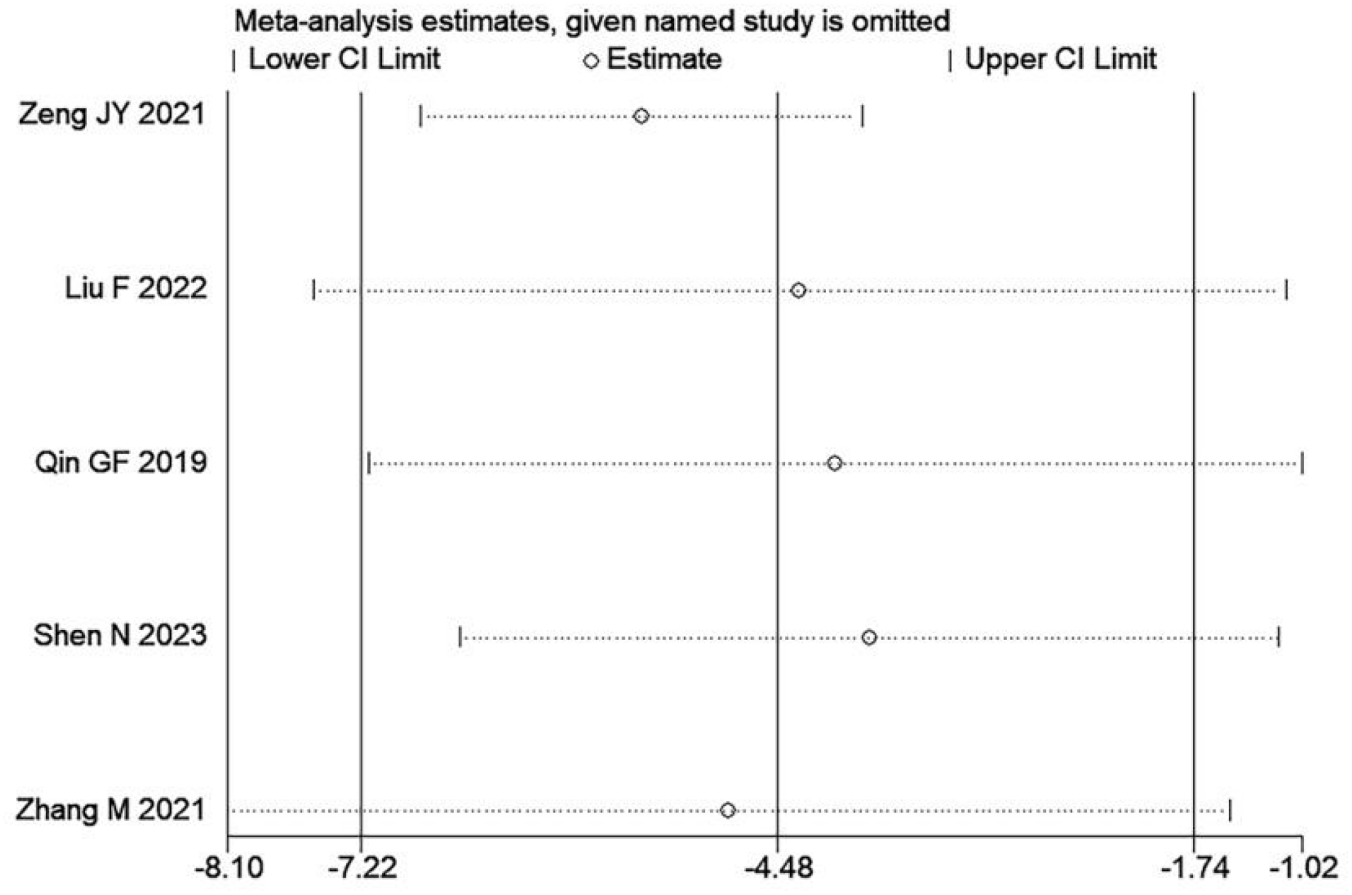
Results of the CRP sensitivity analysis in the COM+WM group.

The combination was superior to WM alone for CRP reduction according to five studies (25, 37, 39, 65, 67) (457 patients) [MD: -4.48(-7.22, -1.74), I^2^ = 97.7%], except in the NSAIDs+colchicine control subgroup (39, 65) (**Table 3**). The results showed high robustness, with MDs varying narrowly from -8.10 to -1.02 mg/L upon exclusion of the study (**Figure 24**).

###### 3.1.3.4.5 Erythrocyte sedimentation rate

**Figure 25:**
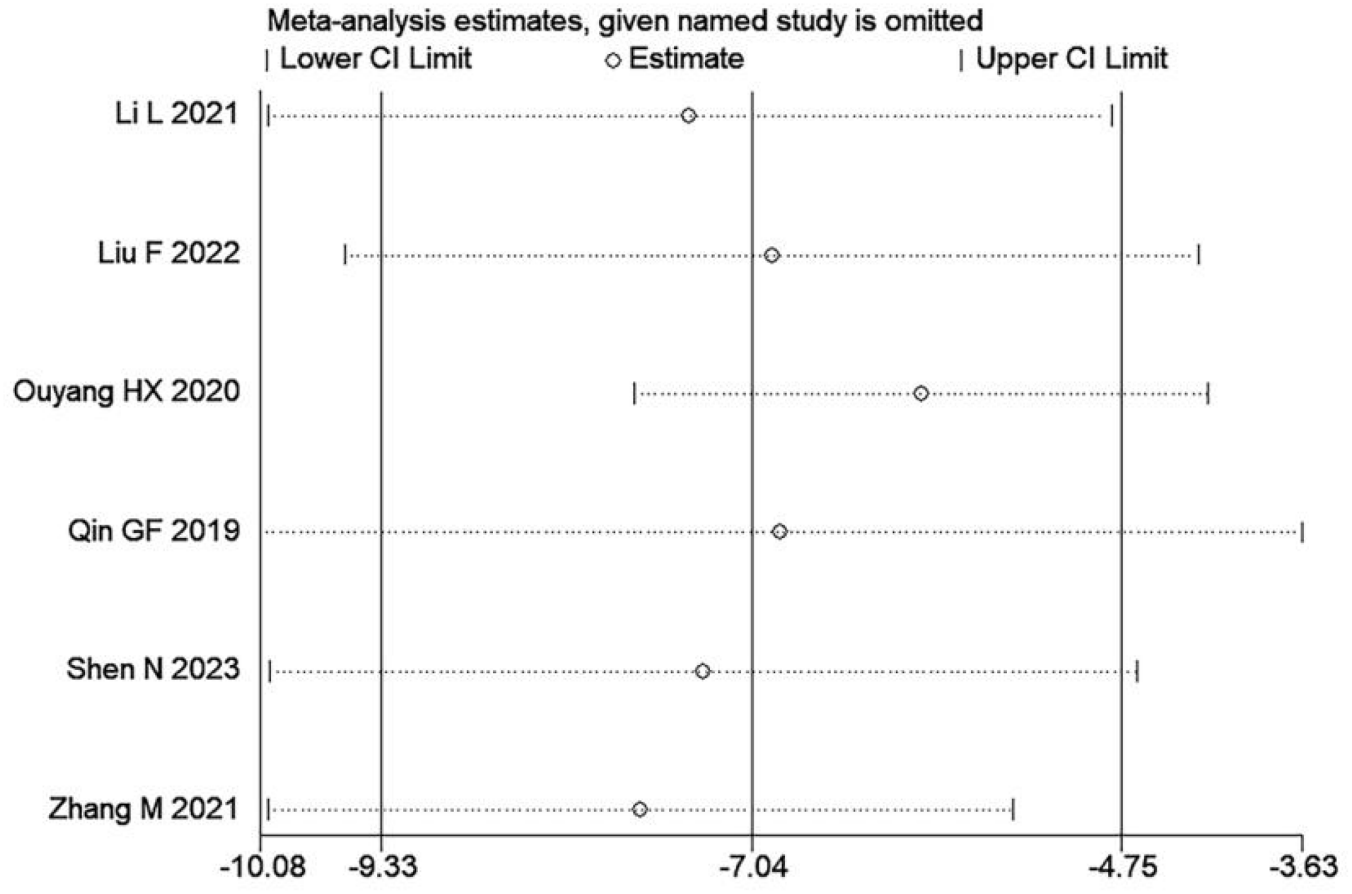
Results of the ESR sensitivity analysis in the COM+WM group.

The combination was superior to WM alone for ESR reduction based on six studies (22, 25, 37, 39, 67, 71) (580 patients) [MD: -7.04(-9.33, -4.75), I^2^ = 93.7%]. The results were consistent across the prespecified subgroup analyses (**Table 3**). The robustness was moderate, as the sensitivity analysis revealed MD estimates between -10.08 and -3.63 mm/h, indicating the influence of individual studies (**Figure 25**).

###### 3.1.3.4.6 TCM syndrome scoring

**Figure 26:**
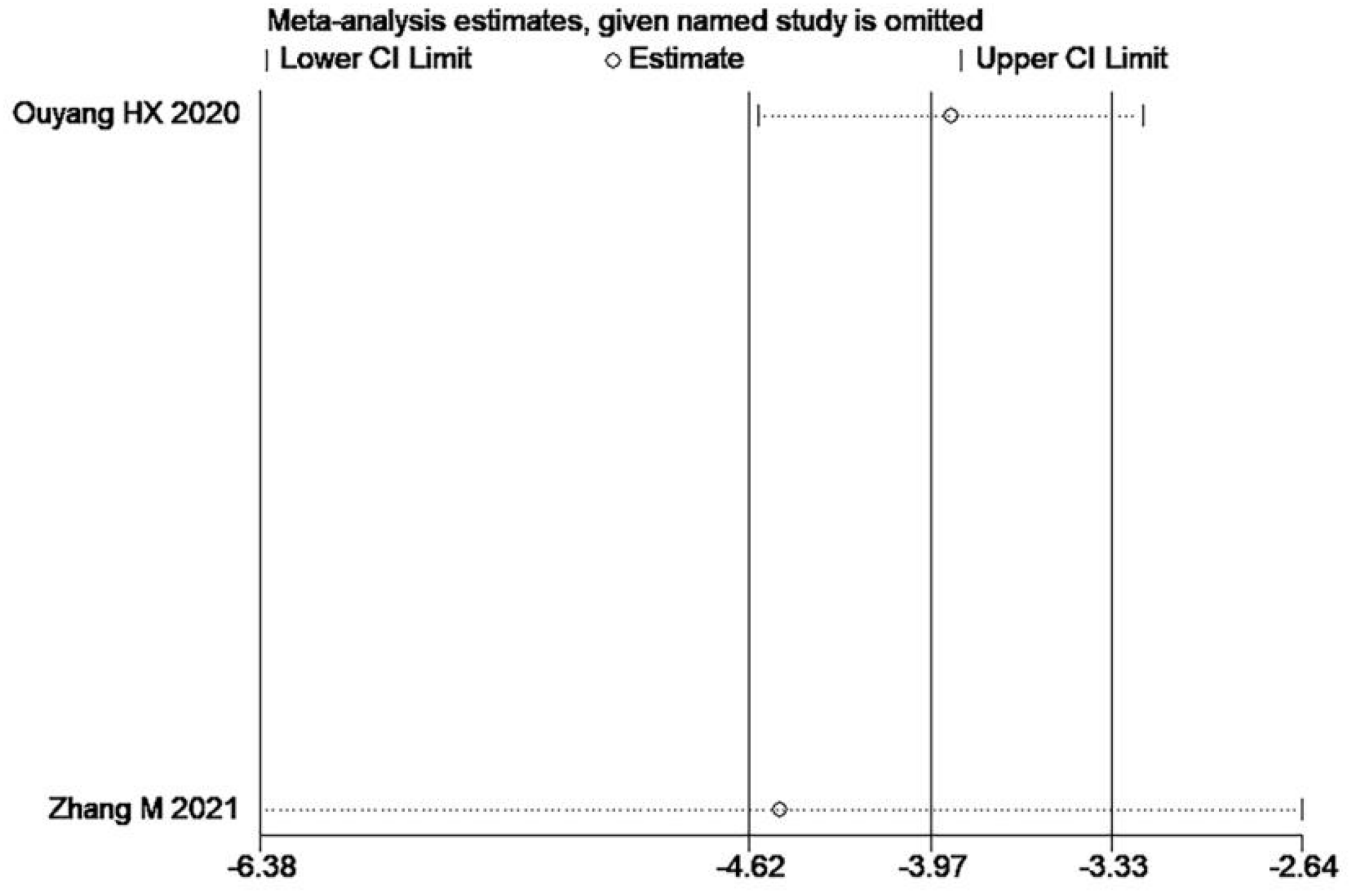
Results of the TCM Syndrome Scoring sensitivity analysis in the COM+WM group.

Two low-risk-of-bias studies (67, 71) (147 patients) using NSAIDs as controls and enrolling patients with baseline UA of 535-590 μmol/L demonstrated the superiority of combination therapy over WM alone for TCM syndrome score improvement [MD -3.97 (-4.62, -3.33), I²=0.0%]. Both studies administered a 7- or 10-day course of *Tufuling* at 30 g/day, with one study (67) combining *Tufuling* with *Huangbo*, *Bixie*, and *Niuxi (*67). (**Table 3**). The findings were robust, with MDs ranging from -6.38 to -2.64 (**Figure 26**).

#### 3.1.4 Adverse events

Among the 56 studies, 42 reported on AEs (16–23, 25, 27–30, 32–37, 39, 40, 42, 43, 45–52, 54, 55, 59–63, 66–68, 71), with 36 providing specific details (16–20, 22, 23, 27–30, 33–36, 40, 42, 43, 46–52, 54, 55, 59–63, 66–68, 71) and 6 documented zero AEs (21, 25, 32, 37, 39, 45). And 14 did not mention AEs (treated as missing data and excluded from quantitative synthesis).

Among the seven studies (33–36, 43, 46, 55) reporting adverse events in the Chinese herbal medicine (CHM) group versus the Western medicine (WM) group, nine cases of gastrointestinal symptoms were documented in the CHM group. In contrast, the WM group reported 59 cases of gastrointestinal adverse effects, and one case of hepatorenal dysfunction. Pooled analysis revealed a significantly lower AE risk in the CHM group [RR 0.25, 95% CI (0.13, 0.48), *P* < 0.001; Peto OR 0.23, 95% CI (0.14, 0.38)], with no heterogeneity [I² = 0%]. The absolute risk difference was –0.12 [95% CI (–0.20, –0.05)], corresponding to an NNT of 8 [95% CI (5, 21)] (**Table 5**).

**Table 5:** Meta-analysis of adverse events across the four prespecified comparisons.

Group	Studies (n)	Intervention Events / N	Control Events / N	RR [95% CI]	Peto OR [95% CI]	RD [95% CI]	NNT [95% CI]	I²	*P*
CHM vs WM	7	9 / 329	60 / 329	0.25 [0.13, 0.48]	0.23 [0.14, 0.38]	-0.12 [-0.20, -0.05]	8 [5, 21]	0.0%	0.00
CHM+WM vs WM	24	41 / 952	86 / 914	0.44 [0.33, 0.59]	0.40 [0.31, 0.53]	-0.05 [-0.07, -0.03]	19 [14, 31]	0.0%	0.00
COM vs WM	5	12 / 190	30 / 190	0.53 [0.27, 1.04]	0.42 [0.22, 0.81]	-0.07 [-0.13, -0.01]	14[8,111]	3.1%	0.066
COM+WM vs WM	6	11 / 291	11 / 289	1.00 [0.44, 2.26]	0.99 [0.42, 2.35]	-0.00 [-0.02, 0.02]	—	0.0%	0.994

AE, adverse event; CHM, Chinese herbal medicine; COM, comprehensive TCM therapy; WM, Western medicine; RR, relative risk; CI, confidence interval; OR, odds ratio; RD, risk difference; NNT, number needed to treat. Zero-event studies were included with a continuity correction of 0.5. All reported AEs were mild to moderate; no severe AEs were observed.

Among the 24 studies (16–18, 20, 21, 23, 28, 30, 32, 40, 42, 45, 47–52, 59–63, 68) comparing adverse events in the Chinese herbal medicine combined with Western medicine (CHM+WM) and Western medicine (WM) alone groups, three studies (21, 32, 45) reported the absence of AEs. The remaining 21 studies (16–18, 20, 23, 28, 30, 40, 42, 47–52, 59–63, 68) provided specific details, indicating that all documented AEs were gastrointestinal. Specifically, 41 cases were reported in the intervention (CHM+WM) group, whereas 86 cases were reported in the control (WM alone) group, including three cases presenting with concurrent rash. Pooled analysis demonstrated a significantly lower AE risk with CHM+WM [RR 0.44, 95% CI (0.33, 0.59), *P* < 0.001; Peto OR 0.40, 95% CI (0.31, 0.53)], with no heterogeneity [I² = 0%]. The RD was –0.05 [95% CI (–0.07, –0.03)], corresponding to an NNT of 19 [95% CI (14, 31)] (**Table 5**).

Among the five studies (19, 27, 29, 54, 66) reporting adverse events in the Comprehensive Chinese Medicine (COM) versus Western Medicine (WM) group, the intervention (COM) group reported 12 cases of AEs (including three cases of rash), whereas the control (WM) group reported 30 cases (with six cases of rash). Meta-analysis indicated a trend toward reduced AE risk in the COM group [RR 0.53, 95% CI (0.27, 1.04), *P* = 0.066; Peto OR 0.42, 95% CI (0.22, 0.81), *P* = 0.009], with minimal heterogeneity [I² = 3.1%]. The RD was –0.07 [95% CI (–0.13, –0.01)], with an NNT of 14 [95% CI (8, 111)] (**Table 5**).

Among the six studies (22, 25, 37, 39, 67, 71) reporting adverse events in the COM combined with WM (COM+WM) group versus the WM alone group, three studies (25, 37, 39) reported no AEs. The remaining three studies (22, 67, 71) provided specific details: the intervention (COM+WM) group reported 11 cases (including two cases of rash), and the control (WM alone) group reported 11 cases (with one case of rash). No significant between-group difference was observed [RR 1.00, 95% CI (0.44, 2.26), *P* = 0.994; Peto OR 0.99, 95% CI (0.42, 2.35)], with no heterogeneity [I² = 0%]. The RD was approximately zero [–0.00, 95% CI (–0.02, 0.02)] (**Table 5**).

Summary of adverse events: a total of 73 adverse events were reported in the treatment groups, of which 68 were gastrointestinal adverse reactions and 5 were cutaneous rashes. The control group reported 161 adverse events, comprising 151 gastrointestinal adverse reactions and 10 cases of rash. All AEs were mild to moderate; no severe AEs were observed in any included study.

#### 3.1.5 Assessment via GRADE

The quality of evidence from the RCTs was assessed using GRADE. The interventions, comparators, and outcomes included were selected based on a consensus process. Comparisons were as follows: CHM versus Western medicine treatment, CHM comprehensive therapy versus Western medicine treatment, CHM plus Western medicine treatment versus Western medicine treatment, and CHM comprehensive therapy plus Western medicine treatment versus Western medicine treatment.

The evidence for *Tufuling*-containing formulae in gout ranged from low to moderate certainty (**Table 6**). The results showed that oral formulations containing *Tufuling* may ameliorate pain symptoms, inflammation, and high serum uric acid levels. Meta-analysis suggested a lower incidence of reported adverse events in the treatment groups; however, this observation was limited by incomplete reporting and short treatment durations.

**Table 6:** GRADE quality of the evidence of the Tufuling*-Containing* formula for gout.

Outcomes	№ of participants (studies) Follow-up	Certainty of the evidence (GRADE)	Relative effect (95% CI)	Anticipated absolute effects	
*CHM* vs*. Western medicine treatment*				Risk with WM	Risk difference with CHM
Visual Analog Scale (VAS) Scale from: 1 to 10	108 (2 RCTs)	⊕⊕◯◯ Low^b,c,d^	–	The mean visual Analog Scale was **3.61**	MD **0.33 lower** (0.88 lower to 0.23 higher)
Effective rate (Effective rate)	658 (7 RCTs)	⊕⊕◯◯ Low^b,c,d^	**RR 1.06** (1.01 to 1.11)	881 per 1,000	**53 more per 1,000** (9 more to 97 more)
Uric Acid (UA)	568 (6 RCTs)	⊕⊕◯◯ Low^a,c,d^	–	The mean uric Acid was **423.03** μmol/L	MD **47.56 μmol/L lower** (68.92 lower to 26.21 lower)
C-Reactive Protein (CRP)	618 (6 RCTs)	⊕⊕◯◯ Low^a,c,d^	–	The mean c-Reactive Protein was **16.62** mg/L	MD **2.75 mg/L lower** (4.21 lower to 1.29 lower)
Erythrocyte Sedimentation Rate (ESR)	440 (4 RCTs)	⊕⊕◯◯ Low^a,c,d^	–	The mean erythrocyte Sedimentation Rate was **32.70** mm/h	MD **5.76 mm/h lower** (11.02 lower to 0.5 lower)
White Blood Cell Count (WBC)	40 (1 RCT)	⊕⊕◯◯ Low^d,e^	–	The mean white Blood Cell Count was **8.99** ×10^9^/L	MD **2** ×**10^9^/L lower** (3.21 lower to 0.79 lower)
TCM Syndrome Scoring	58 (1 RCT)	⊕◯◯◯ Very low^b,d,e^	–	The mean TCM Syndrome Scoring was **7.38**	MD **0** (2.2 lower to 2.2 higher)
Adverse events (AEs)	658 (7 RCTs)	⊕⊕◯◯ Low^a,c,d^	**RR 0.25** (0.13 to 0.48)	182 per 1,000	**137 fewer per 1,000** (from 159 fewer to 95 fewer)
CHM comprehensive therapy vs. Western medicine treatment				Risk with WM	Risk difference with COM
Visual Analog Scale (VAS) Scale from: 1 to 10	160 (2 RCTs)	⊕⊕⊕◯ Moderate^c,d^	–	The mean visual Analog Scale was **2.94**	MD **0.66 lower** (0.83 lower to 0.48 lower)
Effective rate (Effective rate)	610 (8 RCTs)	⊕⊕◯◯ Low^a,c,d^	**RR 1.10** (1.02 to 1.20)	820 per 1,000	**82 more per 1,000** (16 more to 164 more)
Uric Acid (UA)	510 (7 RCTs)	⊕⊕◯◯ Low^a,c,d^	–	The mean uric Acid was **434.40** μmol/L	MD **40.66 μmol/L lower** (68.21 lower to 13.11 lower)
C-Reactive Protein (CRP)	226 (3 RCTs)	⊕◯◯◯ Very low^a,b,c,d^	–	The mean c-Reactive Protein was **13.55** mg/L	MD **2.47 mg/L lower** (8.29 lower to 3.35 higher)
Erythrocyte Sedimentation Rate (ESR)	366 (5 RCTs)	⊕◯◯◯ Very low^a,b,c,d^	–	The mean erythrocyte Sedimentation Rate was **22.48** mm/h	MD **3.49 mm/h lower** (8.35 lower to 1.38 higher)
White Blood Cell (WBC)	80 (1 RCT)	⊕⊕◯◯ Low^d,e^	–	The mean white Blood Cell was **9.51** ×10^9^/L	MD **1.24** ×**10^9^/L lower** (2.07 lower to 0.41 lower)
Adverse events (AEs)	380 (5 RCTs)	⊕⊕◯◯ Low^c,d^	**RR 0.53** (0.27 to 1.04)	158 per 1,000	**74 fewer per 1,000** (from 115 fewer to 6 more)
CHM plus Western medicine treatment vs. Western medicine treatment				Risk with WM	Risk difference with CHM+WM
Visual Analog Scale (VAS) Scale from: 1 to 10	1351 (17 RCTs)	⊕⊕◯◯ Low^a,c^	–	The mean visual Analog Scale was **2.86**	MD **0.94 lower** (1.2 lower to 0.68 lower)
Effective rate (Effective rate)	2597 (33 RCTs)	⊕⊕⊕◯ Moderate^c^	**RR 1.15** (1.11 to 1.18)	794 per 1,000	**119 more per 1,000** (87 more to 143 more)
Uric Acid (UA)	2597 (33 RCTs)	⊕⊕⊕◯ Moderate^a^	–	The mean uric Acid was **449.73** μmol/L	MD **57.02 μmol/L lower** (66.31 lower to 47.73 lower)
C-Reactive Protein (CRP)	2418 (31 RCTs)	⊕⊕◯◯ Low^a,c^	–	The mean c-Reactive Protein was **17.43** mg/L	MD **4.49 mg/L lower** (5.31 lower to 3.68 lower)
Erythrocyte Sedimentation Rate (ESR)	2418 (31 RCTs)	⊕⊕◯◯ Low^a,c^	–	The mean erythrocyte Sedimentation Rate was **23.94** mm/h	MD **6.06 mm/h lower** (7.22 lower to 4.9 lower)
White Blood Cell (WBC)	342 (5 RCTs)	⊕◯◯◯ Very low^a,b,c^	–	The mean white Blood Cell was **7.95** ×10^9^/L	MD **0.78** ×**10^9^/L lower** (1.88 lower to 0.32 higher)
TCM Syndrome Scoring	1134 (16 RCTs)	⊕⊕◯◯ Low^a,c^	–	The mean TCM Syndrome Scoring was **8.85**	MD **3.18 lower** (4.11 lower to 2.25 lower)
Adverse events (AEs)	1866 (24 RCTs)	⊕⊕◯◯ Low^a,c^	**RR 0.44** (0.33 to 0.59)	94 per 1,000	**41 fewer per 1,000** (from 57 fewer to 18 fewer)
CHM comprehensive therapy plus Western medicine treatment vs. Western medicine treatment				Risk with WM	Risk difference with COM+WM
Visual Analog Scale (VAS) Scale from: 1 to 10	413 (4 RCTs)	⊕⊕◯◯ Low^a,c,d^	–	The mean visual Analog Scale was **3.04**	MD **1.13 lower** (2.14 lower to 0.12 lower)
Effective rate (Effective rate)	580 (6 RCTs)	⊕⊕⊕◯ Moderate^c,d^	**RR 1.11** (1.05 to 1.18)	734 per 1,000	**81 more per 1,000** (37 more to 132 more)
Uric Acid (UA)	457 (5 RCTs)	⊕⊕◯◯ Low^a,c,d^	–	The mean uric Acid was **454.94** μmol/L	MD **86.11 μmol/L lower** (109.72 lower to 62.51 lower)
C-Reactive Protein (CRP)	457 (5 RCTs)	⊕⊕◯◯ Low^a,c,d^	–	The mean c-Reactive Protein was **16.23** mg/L	MD **4.48 mg/L lower** (7.22 lower to 1.74 lower)
Erythrocyte Sedimentation Rate (ESR)	580 (6 RCTs)	⊕⊕◯◯ Low^a,c,d^	–	The mean erythrocyte Sedimentation Rate was **20.05** mm/h	MD **7.04 mm/h lower** (9.33 lower to 4.75 lower)
TCM Syndrome Scoring	147 (2 RCTs)	⊕⊕⊕◯ Moderate^c,d^	–	The mean TCM Syndrome Scoring was **12.02**	MD **3.97 lower** (4.62 lower to 3.33 lower)
Adverse events (AEs)	580 (6 RCTs)	⊕⊕⊕◯ Moderate^c,d^	**RR 1.00** (0.44 to 2.26)	38 per 1,000	**0 fewer per 1,000** (from 21 fewer to 48 more)

The risk in the intervention group (and its 95% confidence interval) is based on the assumed risk in the comparison group and the relative effect of the intervention (and its 95% CI). CI, confidence interval; MD, mean difference; RR, Risk Ratio.

Explanations: a. High statistical heterogeneity, *P* < 0.05. b. The credible interval crosses the "invalid vertical line". c. Funnel plot not symmetrical. d. Small sample size. e. A lack of other studies for comparison.

Bold values indicate statistically significant results (P < 0.05).

### 3.2 Network pharmacology results

#### 3.2.1 Identification of *Tufuling, Huangbo, Bixie* drug targets

From the TCMSP database, 11 active compounds were obtained for *Tufuling*, 3 for *Huangbo* (berberine, kihadalactone A, and coptisine), and 14 for *Bixie*, all meeting the criteria of OB ≥ 30%, DL ≥ 0.18, and BBB > –0.3. (**Table 7**). After target prediction and deduplication, 280 putative targets were obtained for *Tufuling*, 336 for the *Tufuling*-*Huangbo* pair, and 14 for *Bixie*.

**Table 7:** Active ingredients.

Drug	MolId	MolName
Tufuling	MOL013117	4,7-Dihydroxy-5-methoxy-6-methyl-8-formyl-flavan
Tufuling	MOL013119	Enhydrin
Tufuling	MOL001736	(-)-Taxifolin
Tufuling	MOL000358	Beta-sitosterol
Tufuling	MOL004328	Naringenin
Tufuling	MOL000449	Stigmasterol
Tufuling	MOL004567	Isoengelitin
Tufuling	MOL004576	Taxifolin
Tufuling	MOL004580	cis-Dihydroquercetin
Tufuling/Bixie	MOL000546	Diosgenin
Tufuling	MOL000098	Quercetin
Huangbo	MOL001454	Berberine
Huangbo	MOL002636	Kihadalactone A
Huangbo	MOL001458	Coptisine
BiXie	MOL013233	EINECS 213-897-0

#### 3.2.2 Identification of disease targets

**Figure 27:**
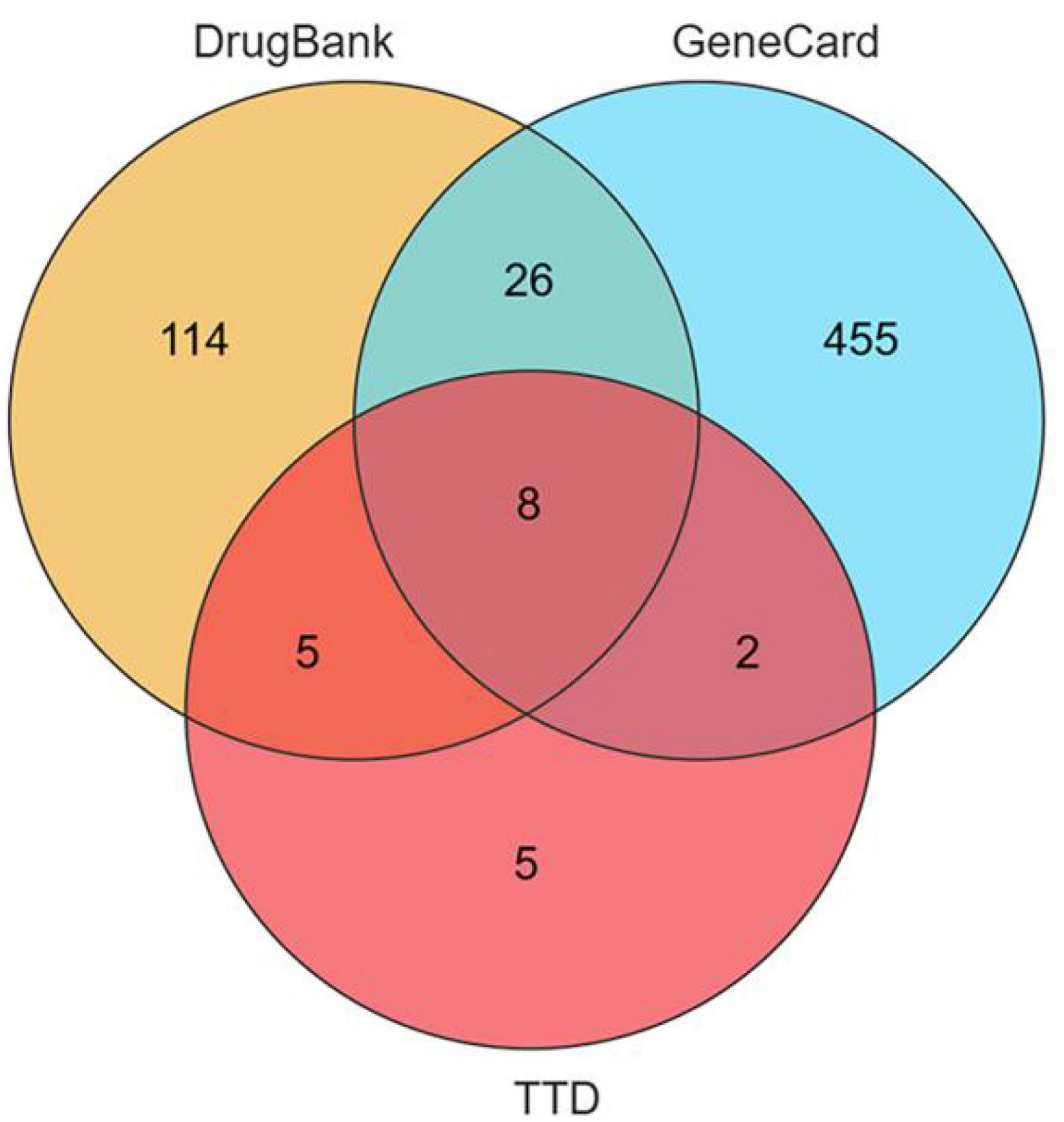
Venn diagram of disease targets.

A total of 153, 491, and 20 targets associated with gout were identified in the DrugBank, GeneCards, and TTD databases, respectively. Following the integration and removal of redundant entries, a non-redundant set of 615 gout-associated targets was established for subsequent analysis (**Figure 27**).

#### 3.2.3 Results of target mapping for the treatment of gout

**Figure 28:**
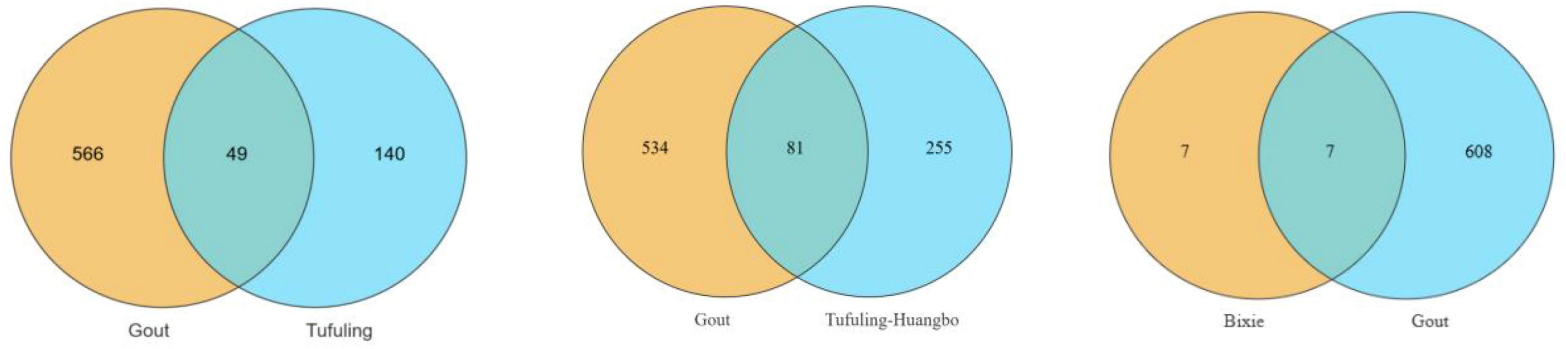
Gout-Tufuling, gout- Tufuling-Huangbo, gout-BixieVenn.

To elucidate the potential mechanism of action of *Tufuling, Bixie* and *Tufuling-Huangbo* in treating gout, the 280 putative *Tufuling* targets, 336 putative *Tufuling*-*Huangbo* pair targets, and 14 for *Bixie* targets were intersected with the 615 gout-associated targets. Intersection analysis yielded 49 overlapping targets for *Tufuling*, 81 for the *Tufulin*g-*Huangbo* pair, and 7 for *Bixie*. Notably, all seven *Bixie* targets were contained within the *Tufuling* target set, indicating a nested pharmacological profile (**Figure 28**).

#### 3.2.4 Results of “component–target–disease” network construction

**Figure 29:**
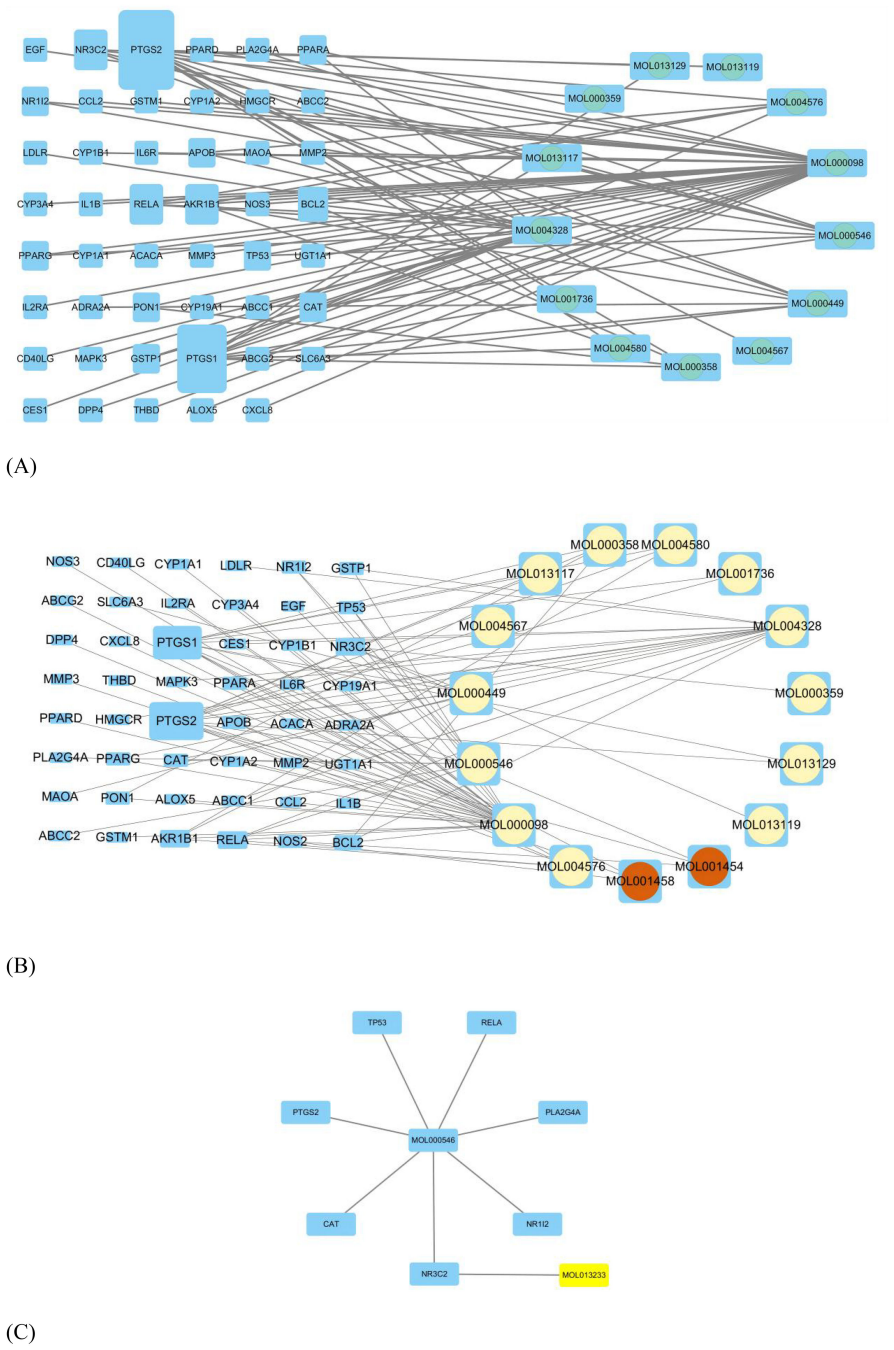
“Component–target–disease” network. **(A)** Tufuling. **(B)** Tufuling-Huangbo. **(C)** Bixie.

A “compound-target-disease” network was constructed on the basis of the active compounds and the intersecting targets via Cytoscape software (v3.10.3). The *Tufuling* network comprised 60 nodes and 84 edges. The *Tufuling*–*Huangbo* network contained 63 nodes and 90 edges, reflecting the additive target coverage of *Huangbo*. The *Bixie* network was substantially smaller, with 9 nodes and 8 edges, consistent with its nested target profile. This network visually encapsulates the multi-compound, multi-target therapeutic characteristic of *Tufuling*, *Tufuling*–*Huangbo* and *Bixie* against gout(**Figure 29**).

#### 3.2.5 Construction results of the intersection gene–protein interaction network

**Figure 30:**
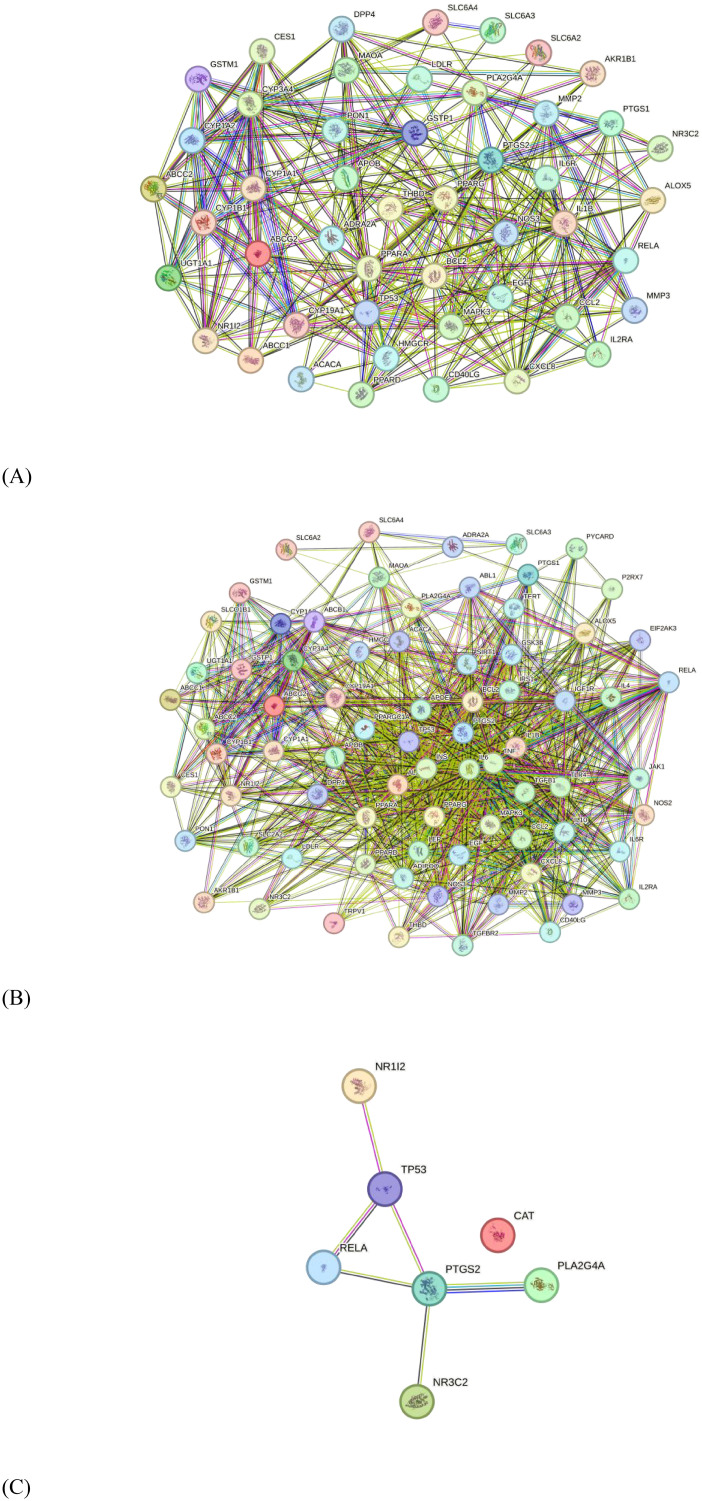
string_hires_image. **(A)** Tufuling. **(B)** Tufuling-Huangbo. **(C)** Bixie.

**Figure 31:**
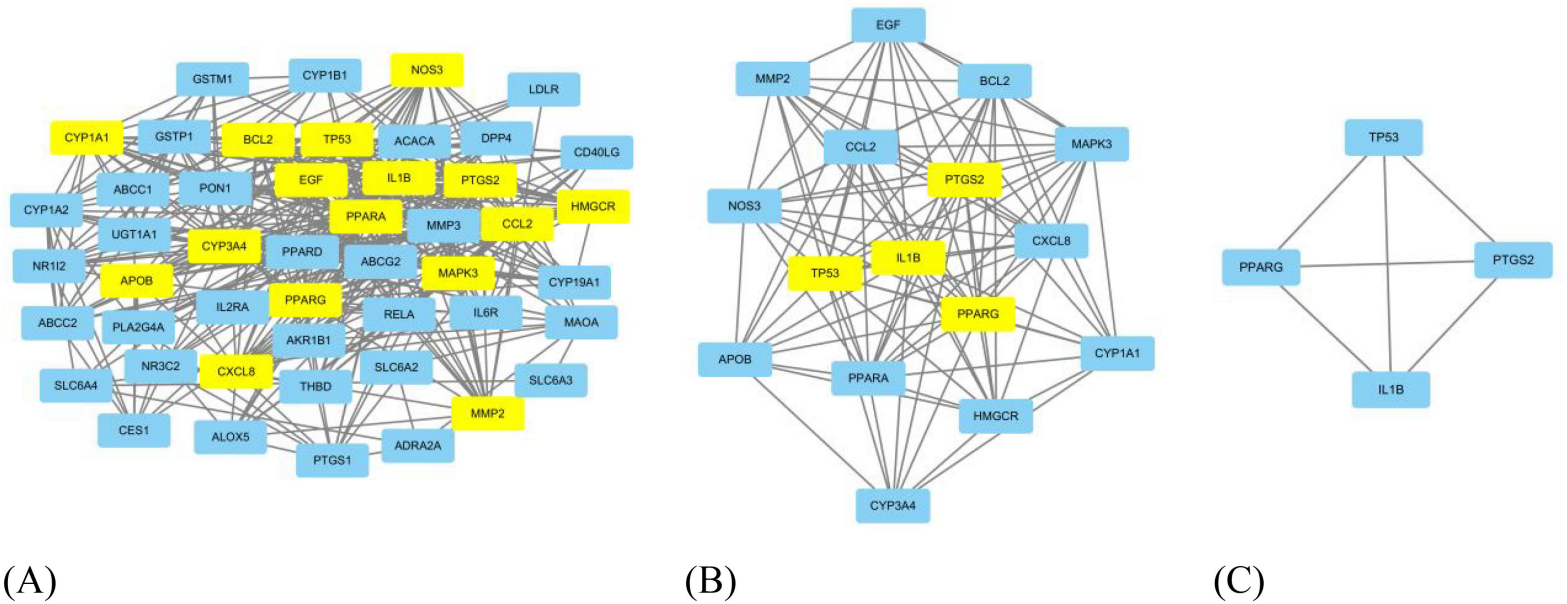
Network 1-3 (Tufuling). **(A)** Network 1, original nodes: 48: edge: 348. **(B)** Network 2, remaining after the first filtering nodes: 16; edge: 100. **(C)** Network 3, remaining after second filtering nodes: 4; edges: 6.

**Figure 32:**
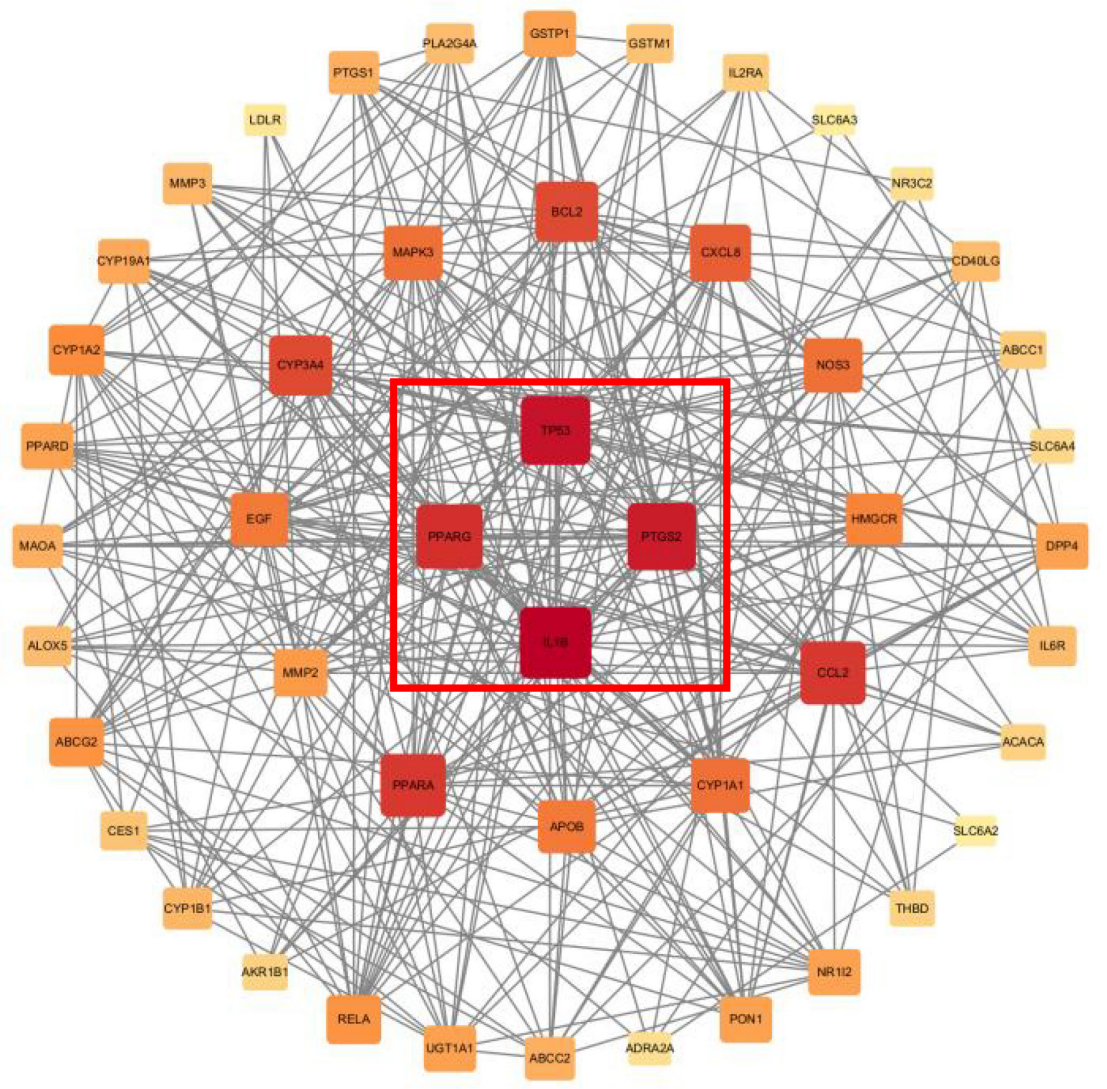
Network 4 (three-circles graph) (Tufuling).

The *Tufuling* PPI network contained 48 nodes and 348 edges (**Figure 30**).Two rounds of CytoHubba (**Table 8**) filtering identified four core targets: PTGS2, IL1B, PPARG, and TP53 (**Figures 31**, **32**).

**Table 8:** Two rounds of filtering on the basis of increasingly stringent criteria (Tufuling).

Parameters for the first filter	Second filter parameters
[1] "filter1,Betweenness: 18.670615375"	[1] "filter2,Betweenness: 1.8156204905"
[1] "filter1,Closeness: 0.56626506"	[1] "filter2,Closeness: 0.882352941"
[1] "filter1,Degree: 14"	[1] "filter2,Degree: 13"
[1] "filter1,Eigenvector: 0.1166605315"	[1] "filter2,Eigenvector: 0.260951906"
[1] "filter1,LAC: 7.8376623375"	[1] "filter2,LAC: 10.74175824"
[1] "filter1,Network: 8.926023976"	[1] "filter2,Network: 12.27373737"

**Figure 33:**
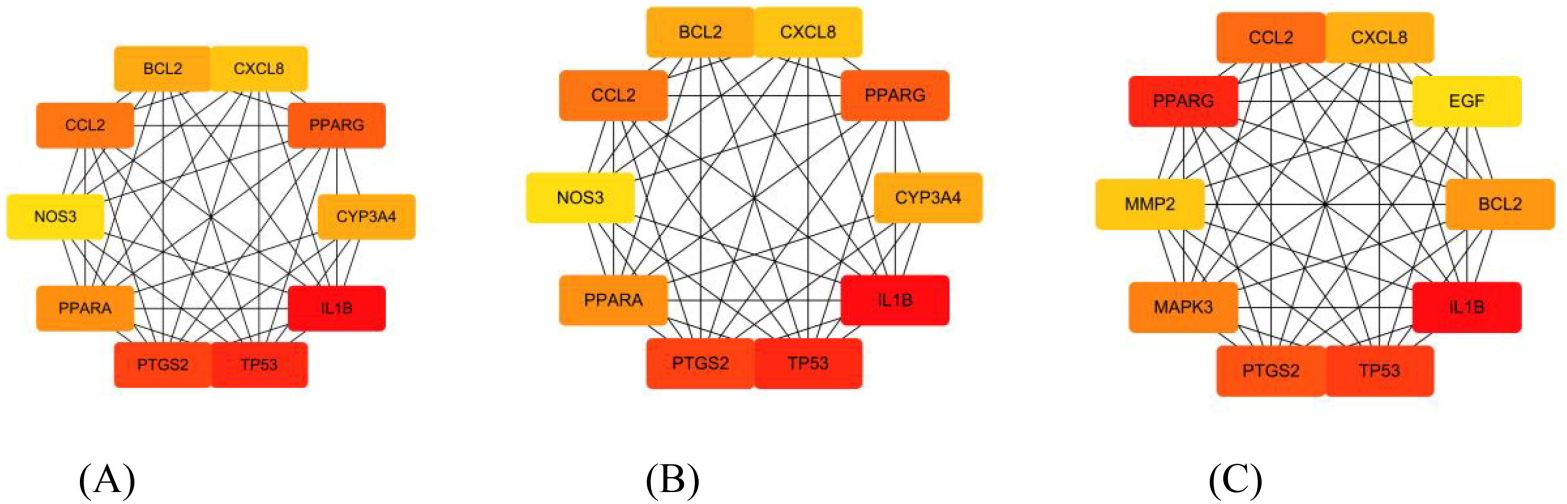
Network1-3 (Tufuling-Huangbo). **(A)** Network 1. **(B)** Network 2. **(C)** Network 3.

**Figure 34:**
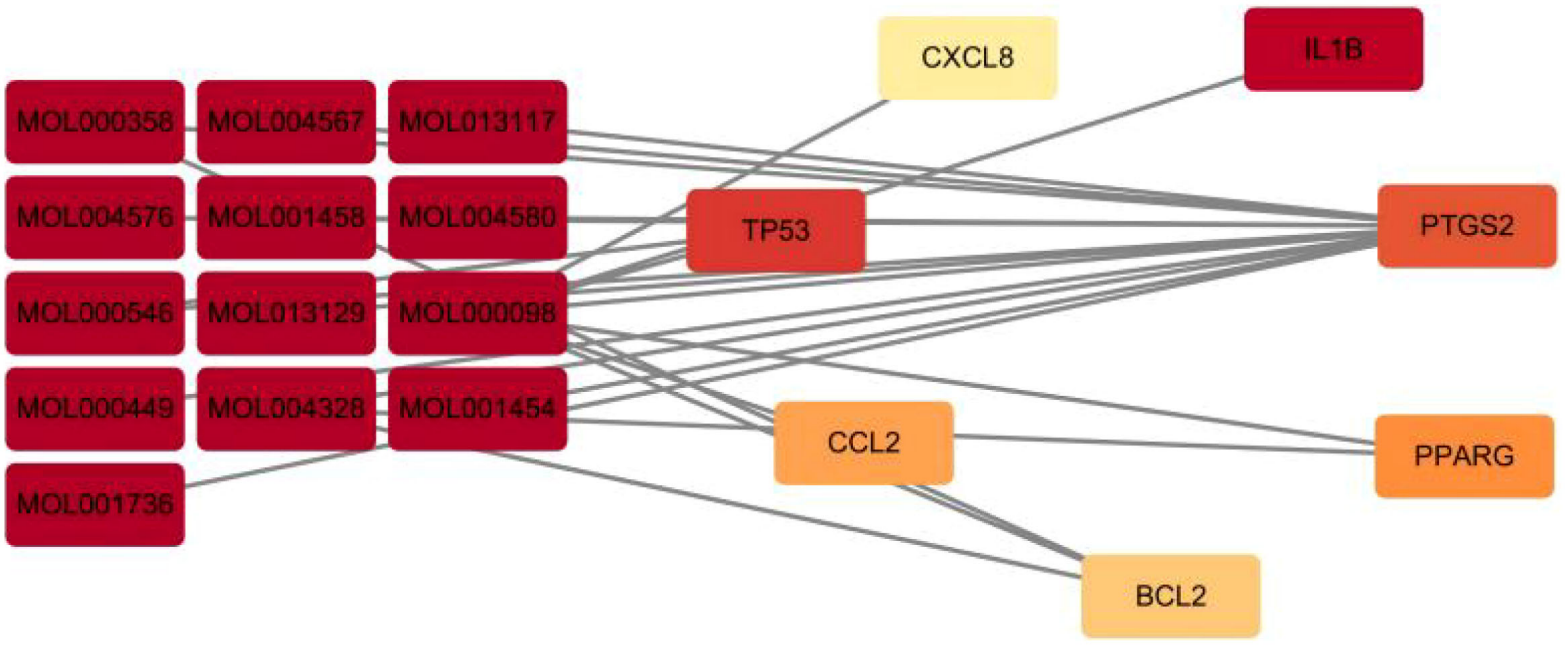
Seven core targets (Tufuling-Huangbo).

The *Tufuling*–*Huangbo* PPI network contained 76 nodes and 1,054 edges (**Figure 30**). Through topological analysis of the PPI network, three algorithms, namely degree centrality, maximal clique, and Maximum Neighborhood Component, were respectively used to rank all targets. The lists of the top 10 targets ranked by each algorithm are presented in (**Table 9**, **Figure 33**). After taking the intersection of the three lists (**Table 9**), a total of 7core targets were obtained, which are IL1B,TP53,PTGS2,PPARG,CCL2,BCL2,CXCL8 (**Figure 34**). The first four overlapped with the *Tufuling* core set; CCL2, BCL2, and CXCL8 emerged as *Huangbo*-specific augmentations.

**Table 9:** Three algorithms were used to rank all targets (Tufuling-Huangbo).

Top 10 in network string_interactions_short.tsv ranked by Degree method			Top 10 in network string_interactions_short.tsv ranked by MNC method			Top 10 in network string_interactions_short.tsv ranked by MCC method		
Rank	Name	Score	Rank	Name	Score	Rank	Name	Score
1	IL1B	33	1	IL1B	33	1	IL1B	2.03E+07
2	TP53	31	2	TP53	31	2	PPARG	2.03E+07
3	PTGS2	30	3	PTGS2	30	3	TP53	2.03E+07
4	PPARG	28	4	PPARG	28	4	PTGS2	2.02E+07
5	CCL2	27	5	CCL2	27	5	CCL2	2.02E+07
6	PPARA	26	6	PPARA	26	6	MAPK3	2.02E+07
7	CYP3A4	25	7	CYP3A4	25	7	BCL2	1.98E+07
7	BCL2	25	7	BCL2	25	8	CXCL8	1.94E+07
9	CXCL8	23	9	CXCL8	23	9	MMP2	1.82E+07
10	NOS3	21	10	NOS3	21	10	EGF	1.10E+07

The *Bixie* PPI network comprised 7 nodes and 6 edges (**Figure 30**). Because all *Bixie* targets were nested within the *Tufuling* target set, formal centrality filtering was not performed.

#### 3.2.6 Results of enrichment analysis of GO and KEGG pathways

Independent GO and KEGG enrichment analyses were not conducted for *Bixie.* All seven intersecting targets identified for *Bixie* were nested within the *Tufuling* target set; as a strict mathematical subset, standalone enrichment of the *Bixie* gene list would yield strictly redundant pathway entries without incremental mechanistic information.

##### 3.2.6.1 Results of the GO enrichment analysis

**Figure 35:**
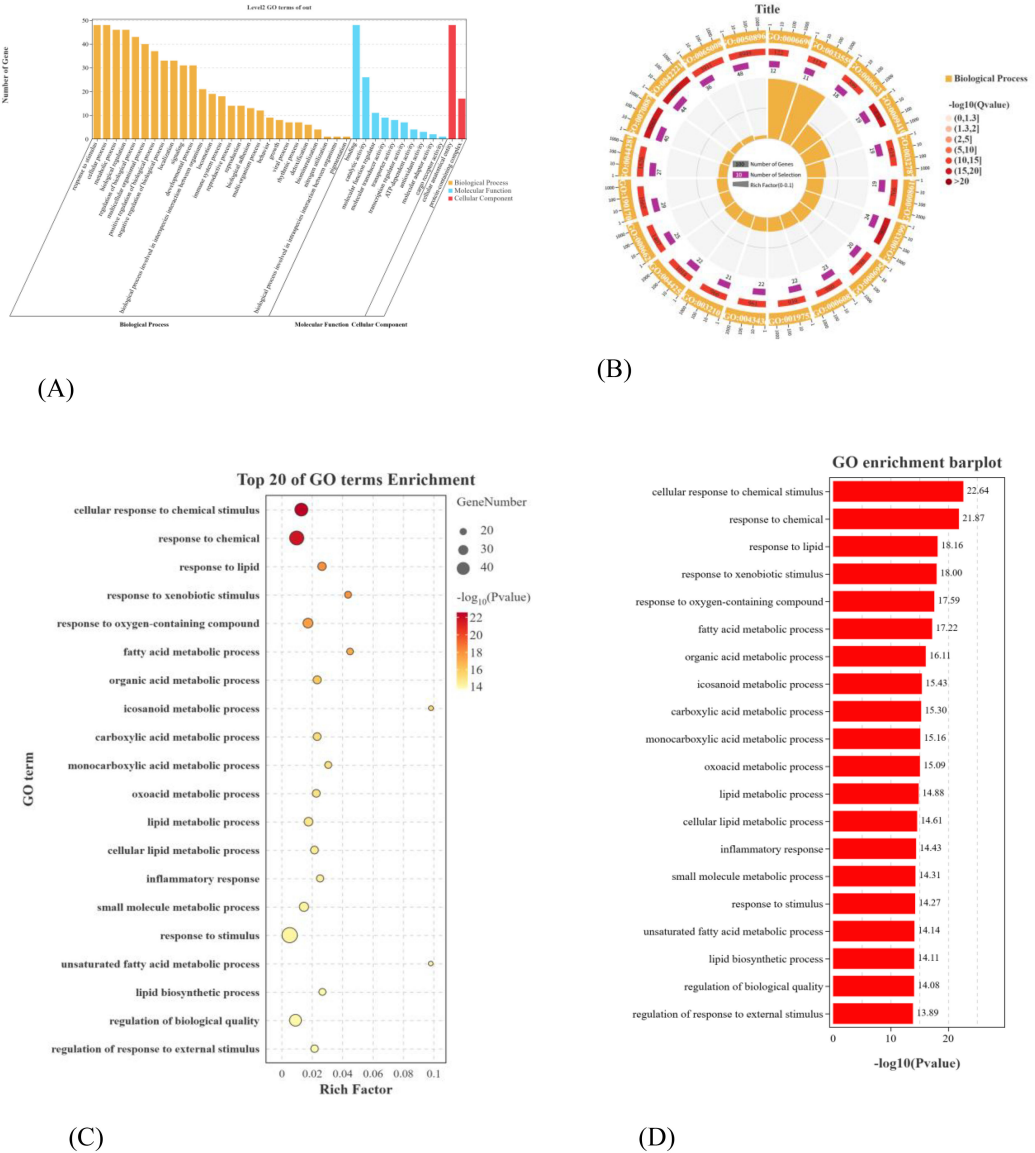
The results of the GO enrichment analysis (Tufuling). **(A)** GO secondary classification bar chart. **(B)** GO enrichment corcle graph. **(C)** GO significance bubble diagram. **(D)** GO significance histogram.

*Tufuling*. GO enrichment of the 48 intersecting targets (after excluding one non-interacting node) yielded 4,082 GO terms; 2,427 met the significance criterion *(P* < 0.05). Biological process (BP) analysis identified 2,031 entries related to stimulus response, metabolic process, and biological regulation. Cellular component (CC) analysis identified 98 entries involving organelles and intracellular membranes. Molecular function (MF) analysis identified 298 entries related to protein binding, enzyme activity, and signaling receptor binding. See **Figure 35** for results.

**Figure 36:**
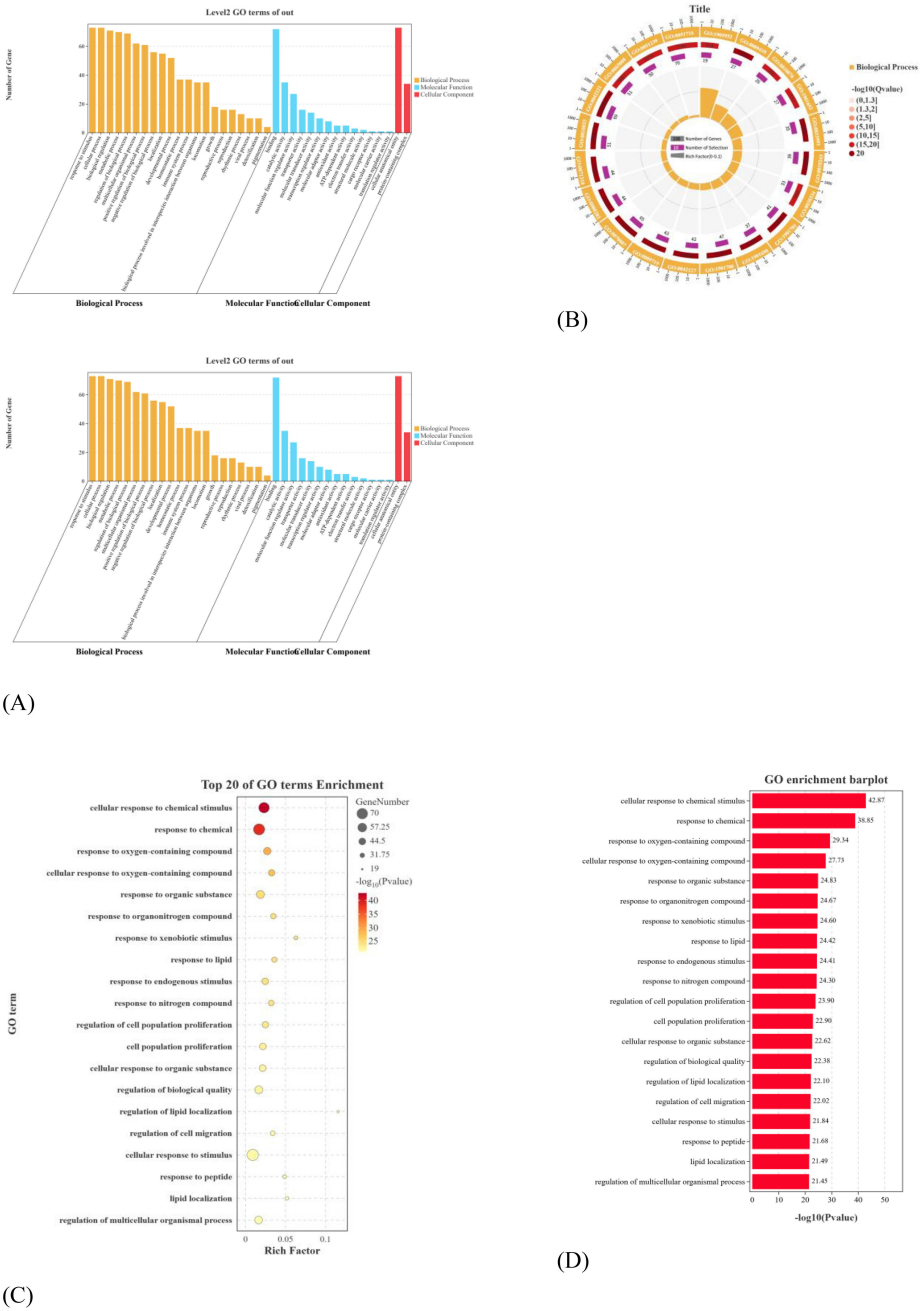
the results of the GO enrichment analysis (Tufuling-Huangbo). **(A)** GO secondary classification bar chart. **(B)** GO enrichment corcle graph. **(C)** GO significance bubble diagram. **(D)** GO significance histogram.

*Tufuling–Huangbo*. Enrichment of the 76 intersecting targets (after excluding five non-interacting nodes) yielded 5,715 GO terms; 3,956 were significant (*P* < 0.05). BP analysis identified 3,394 entries related to stimulus response, metabolic process, and biological regulation. CC analysis identified 146 entries involving protein-containing complexes and cellular anatomical entities. MF analysis identified 416 entries related to catalytic activity, transporter activity, and molecular transducer activity (**Figure 36**).

##### 3.2.6.2 Results of KEGG enrichment analysis

**Figure 37:**
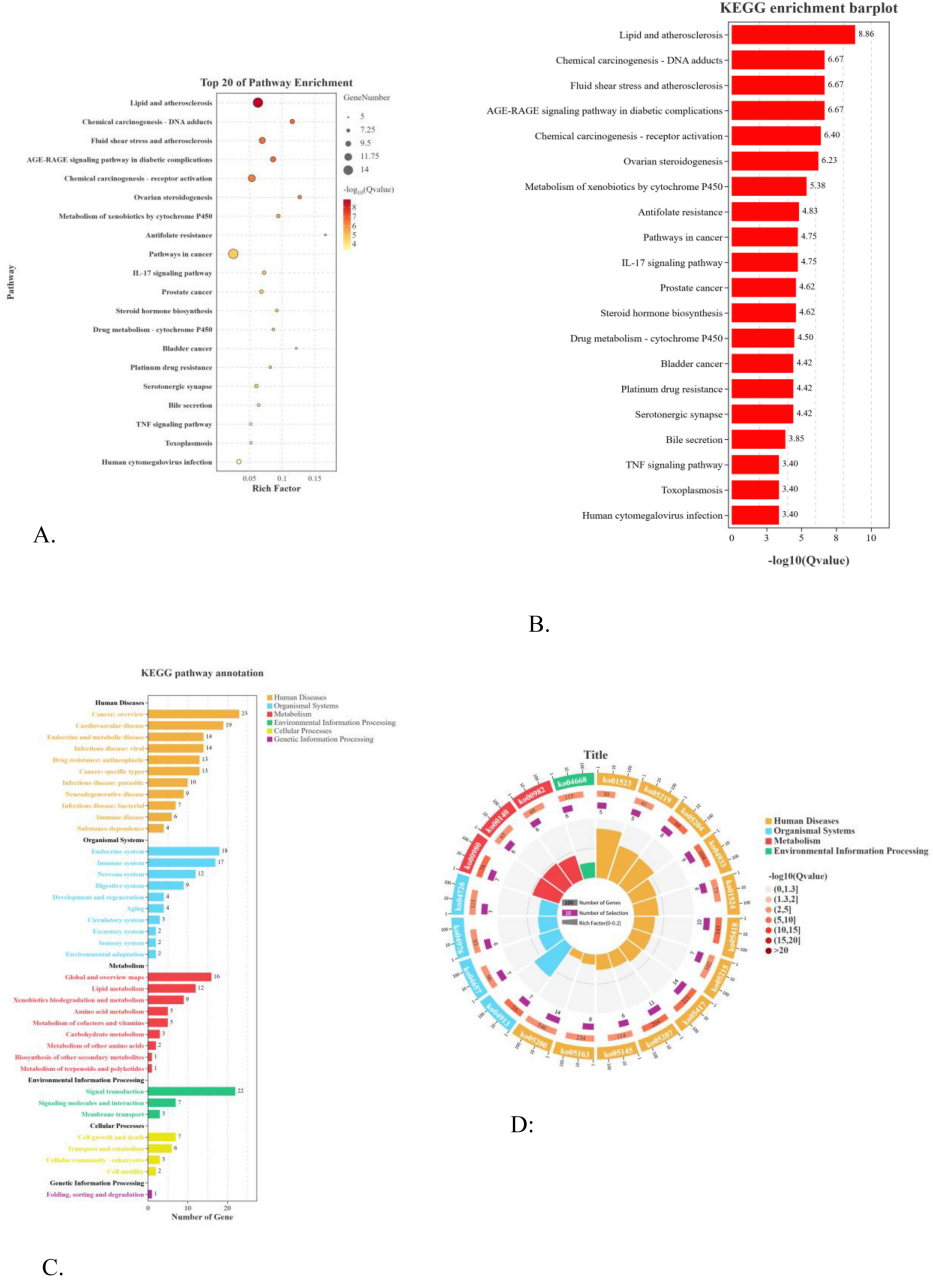
Visualization of the KEGG signaling pathway (Tufuling). **(A)** KEGG significance bubble plot. **(B)** KEGG significance histogram. **(C)** KEGG number statistic graph. **(D)** KEGG enrichment circle diagram.

*Tufuling*. KEGG analysis of the 48 intersecting targets revealed 228 drug-therapy-related pathways, of which 110 disease-related pathways met the significance criterion (*P* < 0.05). Key pathways included metabolic pathways, lipid and atherosclerosis, pathways in cancer, PI3K–Akt signaling, TNF signaling, and IL-17 signaling. See **Table 10** and **Figure 37** for details.

**Table 10:** KEGG signaling pathway (Tufuling).

Pathway name	Number of enriched genes
Metabolic pathways	16
Lipid and atherosclerosis	14
Pathways in cancer	14
PI3K-Akt signaling pathway	8
Metabolism of xenobiotics by cytochrome P450	7
IL-17 signaling pathway	7
Steroid hormone biosynthesis	6
TNF signaling pathway	6
HIF-1 signaling pathway	6
Sphingolipid signaling pathway	6
NF-kappa B signaling pathway	6
MAPK signaling pathway	6
VEGF signaling pathway	4
Cellular senescence	4
JAK-STAT signaling pathway	4
Ras signaling pathway	4
ABC transporters	3
PPAR signaling pathway	3
GnRH signaling pathway	3
AMPK signaling pathway	3

**Figure 38:**
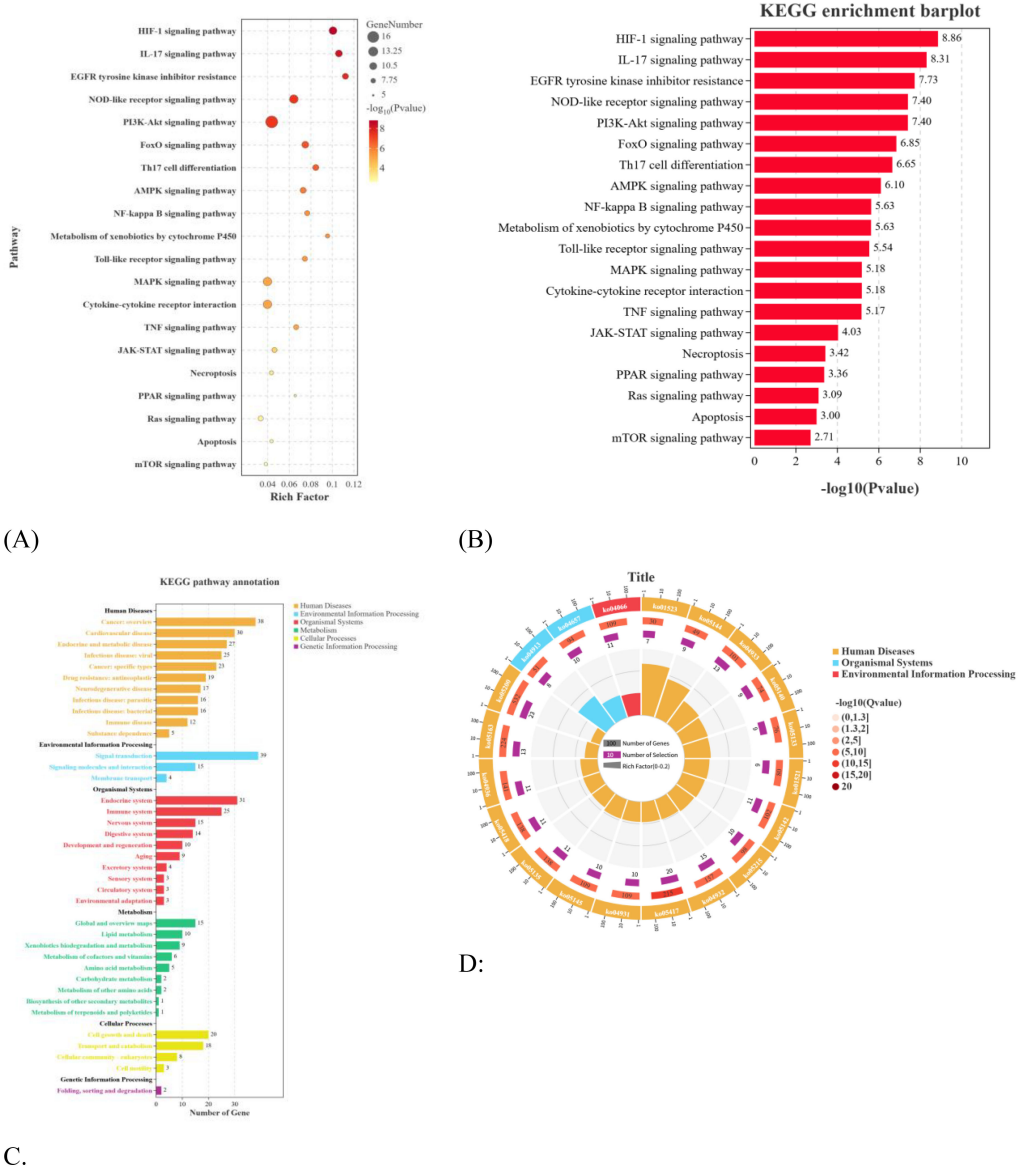
Visualization of the KEGG signaling pathway (Tufuling-huangbo). **(A)** KEGG significance bubble plot. **(B)** KEGG significance histogram. **(C)** KEGG number statistic graph. **(D)** KEGG enrichment circle diagram.

*Tufuling–Huangbo*. Analysis of the 76 intersecting targets revealed 233 pathways, of which 150 were significant (*P* < 0.05). Relative to *Tufuling* alone, the herbal pair showed expanded enrichment in the PI3K–Akt signaling pathway (16 genes), NOD-like receptor signaling pathway (12 genes), MAPK signaling pathway (12 genes), TNF signaling pathway (8 genes), and IL-17 signaling pathway (10 genes). **Table 11** and **Figure 38** provide the detailed information.

**Table 11:** KEGG signaling pathway (Tufuling-huangbo).

Pathway name	Number of enriched genes
PI3K-Akt signaling pathway	16
NOD-like receptor signaling pathway	12
MAPK signaling pathway	12
Cytokine-cytokine receptor interaction	12
HIF-1 signaling pathway	11
IL-17 signaling pathway	10
FoxO signaling pathway	10
EGFR tyrosine kinase inhibitor resistance	9
Th17 cell differentiation	9
AMPK signaling pathway	9
NF-kappa B signaling pathway	8
Toll-like receptor signaling pathway	8
TNF signaling pathway	8
JAK-STAT signaling pathway	8
Ras signaling pathway	8
Metabolism of xenobiotics by cytochrome P450	7
Necroptosis	7
Apoptosis	6
mTOR signaling pathway	6
PPAR signaling pathway	5

##### 3.2.6.3 Molecular docking results

**Figure 39:**
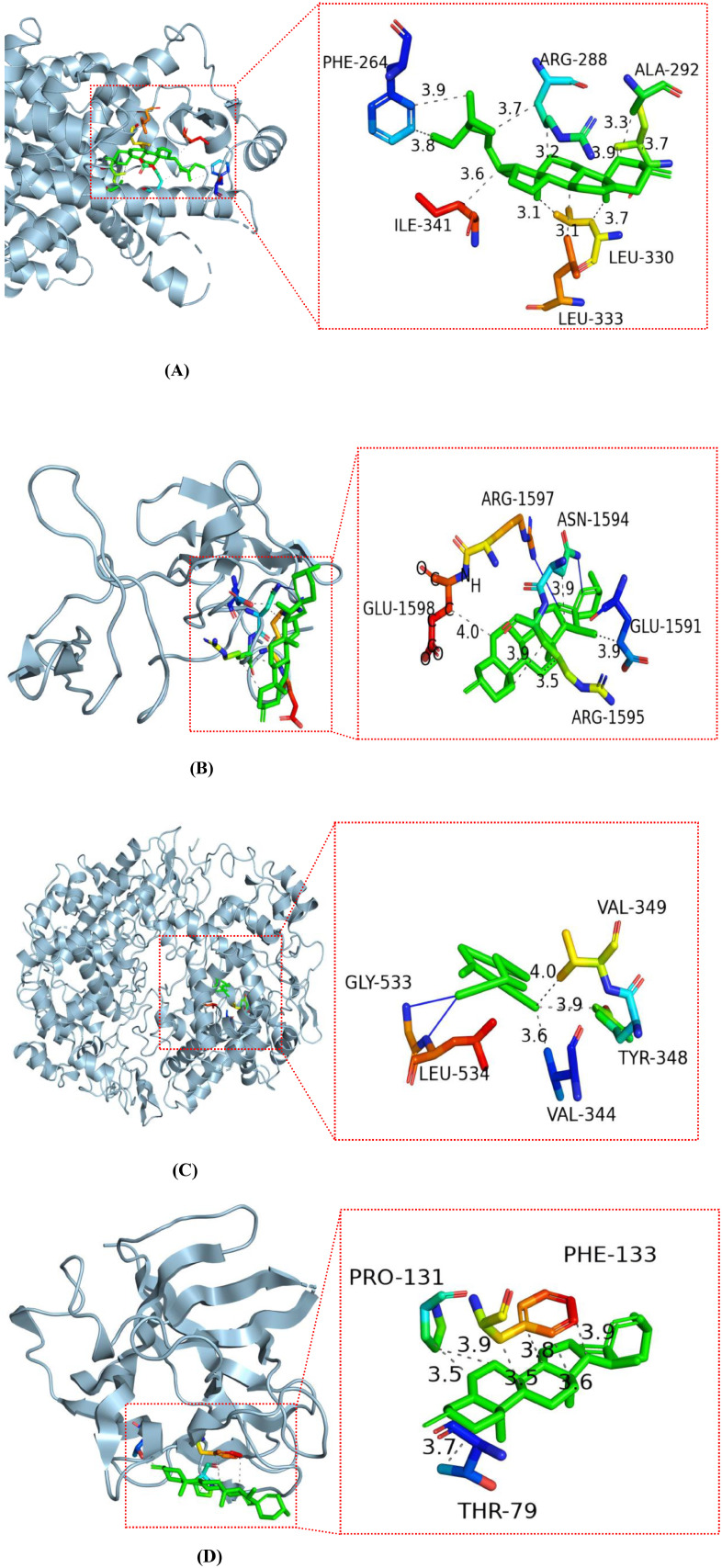
Molecular docking results (Tufuling). **(A)** Molecular docking of PPARG with beta-sitosterol. **(B)** Molecular docking of TP35 with diosgenim. **(C)** Docking plot of PTGS2 with diosgenim molecule. **(C)** Docking pf PTGS2 with diosgenim molecule. **(D)** Docking of IL1B with diosgenim molecule.

*Tufuling.* Diosgenin exhibited the strongest binding affinity for PTGS2 (−11.6 kcal/mol), followed by TP53 (−9.8 kcal/mol) and IL1B (−8.0 kcal/mol). Beta-sitosterol showed high affinity for PPARG (−9.2 kcal/mol) (**Table 12**; **Figure 39**).

**Table 12:** Docking results of core small molecule and core target protein (Tufuling).

Target	PDB ID	Compound	Binding energy	Target	PDB ID	Compound	Binding energy
PPARG	2Q59	4,7-Dihydroxy-5-methoxyl-6-methyl-8-formyl-flavan	-8.8	PTGS2	5F19	4,7-Dihydroxy-5-methoxyl-6-methyl-8-formyl-flavan	-8.8
		Enhydrin	-6.6			Enhydrin	-8.3
		(-)-taxifolin	-8.0			(-)-taxifolin	-8.9
		beta-sitosterol	**-9.2**			beta-sitosterol	-9.2
		naringenin	-8.4			naringenin	-9.5
		Stigmasterol	-8.7			Stigmasterol	-9.7
		isoengelitin	-9.2			isoengelitin	-10.2
		taxifolin	-8.7			taxifolin	-9.0
		cis-Dihydroquercetin	-8.2			cis-Dihydroquercetin	-8.7
		diosgenin	-9.2			diosgenin	**-11.6**
		quercetin	-8.5			quercetin	-9.7
TP53	2G3R	4,7-Dihydroxy-5-methoxyl-6-methyl-8-formyl-flavan	-7.9	IL1B	1HIB	4,7-Dihydroxy-5-methoxyl-6-methyl-8-formyl-flavan	-6.5
		Enhydrin	-6.5			Enhydrin	-6.3
		(-)-taxifolin	-8.2			(-)-taxifolin	-6.9
		beta-sitosterol	-8.8			beta-sitosterol	-7.0
		naringenin	-8.4			naringenin	-7.3
		Stigmasterol	-9.2			Stigmasterol	-7.3
		isoengelitin	-8.6			isoengelitin	-6.9
		taxifolin	-8.1			taxifolin	-7.2
		cis-Dihydroquercetin	-8.0			cis-Dihydroquercetin	-6.9
		diosgenin	**-9.8**			diosgenin	**-8.0**
		quercetin	-8.4			quercetin	-7.2

Bold values indicate statistically significant results (P < 0.05).

**Figure 40:**
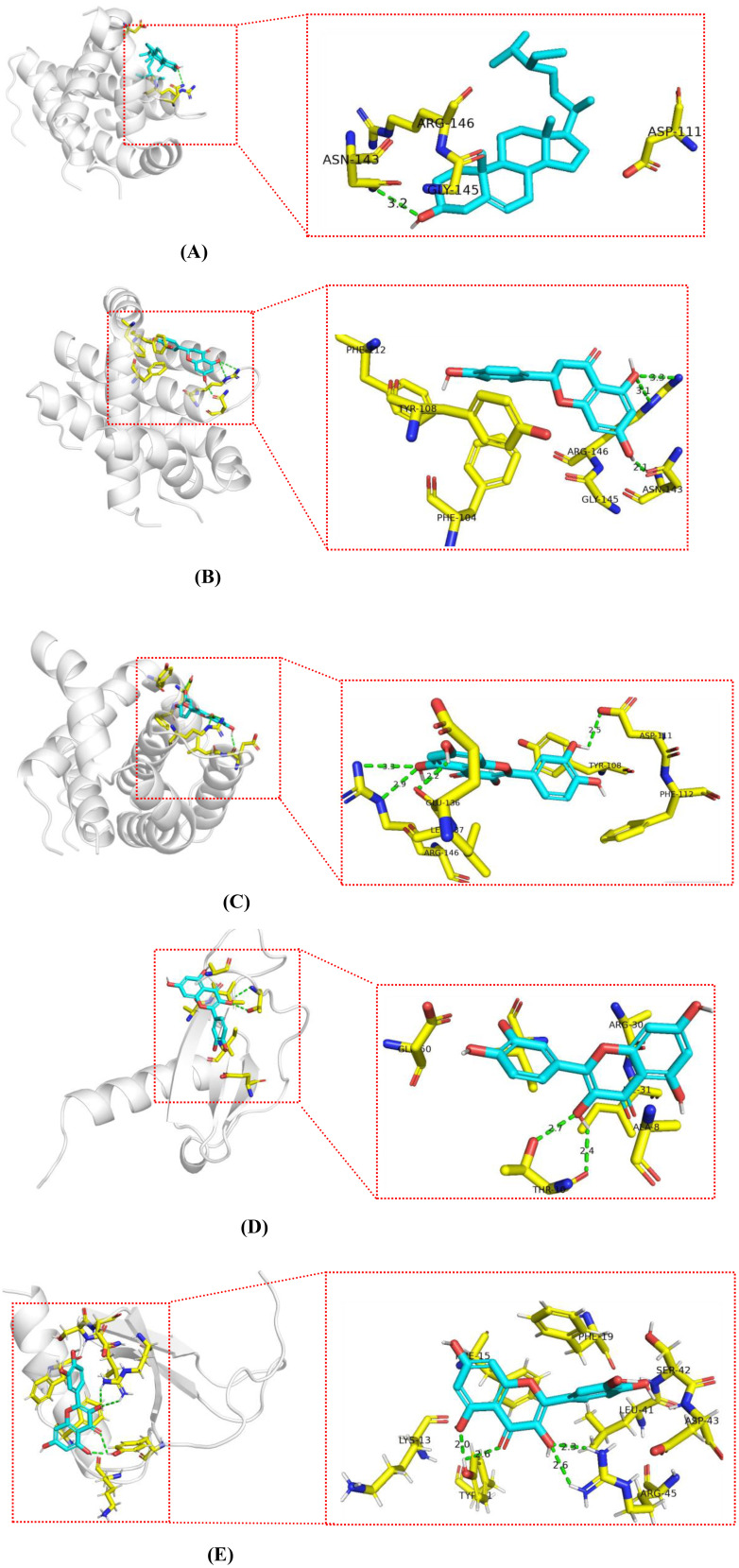
Molecular docking results (Tufuling-Huangbo). **(A)** Molecular docking of BCL2 with beta-sitosterol. **(B)** Molecular docking of BCL2 with naringenim. **(C)** Docking plot of BCL2 with quercetin. **(D)** Docking of CCL2 with quercetin. **(E)** Docking of CXCL8 with quercetin.

*Tufuling–Huangbo*. To avoid redundant reporting of previously examined pairs, only the newly prioritized target from the expanded core list was additionally docked: beta-sitosterol bound to BCL2 with an energy of −7.9 kcal/mol (**Table 13**; **Figure 40**).

**Table 13:** Docking results of core small molecule and core target protein (Tufuling-Huangbo).

Target	PDB ID	Compound	Binding energy
BCL2	5jsn	**beta-sitosterol**	**-7.9**
		naringenin	-6.5
		quercetin	-6.5
CCL2	7so0	quercetin	-5.9
CXCL8	6n2u	quercetin	-6.6

Bold values indicate statistically significant results (P < 0.05).

**Figure 41:**
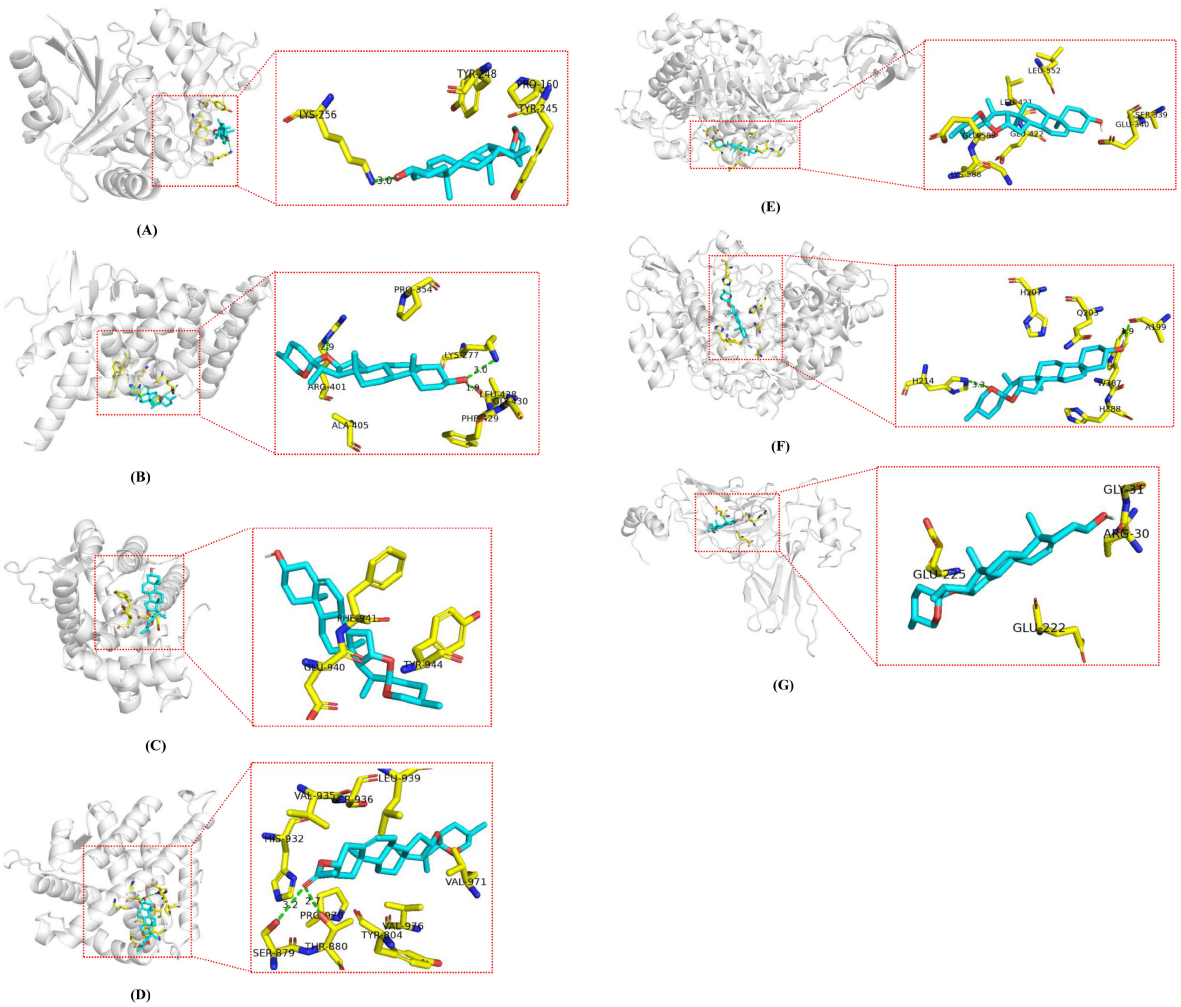
Molecular docking results (Bixie). **(A)** Molecular docking of CAT with diosgenim. **(B)** Molecular docking of NR1I2 with diosgenim. **(C)** Docking plot of NR3C2 with diosgenim. **(D)** Docking of NR3c2 with EINECS_213-897-0. **(E)** Docking of PLA2G4A with diosgenim. **(F)** Docking of PTGS2 with diosgenim. **(G)** Docking of RELA with diosgenim.

*Bixie.* The 2 main components of *Bixie* listed in **Table 6** and the top-ranked 7 key targets screened in the PPI network were subjected to molecular docking by AutoDock Vina software. Diosgenin exhibited the strongest binding affinity for PLA2G4A (−10.3 kcal/mol), followed by PTGS2 (−9.9 kcal/mol). EINECS 213-897–0 bound to NR3C2 with an energy of −8.9 kcal/mol (**Table 14**; **Figure 41**).

**Table 14:** Docking results of core small molecule and core target protein (Bixie).

Target	PDB ID	Compound	Binding energy
**CAT**	1DGF	diosgenin	-7.7
**NR1I2**	6tfi	diosgenin	-8.3
**NR3C2**	4pf3	diosgenin	-8.1
		**EINECS_213-897-0**	-**8.9**
**PLA2G4A**	1cjy	**diosgenin**	**-10.3**
**PTGS2**	5f1a	**diosgenin**	**-9.9**
RELA	1nfi	diosgenin	-7.9

Bold values indicate statistically significant results (P < 0.05).

## 4 Discussion

### 4.1 Summary of principal findings

This study suggests that *Tufuling*-containing traditional Chinese medicine (TCM) formulae may be associated with symptomatic relief during acute gout attacks, particularly in patients with dampness-heat accumulation. A pooled analysis of 56 RCTs indicated that these formulae, whether as adjuncts to Western medicine or as comprehensive TCM regimens, conferred greater improvements in pain (VAS score), inflammation (CRP, ESR), and serum uric acid levels compared with Western medicine monotherapy. These improvements align with TCM concepts of “heat” (inflammation) and “dampness-turbidity” (hyperuricemia). Importantly, meta-analysis of adverse events showed CHM monotherapy reduced AE risk by 75%, CHM+WM by 56%, and COM showed a trend, whereas COM+WM did not differ from WM. All reported AEs were mild to moderate, with no severe events documented; however, 25% of studies did not report AEs, treatment durations were uniformly short (≤14 days), and long-term follow-up data were scarce. The NNT gradient—CHM (8) → COM (14) → CHM+WM (19)—suggests that simpler interventions may be associated with fewer AEs, but these safety signals are preliminary. The certainty of this evidence, assessed via GRADE, ranged from low to moderate, reflecting methodological limitations, risk of bias, and outcome heterogeneity.

Sensitivity analyses showed that the main findings about pain reduction, inflammatory marker suppression (CRP and ESR), and uric acid lowering were mostly strong across intervention comparisons. For example, the benefits of combined CHM+WM therapy on the VAS score, effective rate, UA, CRP, and ESR remained stable even when excluding any single study. Point estimates had minimal changes, and confidence intervals consistently excluded the null value. This reinforces that integrative therapy provides multiple benefits. However, the strength varied for specific outcomes and comparisons. The effect size for UA reduction in some subgroups, like CHM comprehensive therapy alone, was more sensitive to individual studies. This calls for careful interpretation of the exact benefit in those cases.

To identify sources of heterogeneity, meta-regression was conducted for the CHM+WM versus WM comparison. Three clinically informative patterns emerged. First, when western medicine controls already comprised potent anti-inflammatory or urate-lowering combinations, the incremental benefit of adding Chinese herbal medicine became attenuated, reflecting an inherent ceiling effect of polypharmacy. Second, the presence of *Bixie* emerged as a selective enhancer of uric acid reduction; this effect remained independent of baseline metabolic status, supporting its traditional role in separating turbidity. Third, formulas containing *Huangbo* exhibited somewhat attenuated incremental effects on inflammatory and syndrome-based outcomes, suggesting that tailoring prescriptions to individual syndromes may be more conducive to maximizing therapeutic efficacy.

Network pharmacology predicted a hierarchical polypharmacological architecture among the three herbs. *Tufuling* established a foundational network of 49 intersecting targets with four core nodes—PTGS2, IL1B, PPARG, and TP53—providing the primary anti-inflammatory and metabolic framework. The *Tufuling*–*Huangbo* pair expanded this network to 81 targets and seven core nodes; the additional CCL2, BCL2, and CXCL8 augmented inflammatory chemotaxis and apoptotic regulation, while KEGG enrichment demonstrated broadened coverage in PI3K–Akt, MAPK, and NOD-like receptor signaling. This expansion aligns with the TCM concept of *Huangbo* as a minister herb that reinforces the monarch herb’s heat-clearing and dampness-drying actions. In contrast, all seven *Bixie* targets were nested within the *Tufuling* set, precluding independent GO and KEGG enrichment because redundant pathway entries would yield no incremental information. Molecular docking nonetheless predicted a distinct high-affinity interaction between diosgenin and PLA2G4A (−10.3 kcal/mol), a phospholipase A2 isoform central to arachidonic acid metabolism and eicosanoid synthesis, alongside sustained PTGS2 binding (−9.9 kcal/mol). This indicates that *Bixie* refines, rather than expands, *Tufuling*’s network by concentrating its polypharmacology toward lipid-mediator nodes that modulate uric acid homeostasis. This in silico observation converges precisely with the clinical multivariable meta-regression, which identified *Bixie* as the sole significant modifier of serum uric acid reduction (coefficient = −29.30, 95% CI: −45.35 to −13.25, *P* = 0.001). Collectively, these findings support a three-tier synergistic model: *Tufuling* provides the broad foundational network, *Huangbo* expands the target boundary into complementary inflammatory and apoptotic pathways, and *Bixie* refines the network toward arachidonic acid–metabolizing targets that drive urate reduction, thereby mirroring the classical monarch–minister–assistant hierarchy of TCM formulation theory.

### 4.2 Corroboration with TCM theory: “clearing heat and draining dampness”

In TCM, gout during an acute flare is frequently attributed to dampness–heat accumulation, characterized by joint redness, swelling, heat, pain, and systemic signs such as thirst and dark urine. The principle of treatment is to “clear heat, drain dampness, unblock collaterals, and relieve pain.” The findings of this study offer a modern biomedical interpretation of an ancient paradigm.

“Clearing Heat” as Anti-Inflammatory Action: The “heat” component is biomedically linked to acute inflammation. Our network analysis predicted that the effects of *Tufuling* and its bioactive components involve key inflammatory mediators, and a subsequent literature review provided supporting evidence for this prediction (72). Its components are shown or predicted to inhibit PTGS2 (the enzyme producing inflammatory prostaglandins) and the potent cytokine IL-1β, which is a master regulator of gouty inflammation via NLRP3 inflammasome activation (73). This multitarget suppression of the IL-1β/NF-κB/COX-2 axis directly translates to the clinically observed reduction in pain (VAS) and systemic inflammation (CRP, ESR), effectively embodying the “heat-clearing” effect.

“Draining Dampness” as Uric Acid Regulation: The “dampness-turbidity” component corresponds to the metabolic dysfunction of purines leading to hyperuricemia. Our analysis and supporting experimental studies revealed a dual mechanism: inhibition of xanthine oxidase (XOD), a key enzyme in uric acid production, and upregulation of renal transporters, such as ABCG2, to increase uric acid excretion (74, 75). This “inhibit synthesis + promote excretion” strategy provides a concrete pharmacological basis for exploiting the “dampness-draining” and “turbidity-removing” properties of these compounds, corroborating the significant reduction in serum uric acid concentration reported in our meta-analysis.

This combined effect on inflammatory “heat” and metabolic “dampness” shows how TCM takes a holistic, multitarget approach. This differs from the usual single-target focus of conventional drugs.

### 4.3 Dialog with modern pharmacology and experimental evidence

Network pharmacology and molecular docking generated mechanistic hypotheses that require prospective experimental validation (76). Emerging contemporary evidence provides preliminary support for these predicted pathways, though direct biological confirmation remains necessary.

Anti-inflammatory Targets (PTGS2, IL1B, NF-κB Pathway): Previous studies have confirmed that extracts and specific flavonoids from *Smilax glabra* (e.g., astilbin) can significantly inhibit the activation of the NLRP3 inflammasome, reduce IL-1β and TNF-α levels in gout models, and suppress COX-2 expression (72). These findings validate the predicted roles of PTGS2 and IL1B as critical targets.

Metabolic and Anti-inflammatory Targets (PPARG): PPARγ activation exerts anti-inflammatory effects and modulates metabolic homeostasis (77). While direct evidence for the efficacy of *Tufuling* in gout models is still emerging, studies on its components and related herbs suggest that this pathway may contribute to its overall effect, aligning with network predictions.

Uric Acid Metabolism (XOD Inhibition): Recent bioactivity-guided studies have precisely identified specific flavonoids involved in *Tufuling* (e.g., engeletin and isoengeletin) as potent direct inhibitors of XOD, with activity comparable to that of allopurinol (78). This provides strong experimental support for its “dampness-draining” and hypouricemic effects.

Synergy within herbal pairs: The *Tufuling*–*Huangbo* pair added CCL2, BCL2, and CXCL8 to the core set. These targets regulate inflammatory chemotaxis and apoptosis. KEGG analysis showed broadened enrichment in PI3K–Akt, MAPK, and NOD-like receptor signaling. This expansion mirrors the TCM concept of *Huangbo* as a minister herb. It reinforces the monarch herb’s heat-clearing and dampness-drying actions. Berberine, a key alkaloid in *Huangbo*, has been shown to attenuate NLRP3 inflammasome activation and suppress NF-κB signaling in monosodium urate crystal-induced inflammation (79). This experimental evidence supports the network-predicted augmentation of anti-inflammatory capacity.

Refinement by *Bixie*: All seven *Bixie* targets were nested within the *Tufuling* target set. Standalone enrichment was therefore redundant. However, molecular docking revealed a distinct high-affinity interaction. Diosgenin bound PLA2G4A at −10.3 kcal/mol. This phospholipase A2 isoform is central to arachidonic acid metabolism. It drives eicosanoid synthesis and modulates uric acid homeostasis (80). This finding indicates that *Bixie* refines, rather than expands, the *Tufuling* network. It concentrates polypharmacology toward lipid-mediator nodes. This in silico hypothesis aligns with the clinical multivariable meta-regression. Diosgenin, shared by both *Tufuling* and *Bixie*, has been reported to exert hypouricemic effects through dual inhibition of xanthine oxidase and modulation of renal organic ion transporters in recent pharmacokinetic studies (81).

Synergy and Pharmacokinetics: Research shows that the bioavailability of active *Tufuling* compounds can change in certain conditions, like hyperuricemia. This may lead to a stronger focus on target organs (78). This “intelligent” pharmacokinetic behavior highlights the complexity and potential synergy in the multicomponent formula. It explains how different compounds can work together to influence inflammation and metabolism networks.

### 4.4 Clinical implications and strengths

The primary strength of this study lies in its integrative design, which systematically connects syndrome-specific clinical efficacy, in silico mechanistic prediction, and external experimental validation. This approach moves beyond empirical descriptions toward mechanistic explanations. Clinically, the findings suggest that Tufuling-based TCM therapy, especially as an adjunct to conventional treatment, can provide broader symptom control (pain, inflammation, hyperuricemia) with a potentially improved gastrointestinal tolerability profile during acute-gout management. The stability of these results, as demonstrated by the sensitivity analyses, increases confidence in their reproducibility and potential for real-world application. Its multitarget nature may also offer advantages in the management of the complex pathophysiology of gout.

### 4.5 Limitations

This study has several limitations to consider. First, the quality of the included RCTs was often low. Many studies lacked blinding and proper allocation concealment. This could have led to performance and detection bias. All data came from a single healthcare system and population. The way gout presents, along with its comorbidities, diet, and TCM practices, is specific to this context. Thus, the results mainly apply to Chinese clinical settings. Extrapolating to other populations needs validation through multinational trials.

Second, we did extensive subgroup and sensitivity analyses to check for differences and robustness. Still, some outcomes showed residual heterogeneity, like UA in monotherapy analyses. Sensitivity analyses showed that key outcomes, such as effective rate and pain reduction, were stable. However, certain results, like UA reduction in the CHM therapy group and ESR in various comparisons, were more sensitive to specific studies. This suggests that besides measured factors—like control drug type and baseline UA—other unmeasured variables, such as differences in herbal formula composition, treatment duration, or patient adherence, might explain the variability. Univariable and multivariable meta-regression explored *Tufuling* dose, herbal combinations, treatment duration, baseline UA, comparator class, and randomization quality. *Bixie* was the sole significant effect modifier for UA, explaining 31.76% of variance; baseline UA independently contributed in the multivariable model (adjusted R² = 46.05%). Residual heterogeneity likely stemmed from unmeasured variables including disease duration, dietary control, and the complexity of 196 co-administered herbs. Future trials should address these factors.

Third, while we backed network predictions with published evidence, these remain indirect validations. Direct *in vitro* and *in vivo* studies on specific compound-target interactions, like diosgenin and TP53 or diosgenin–PLA2G4A in gout models in gout models, are needed for solid proof.

Fourth, the complexity of herbal formulas makes it hard to separate the effects of TCM from other herbs. It also complicates identifying all possible synergistic interactions. Finally, our analysis focused only on the acute phase and dampness–heat pattern. Efficacy in chronic gout or other TCM patterns needs separate investigation. Safety conclusions should be interpreted cautiously 25% of the included trials did not report AEs, introducing potential reporting bias; zero-event studies required continuity correction; all treatment durations were ≤14 days; and no long-term follow-up data were available to assess delayed or chronic toxicity. Consequently, the observed safety signals are preliminary and require confirmation in RCTs with standardized AE reporting and extended follow-up. Future RCTs with extended follow-up are warranted.

## 5 Conclusion and future perspectives

This study suggests that *Tufuling*-containing TCM formulae may be associated with symptomatic improvements in acute gout with dampness-heat accumulation. It helps relieve pain, inflammation, and high uric acid levels. Network pharmacology and molecular docking generated hypotheses implicating multi-target modulation of inflammatory and metabolic pathways (e.g., IL1B, PTGS2); however, these computational predictions require further experimental validation.

Future research should focus on: 1) Conducting larger, well-designed RCTs with standardized TCM diagnosis and longer follow-up to check long-term results and flare prevention. 2) Employing experimental techniques, such as gene editing and spatial metabolomics, to validate the predicted roles of core targets and pathways in gout models. 3) Investigating the synergy between the “heat-clearing” and “dampness-draining” components in *Tufuling* and its classic formulas, particularly the *Tufuling*–*Huangbo* core pair, to develop better, evidence-based botanical prescriptions.

## Data Availability

The original contributions presented in the study are included in the article/supplementary material. Further inquiries can be directed to the corresponding author.
